# Bifunctional thiourea-catalyzed asymmetric [3 + 2] annulation reactions of 2-isothiocyanato-1-indanones with barbiturate-based olefins

**DOI:** 10.3762/bjoc.18.3

**Published:** 2022-01-04

**Authors:** Jiang-Song Zhai, Da-Ming Du

**Affiliations:** 1 School of Chemistry and Chemical Engineering, Beijing Institute of Technology, No.5 Zhongguancun South Street, Beijing 100081, People’s Republic of China

**Keywords:** asymmetric catalysis, cyclization reaction, Michael addition, one-pot three-component reaction, spirobarbiturates

## Abstract

Bifunctional thiourea-catalyzed asymmetric [3 + 2] annulation reactions of 2-isothiocyanato-1-indanones with barbiturate-based olefins have been developed to afford chiral dispiro[indene-pyrrolidine-pyrimidine]s. Through this strategy, the target products could be obtained in good to excellent yields with excellent stereoselectivities. In addition, the synthetic utility was verified through a gram-scale synthesis, one-pot three-component reactions and further transformation experiments of the products.

## Introduction

**Figure 1:**
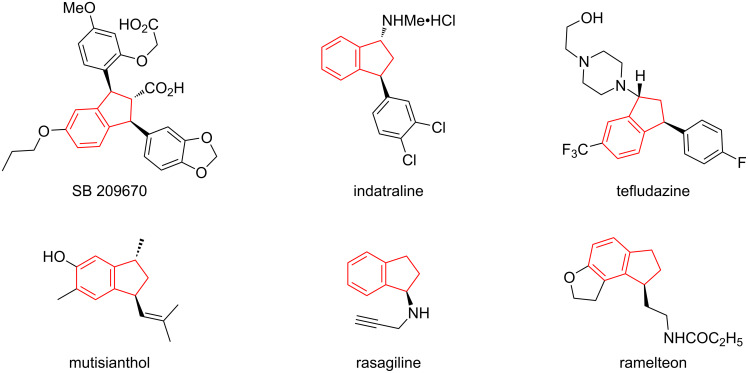
Selected examples of natural products and drugs possessing the indane scaffold.

**Scheme 1:**
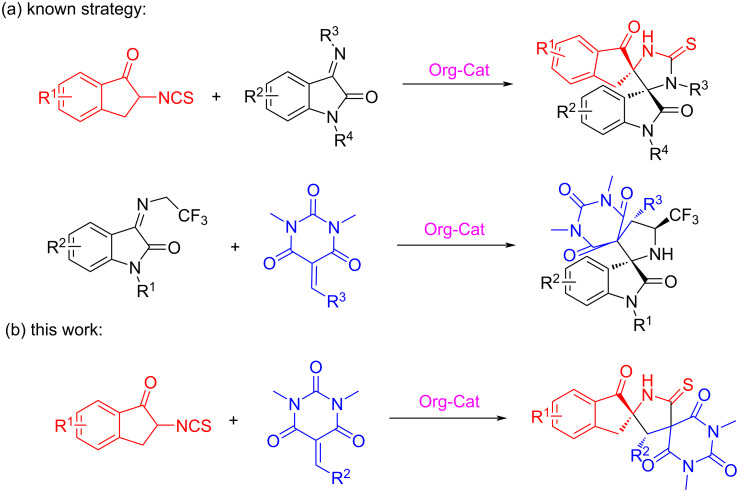
Known strategies and conceptual advance of this contribution.

Indane scaffolds exist in various biologically active natural products and pharmaceutical compounds with antipsychotic and antifungal activities, such as SB 209670, indatraline, tefludazine, mutisianthol, rasagiline, and ramelteon (Figure 1) [1–5]. Therefore, this structural motif has attracted great attention of researchers in the field of synthetic organic chemistry and pharmaceutical chemistry all over the world. In the previous few decades, a large number of strategies emerged to construct heterocyclic compounds with this skeleton or similar ones [6–10], aiming to explore biological activity and medicinal value conveniently and comprehensively. However, as we know, the construction of these compounds is mostly carried out through transition-metal-catalyzed cyclization reactions [11–14], whereas strategies using bifunctional chiral thiourea catalysts are rarely reported. In 2018, Du's group reported a novel cascade reagent with the indane framework, namely, 2-isothiocyanato-1-indanone (Scheme 1a) [15], but research on its participation in the construction of chiral compounds has been relatively low [16–17].

**Figure 2:**
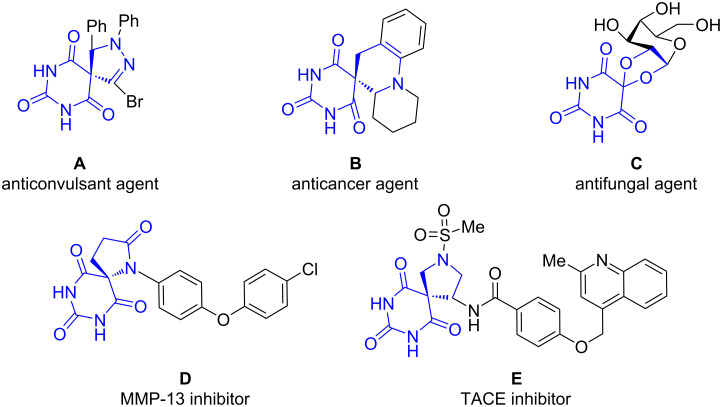
Selected examples of bioactive spirobarbiturates.

On the other hand, as a kind of vital spiroheterocyclic derivatives, spirobarbiturates show a wide range of significant pharmacological and physiological activities in the medical and biological fields (Figure 2) [18–21]. For instance, compound **A** displays anticonvulsant activity and compound **C** can be used as an antifungal agent [22–23]. This impels the quest to develop a series of synthons or new methodologies to construct the spirobarbiturates with diverse structures. In recent years, good progress has been achieved in the construction of racemates of spirobarbiturates and the enantioselective synthesis [24–29], but only limited progress has been made in the construction of bispirobarbiturates [30–31]. In 2019, for example, An and co-workers reported an asymmetric Michael/Mannich [3 + 2] cycloaddition reaction between *N*-(2,2,2-trifluoroethyl)isatin ketimines and barbiturate-based olefins (Scheme 1a) [32]. Based on the current knowledge, the construction of dispirobarbiturates containing the indane skeleton has not been reported yet.

In light of the prominent bioactivities and the pharmacological activity of the above two framework compounds, the combination of these two species may be potential drug candidates. Therefore, it is of great significance to develop a new strategy to construct a series of spirobarbiturates derived from indanone. Combining current researches of these two compounds, we report the first organocatalytic asymmetric Michael addition/cyclization reaction between barbituric acid-derived olefins and indanones (Scheme 1b). Under the action of the bifunctional thiourea catalyst, a series of target products in excellent yields with excellent stereoselectivities can be obtained under mild conditions in this reaction. Notably, this protocol provides direct access to indanone-derived spirobarbiturates, which are difficult to access with other methods.

## Results and Discussion

To verify the feasibility of the reaction, the domino Michael addition/cyclization reaction of 2-isothiocyanato-1-indanone (**1a**) and barbiturate-based olefin **2a** was used as a model reaction, which was carried out in dichloromethane (DCM) with 5 mol % quinine-derived squaramide **C1** at room temperature. The results are summarized in Table 1. We were pleased to find that the domino Michael addition/cyclization reaction could complete in the presence of 5 mol % **C1** at room temperature in 12 h providing the desired product **3aa** in 55% yield with excellent stereoselectivity (>20:1 dr, 97% ee) (Table 1, entry 1). Due to the excellent stereoselectivity of the target product **3aa**, the reaction conditions were further optimized to increase its yield.

**Table 1:** Optimization of the reaction conditions^a^.

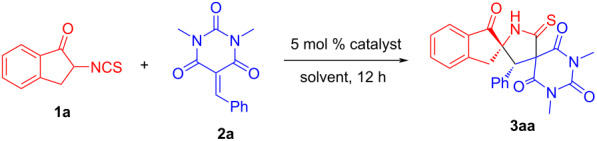					

Entry	Solvent	Catalyst	Yield^b^ (%)	dr^c^	ee^d^ (%)

1	DCM	**C1**	55	>20:1	97
2	DCM	**C2**	57	>20:1	89
3	DCM	**C3**	61	>20:1	97
4	DCM	**C4**	81	>20:1	97
5	DCM	**C5**	79	>20:1	96
6	DCM	**C6**	65	>20:1	94
7	DCM	**C7**	84	>20:1	94
8	DCM	**C8**	82	>20:1	96
9	CHCl_3_	**C4**	83	>20:1	94
10	PhMe	**C4**	80	>20:1	95
11	THF	**C4**	81	>20:1	86
12	MeCN	**C4**	79	>20:1	94
13	DCE	**C4**	72	>20:1	96
14	dioxane	**C4**	81	>20:1	96
15	EtOAc	**C4**	74	>20:1	93
16^e^	DCM	**C4**	76	>20:1	94

*^a^*Unless otherwise specified, the reactions were carried out with **1a** (0.12 mmol), **2a** (0.10 mmol) and catalyst (5 mol %) in solvent (1.0 mL) at room temperature for 12 h. ^b^Isolated yield after column chromatography purification. ^c^Determined by ^1^H NMR analysis. ^d^Enantiomeric excess (ee) was determined by HPLC analysis. ^e^2.5 mol % catalyst was used and reaction time was 18 h.

**Figure 3:**
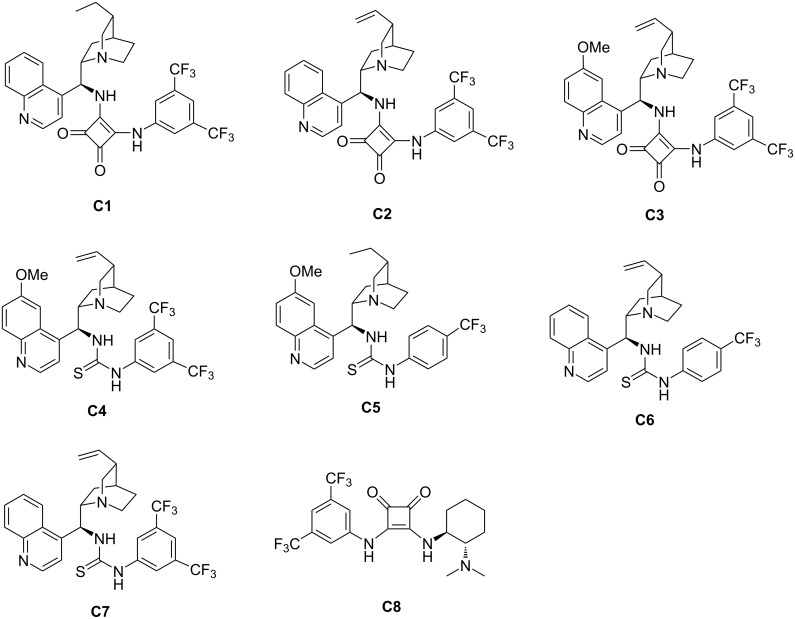
The screened organocatalysts.

Subsequently, a number of organocatalysts (Figure 3) were evaluated for this domino process (Table 1, entries 2–8). From the experimental results, it was found that the yield of product did not increase significantly with the cinchona alkaloid-derived squaramide catalysts (Table 1, entries 2 and 3). Consequently, we decided to explore the effects of different types of catalysts on the reaction. Through experiments, it can be found that thiourea catalysts (**C4**–**C7**) can catalyze the reaction to obtain higher yields while keeping the stereoselectivity basically unchanged. Then we chose the **C4** catalyst with the best reaction effect as the optimal catalyst to explore the influence of other reaction conditions such as solvent type and catalyst loading on the reaction (Table 1, entries 9–16). The experimental results show that the solvent has a non-negligible effect on the reaction and dichloromethane (DCM) has the best reaction effect in the annulation system (Table 1, entries 9–15). Hereafter, we tried to reduce the catalyst loading to further improve the reaction yield and enantioselectivity, but it did not meet our expectations (Table 1, entry 16). Taking into account the ease of operation of the experiment and for economic reasons, we did not explore the effect of increasing the catalyst loading and changing the reaction temperature on the reaction. Based on the above evaluation, we finally selected 2-isothiocyanato-1-indanones **1** and barbiturate-based olefins **2** with a molar ratio of 1.2:1 to react for 12 h at room temperature in DCM using 5 mol % of catalyst **C4** as the optimum reaction conditions.

**Scheme 2:**
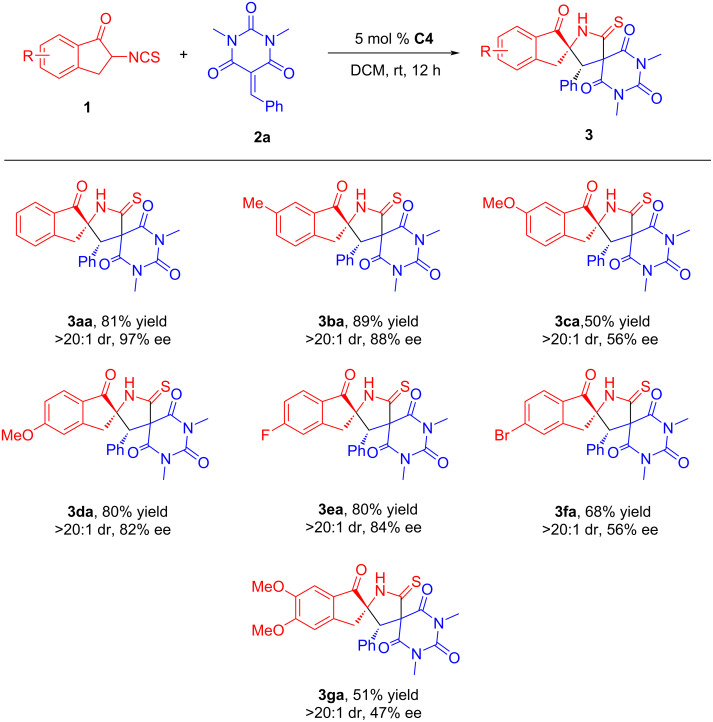
Substrate scope of 2-isothiocyanato-1-indanones. The reactions were carried out with **1** (0.12 mmol), **2a** (0.10 mmol), and catalyst (5 mol %) in solvent (1.0 mL) at room temperature for 12 h. The yields refer to the isolated products after column chromatography. The diastereoisomeric ratios (dr values) were determined by ^1^H NMR spectroscopy and the enantiomeric excess (ee) values were determined by HPLC analysis.

With the optimum reaction conditions established, we then commenced to probe the substrate scope and limitations of this reaction. As summarized in Scheme 2, a variety of 2-isothiocyanato-1-indanones **1** were firstly tested under the optimized conditions. When a methyl substituent is located at the 6-position of the indanone, the reaction yield of the product **3ba** was higher than that of the model reaction, but the enantioselectivity was partially reduced. When the 5-position of the indanone was substituted by either F or a MeO group, the yield remained nearly unchanged, however, the enantioselectivity was slightly reduced. On the other hand, when the 5-position of the indanone was substituted by Br, the 6-position was substituted by a MeO group, and the 5 and 6-positions are simultaneously substituted by a MeO group, the yields and stereoselectivities of the reactions significantly dropped. This indicates that the position of the substituent has a great influence on the reaction. Gratifyingly, the diastereoselectivities of the reactions were maintained.

**Scheme 3:**
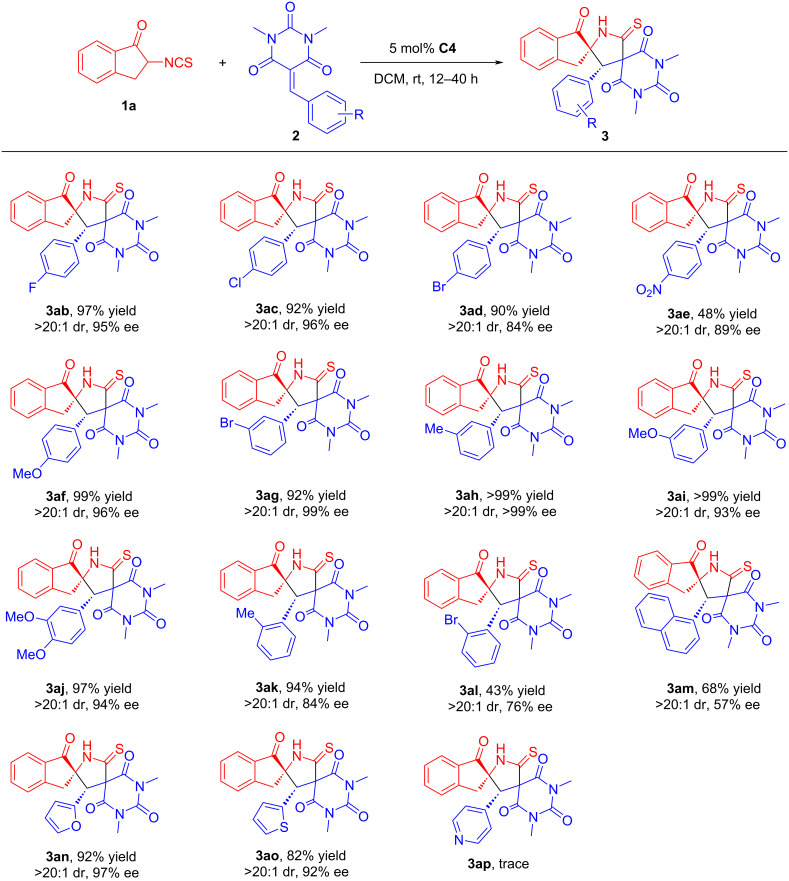
Substrate scope of barbiturate-based olefins. The reactions were carried out with **1a** (0.12 mmol), **2** (0.10 mmol) and catalyst **C4** (5 mol %) in solvent (1.0 mL) at room temperature for 12–40 h. The yields refer to isolated products after column chromatography. The diastereoisomeric ratios (dr values) were determined by ^1^H NMR spectroscopy and the enantiomeric excess (ee) values were determined by HPLC analysis.

To further explore the generality of this reaction, structurally diverse barbiturate-based olefins **2** were examined under the standard conditions by reacting with **1a**. As shown in Scheme 3, in addition to substrates **3ae** and **3al**, it appeared that the reaction could well tolerated the presence of electron-donating and electron-withdrawing groups on the benzene ring of substrates **2**, and afforded most of the products **3** in excellent chemical yields (90 to >99%) and stereoselectivities (>20:1 dr, 84–>99% ee). Possibly due to the influence of steric hindrance, reactions involving substrate **2** with *ortho-*substitution on the benzene ring has lower yields and worse enantioselectivities than those with *meta*-substitution and *para*-substitution. Meanwhile, the enantioselectivities of the products **3am** and **3ao** were partially decreased when the R^1^ group was substituted by naphthyl and thienyl, respectively. It was a good result that R^1^ was substituted by furyl. Unfortunately, when the R^1^ was a pyridyl group, the product was obtained in trace amounts. This may be partly related to the poor solubility of this substrate.

**Figure 4:**
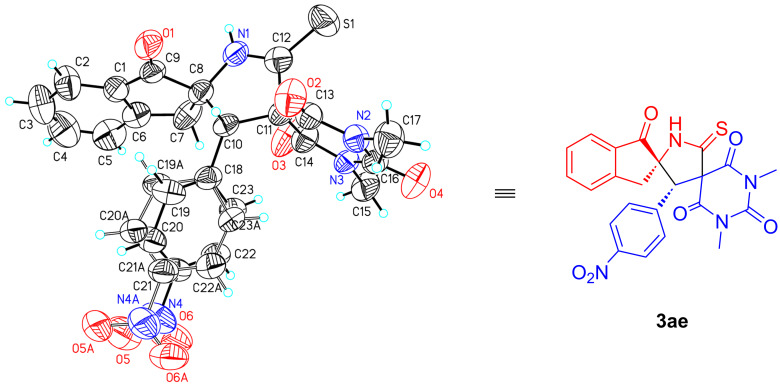
X-ray crystal structure of **3ae** (displacement ellipsoids are drawn at the 50% probability level).

The absolute configuration of the chiral product **3ae** was unambiguously identified on the basis of single-crystal X-ray diffraction analysis as (2*S*,3'*S*) (Figure 4) [33]. The configurations of the other products were assigned by analogy to **3ae**.

**Scheme 4:**
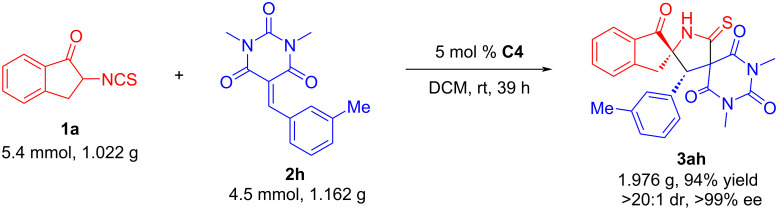
Gram-scale synthesis of **3ah**.

In order to further prove the application value of this asymmetric domino Michael addition/cyclization reaction, a gram-scale experiment was performed under the optimized conditions. As exemplified in Scheme 4, the desired dispiro[indene-pyrrolidine-pyrimidine] **3ah** could be obtained in 94% yield with excellent stereoselectivity (>20:1 dr, >99% ee), which indicated this strategy shows promising prospects for mass production.

**Scheme 5:**
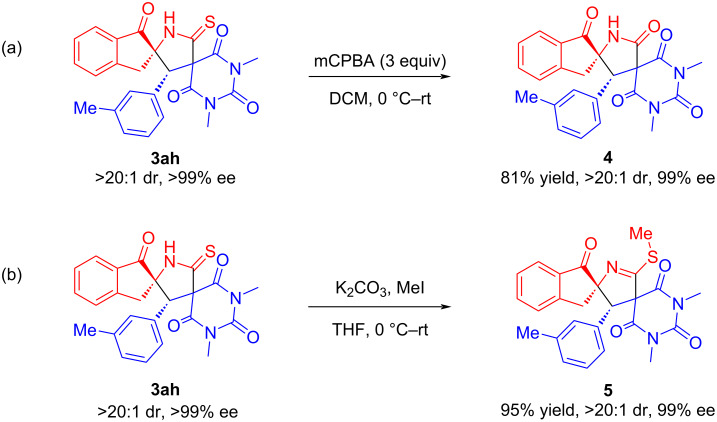
Further transformation of **3ah**.

Moreover, two different transformations of the product **3ah** are shown to validate synthetic utility of the reaction. As demonstrated in Scheme 5, the dispiro[indene-pyrrolidine-pyrimidine] **3ah** could be easily oxidized to compound **4** with *m*-chloroperbenzoic acid under mild conditions, and compound **4** can basically maintain the original excellent stereoselectivity (Scheme 5a). Meanwhile, we are pleased that methylation of **3ah** took place easily to afford product **5** in 95% chemical yield with 99% ee and >20:1 dr under the basic reaction conditions (Scheme 5b).

**Scheme 6:**
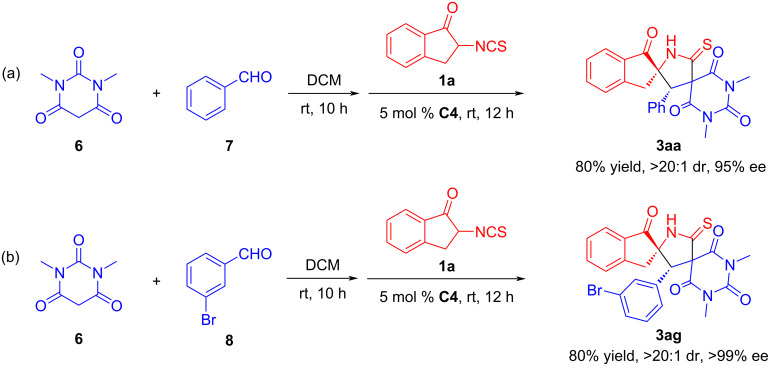
One-pot three-component reaction.

A one-pot reaction of three available starting materials was tested using CH_2_Cl_2_ as the solvent. The one-pot reaction of 1,3-dimethylbarbituric acid (**6**), benzaldehyde (**7**), and 2-isothiocyanato-1-indanone (**1a**) proceeded smoothly to provide the desired product **3aa** in 80% yield with 95% ee and >20:1 dr (Scheme 6a). In addition, the one-pot reaction of 1,3-dimethylbarbituric acid (**6**), *m*-bromobenzaldehyde (**8**), and 2-isothiocyanato-1-indanone (**1a**) was also investigated, and the reaction yield (80%) was lower than before, but the stereoselectivity (>20:1 dr, >99% ee) could still be maintained (Scheme 6b). This one-pot three-component reaction would be more convenient for potential industrial applications.

**Scheme 7:**
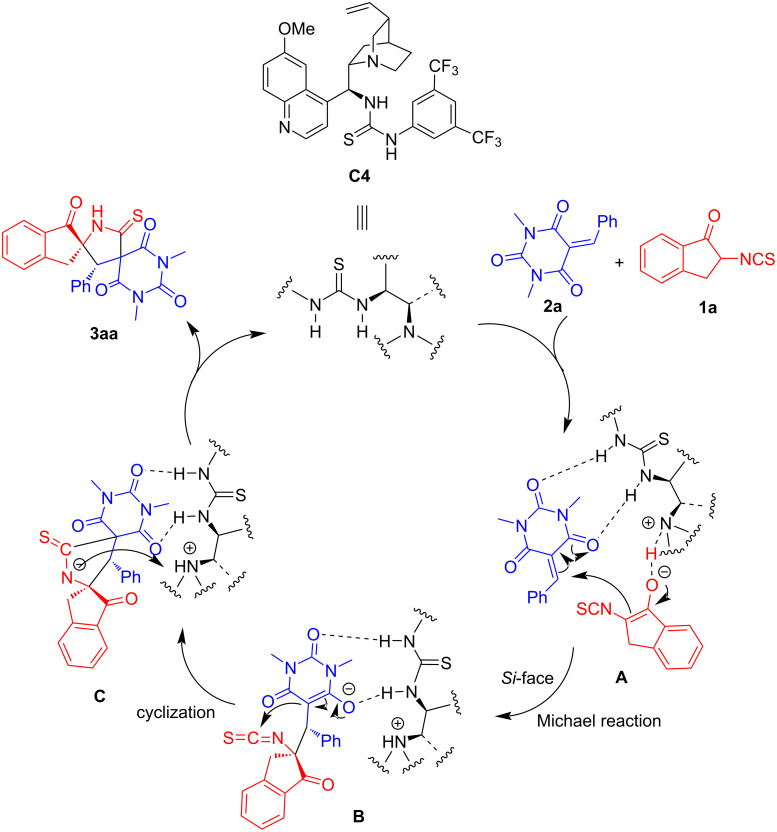
Proposed reaction mechanism.

Finally, in order to understand the enantioselective formation process of product **3**, we proposed the possible mechanisms for the [3 + 2] cyclization reaction of **1a** and **2a** based on the previously published work. As illustrated in Scheme 7, in this cycle, it is reasonable that the catalyst **C4** activates barbiturate-based olefins **2a** through the action of hydrogen bonds, and then the 2-isothiocyanato-1-indanone **1a** tautomerizes to form the corresponding enol under the action of catalyst **C4**. Simultaneously, deprotonated **1a** attacks the double bond of **2a** from the *Si* face via intermediate **A**, resulting in a Michael addition reaction. Then the electron-deficient isothiocyanate moiety is attacked by newly generated α-carbon center from barbiturate-based olefins **2a** to form intermediate **B**. Finally, the catalyst **C4** is removed in intermediate **C** and the product **3aa** is obtained.

## Conclusion

In summary, we have successfully developed an exceptionally efficient strategy for the enantioselective construction of indanone-derived spirobarbiturates through a simple organocatalytic domino Michael/cyclization reaction. This annulation reaction can be easily performed under air atmosphere and mild conditions with 5 mol % catalyst loading. By using bifunctional thiourea catalyst, a series of structurally diverse indanone-derived spirobarbiturates could be obtained in high yields and excellent diastereo- and enantioselectivities (up to >99% yield, >20:1 dr and >99% ee). In addition, a gram-scale synthesis, one-pot three-component reactions and further transformation experiments of the products were also demonstrated with excellent stereoselectivities. We believe that the availability of these compounds will provide promising candidates for chemical biology and drug discovery.

## Experimental

### General information

Commercially available compounds were used without further purification. Solvents were dried according to standard procedures. Column chromatography was performed with silica gel (200–300 mesh). Melting points were determined with an XT-4 melting-point apparatus and are uncorrected. ^1^H NMR spectra were measured with a Bruker Ascend 400 MHz spectrometer, chemical shifts are reported in δ (ppm) units relative to tetramethylsilane (TMS) as an internal standard. ^13^C NMR spectra were measured at 100 MHz with a 400 MHz spectrometer or at 176 MHz with a 700 MHz spectrometer, chemical shifts are reported in δ (ppm) units relative to tetramethylsilane and referenced to solvent peak (CDCl_3_, δ = 77.00 ppm; DMSO-*d*_6_, δ = 39.43 ppm). High-resolution mass spectra were measured with an Agilent 6520 Accurate-Mass Q-TOF MS system equipped with an electrospray ionization (ESI) source. Optical rotations were measured with a Krüss P8000 polarimeter at the indicated concentration with the units of g/100 mL. Enantiomeric excesses were determined by chiral HPLC analysis using an Agilent 1200 LC instrument with a Daicel Chiralpak IB, IC, or ADH column.

The following compounds were prepared following procedures reported in the literature: **1a**‒**g** [15], **2a**‒**o** [34], and chiral organocatalysts [35–38].

### 1. Procedure for the synthesis of racemates of **3**

To a dried small bottle were added **1** (0.06 mmol), **2** (0.05 mmol), Et_3_N (1.0 mg, 0.01 mmol, 0.2 equiv), and DCM (1.0 mL). The mixture was stirred at room temperature for 12 h, then the reaction mixture was concentrated and directly purified by silica gel column chromatography to afford the racemates of **3**.

### 2. Procedure for the synthesis of chiral compounds **3**

To a dried small bottle were added **1** (0.12 mmol), **2** (0.10 mmol), chiral organocatalyst **C4** (2.7 mg, 0.005 mmol, 5 mol %), and DCM (1.0 mL). The mixture was stirred at room temperature for 12‒40 h, then the reaction mixture was concentrated and directly purified by silica gel column chromatography to afford the desired products **3**.

### 3. Gram-scale synthesis of **3ah**

2-Isothiocyanato-2,3-dihydro-1*H*-inden-1-one (**1a**, 1.022 g, 5.4 mmol), 1,3-dimethyl-5-(3-methylbenzylidene)pyrimidine-2,4,6(1*H*,3*H*,5*H*)-trione (**2h**, 1.162 g, 4.5 mmol), and catalyst **C4** (122.4 mg, 5 mol %) were dissolved in dry DCM (45 mL) at room temperature. After stirring at room temperature for 39 h, the reaction mixture was concentrated, and directly purified by silica gel column chromatography (dichloromethane/ethyl acetate/petroleum ether 1:1:5) to afford the desired product **3ah** as white solid (1.976 g, 94% yield) with >20:1 dr and >99% ee.

### 4. Synthetic procedure for compound **4**

The synthesis of compound **4** was similar to the reported method in the literature [39]. In a 5 mL small bottle, compound **3ah** (44.8 mg, 0.10 mmol, 1.0 equiv) was dissolved in CH_2_Cl_2_ (2 mL) and the bottle was placed in an ice-water bath. Then *m*-CPBA (≈85%, 60.9 mg, 0.30 mmol, 3.0 equiv) was added to the reaction mixture at 0 °C. After completion of the addition, the reaction mixture was slowly warmed to room temperature and allowed to stir overnight. The residue was purified by silica gel column chromatography (petroleum ether/ethyl acetate 2:1) to give pure compound **4** as a white solid (35.1 mg, 81% yield).

### 5. Synthetic procedure for compound **5**

The synthesis of compound **5** was similar to the reported method in the literature [39]. To an oven dried 5 mL small bottle were added compound **3ah** (44.8 mg, 0.10 mmol, 1.0 equiv), dry K_2_CO_3_ (21.0 mg, 0.23 mmol, 1.50 equiv), and THF (2 mL). The solution was cooled to 0 °C and iodomethane (12.5 μL, 0.20 mmol, 2.0 equiv) was added dropwise to the reaction mixture. After completion of the addition, the reaction mixture was gradually warmed to room temperature and allowed to stir overnight. The residue was purified by flash column chromatography on silica gel (petroleum ether/ethyl acetate 4:1) to give pure compound **5** as white solid (44.0 mg, 95% yield).

### 6. One-pot three-component reaction for the synthesis of **3aa**

1,3-Dimethylpyrimidine-2,4,6(1*H*,3*H*,5*H*)-trione (15.6 mg, 0.10 mmol) and benzaldehyde (10.6 mg, 0.10 mmol) were dissolved in anhydrous CH_2_Cl_2_ (1.0 mL) and stirred at room temperature for 10 h. Then, catalyst **C4** (2.7 mg, 5 mol %) and compound **1a** (22.7 mg, 0.12 mmol) were added. After stirring at room temperature for another 12 h, the reaction mixture was concentrated and directly purified by silica gel column chromatography (dichloromethane/ethyl acetate/petroleum ether 1:1:5) to afford the desired product **3aa** as white solid (35.5 mg, 80% yield) with >20:1 dr and 95% ee.

### 7. One-pot three-component reaction for the synthesis of **3ag**

1,3-Dimethylpyrimidine-2,4,6(1*H*,3*H*,5*H*)-trione (15.6 mg, 0.10 mmol) and *m*-bromobenzaldehyde (18.5 mg, 0.10 mmol) were dissolved in anhydrous CH_2_Cl_2_ (1.0 mL) and stirred at room temperature for 10 h. Then, catalyst **C4** (2.7 mg, 5 mol %) and compound **1a** (22.7 mg, 0.12 mmol) were added. After stirring at room temperature for another 12 h, the reaction mixture was concentrated and directly purified by silica gel column chromatography (dichloromethane/ethyl acetate/petroleum ether 1:1:5) to afford the desired product **3ag** as a white solid (41.0 mg, 80% yield) with >20:1 dr and >99% ee.

## Supporting Information

File 1 Characterization data, copies of NMR spectra, and HPLC chromatograms of products.

File 2 Crystallographic data of compound **3ae**.
