# Supplementary material for: Bifunctional thiourea-catalyzed asymmetric [3 + 2] annulation reactions of 2-isothiocyanato-1-indanones with barbiturate-based olefins
Source: Beilstein J Org Chem. 2022 Jan 4;18:25–36. doi: 10.3762/bjoc.18.3 (PMC8744461; doi:10.3762/bjoc.18.3)
Supplement: File 1 — Characterization data, copies of NMR spectra, and HPLC chromatograms of products. [file Beilstein_J_Org_Chem-18-25-s001.pdf]

BEILSTEIN JOURNAL OF ORGANIC CHEMISTRY

Supporting Information

for

Bifunctional thiourea-catalyzed asymmetric [3 + 2] annulation reactions of 2-isothiocyanato-1-indanones with barbiturate-based olefins

Jiang-Song Zhai and Da-Ming Du

Beilstein J. Org. Chem. **2022,** *18,* 25–36. doi:10.3762/bjoc.18.3

Characterization data, copies of NMR spectra, and HPLC chromatograms of products

License and Terms: This is a supporting information file under the terms of the Creative Commons Attribution License (https://creativecommons.org/licenses/by/4.0). Please note that the reuse, redistribution and reproduction in particular requires that the author(s) and source are credited and that individual graphics may be subject to special legal provisions.
The license is subject to the *Beilstein Journal of Organic Chemistry* terms and conditions: (https://www.beilstein-journals.org/bjoc/terms)

Table of contents

1. Characterization data of compounds **3** S1
2. Characterization data of compound **4** S11
3. Characterization data of compound **5** S12
4. Copies of ^{1}H and ^{13}C NMR spectra of new compounds S13
5. Copies of HPLC chromatograms S59

1. Characterization data of compounds 3

(2*S*,3'*S*)-1'',3''-Dimethyl-3'-phenyl-5'-thioxo-2''*H*-dispiro[indene-2,2'-pyrrolidine-4',5''-pyrimidine]-1,2'',4'',6''(1''*H*,3*H*,3''*H*)-tetraone (3aa). From **1a** (22.7 mg, 0.12 mmol) and **2a** (24.4 mg, 0.10 mmol), purified by silica gel (200–300 mesh) column chromatography using dichloromethane/ethyl acetate/petroleum ether 1:1:5 as eluent to obtain 35.1 mg (81% yield) of compound **3aa** as a white solid; m.p. 252–254 °C. HPLC (Daicel Chiralpak IB, *n*-hexane/2-propanol 90:10, flow rate 1.0 mL/min, detection at 254 nm): t_R = 15.4 min (major), t_R = 20.3 min (minor); 97% ee. $[\alpha]_D^{25}$ = +51.5° (*c* = 1.60, THF). ^{1}H NMR (400 MHz, $CDCl_3$): δ 8.39 (s, 1H, NH), 7.85 (d, *J* = 7.6 Hz, 1H, ArH), 7.64 (t, *J* = 7.2 Hz, 1H, ArH), 7.41 (t, *J* = 7.6 Hz, 2H, ArH), 7.19–7.12 (m, 3H, ArH), 6.82 (d, *J* = 7.2 Hz, 2H, ArH), 5.51 (s, 1H, CH), 4.02 (d, *J* = 18.8 Hz, 1H, CH_2), 3.57 (d, *J* = 18.8 Hz, 1H, CH_2), 3.38 (s, 3H, CH_3), 3.36 (s, 3H, CH_3) ppm. ^{13}C NMR (100 MHz, $CDCl_3$): δ 200.7, 195.7, 165.55, 165.49, 152.4, 150.6, 136.9, 133.8, 132.0, 129.1, 128.3, 128.24, 128.20, 126.6, 125.2, 75.4, 73.0, 55.1, 37.8, 29.7, 29.2 ppm. HRMS (ESI): *m*/*z* calcd. for $C_{23}H_{20}N_3O_4S$ $[M + H]^+$ 434.1169, found 434.1169.

(2*S*,3'*S*)-1'',3'',6-Trimethyl-3'-phenyl-5'-thioxo-2''*H*-dispiro[indene-2,2'-pyrrolidine-4',5''-pyrimidine]-1,2'',4'',6''(1''*H*,3*H*,3''*H*)-tetraone (3ba). From **1b** (24.4 mg, 0.12 mmol) and **2a** (24.4 mg, 0.10 mmol), purified by silica gel (200–300 mesh) column chromatography using dichloromethane/ethyl acetate/petroleum ether 1:1:5 as eluent to obtain 39.9 mg (89% yield) of compound **3ba** as a white solid; m.p. 236–238 °C. HPLC (Daicel Chiralpak ADH,

n-hexane/2-propanol 70:30, flow rate 1.0 mL/min, detection at 254 nm): t_R = 9.3 min (minor), t_R = 14.2 min (major); 88% ee. $[\alpha]_D^{25}$ = +58.7° (*c* = 0.98, THF). ^{1}H NMR (400 MHz, $CDCl_3$): δ 8.42 (s, 1H, NH), 7.65 (s, 1H, ArH), 7.45 (dd, J_1 =7.8 Hz, J_2 = 1.0 Hz, 1H, ArH), 7.28 (d, *J* = 8.0 Hz, 1H, ArH), 7.19–7.11 (m, 3H, ArH), 6.81–6.79 (m, 2H, ArH), 5.50 (s, 1H, CH), 3.96 (d, *J* = 18.4 Hz, 1H, CH_2), 3.50 (d, *J* = 18.8 Hz, 1H, CH_2), 3.37 (s, 3H, CH_3), 3.36 (s, 3H, CH_3), 2.40 (s, 3H, CH_3) ppm. ^{13}C NMR (100 MHz, $CDCl_3$): δ 200.8, 195.6, 165.6, 165.5, 150.6, 149.9, 138.5, 138.2, 134.0, 132.1, 129.0, 128.15, 128.09, 126.3, 125.0, 75.7, 73.1, 55.0, 37.5, 29.7, 29.2, 21.1 ppm. HRMS (ESI): *m*/*z* calcd. for $C_{24}H_{22}N_3O_4S$ $[M + H]^+$ 448.1326, found 448.1314.

(2*S*,3'*S*)-6-Methoxy-1'',3''-dimethyl-3'-phenyl-5'-thioxo-2''*H*-dispiro[indene-2,2'-pyrrolidi-ne-4',5''-pyrimidine]-1,2'',4'',6''(1''*H*,3*H*,3''*H*)-tetraone (3ca). From **1c** (26.3 mg, 0.12 mmol) and **2a** (24.4 mg, 0.10 mmol), purified by silica gel (200–300 mesh) column chromatography using dichloromethane/ethyl acetate/petroleum ether 1:1:5 as eluent to obtain 23.1 mg (50% yield) compound **3ca** as a white solid; m.p. 242–244 °C. HPLC (Daicel Chiralpak ADH, *n*-hexane/2-propanol 70:30, flow rate 1.0 mL/min, detection at 254 nm): t_R = 12.1 min (minor), t_R = 26.3 min (major); 56% ee. $[\alpha]_D^{25}$ = +37.5° (*c* = 0.73, THF). ^{1}H NMR (400 MHz, $CDCl_3$): δ 8.19 (s, 1H, NH), 7.29–7.26 (m, 2H, ArH), 7.23 (dd, J_1 =8.2 Hz, J_2 = 2.6 Hz, 1H, ArH), 7.18–7.12 (m, 3H, ArH), 6.82–6.80 (m, 2H, ArH), 5.52 (s, 1H, CH), 3.95 (d, *J* = 18.4 Hz, 1H, CH_2), 3.85 (s, 3H, OCH_3), 3.47 (d, *J* = 18.8 Hz, 1H, CH_2), 3.39 (s, 3H, CH_3), 3.38 (s, 3H, CH_3) ppm. ^{13}C NMR (100 MHz, $CDCl_3$): δ 200.7, 195.7, 165.6, 165.4, 160.0, 150.6, 145.5, 134.9, 132.1, 129.1, 128.2, 128.1, 127.4, 126.6, 105.9, 76.0, 73.0, 55.7, 55.1, 37.3, 29.7, 29.3 ppm. HRMS (ESI): *m*/*z* calcd. for $C_{24}H_{22}N_3O_5S$ $[M + H]^+$ 464.1275, found 464.1266.

(2*S*,3'*S*)-5-Methoxy-1'',3''-dimethyl-3'-phenyl-5'-thioxo-2''*H*-dispiro[indene-2,2'-pyrrolidi-ne-4',5''-pyrimidine]-1,2'',4'',6''(1''*H*,3*H*,3''*H*)-tetraone (3da). From **1d** (26.3 mg, 0.12 mmol) and **2a** (24.4 mg, 0.10 mmol), purified by silica gel (200–300 mesh) column chromatography using dichloromethane/ethyl acetate/petroleum ether 1:1:5 as eluent to obtain 37.2 mg (80% yield) compound **3da** as a white solid, m.p. 225–227 °C. HPLC (Daicel Chiralpak ADH, *n*-hexane/2-propanol 70:30, flow rate 1.0 mL/min, detection at 254 nm): t_R = 12.0 min (minor), t_R = 26.1 min (major); 82% ee. $[\alpha]_D^{25}$ = +58.5° (*c* = 0.90, THF). ^{1}H NMR (400 MHz, $CDCl_3$): δ 8.49 (s, 1H, NH), 7.29–7.25 (m, 2H, ArH), 7.22 (dd, J_1 =8.2 Hz, J_2 = 2.6 Hz, 1H, ArH), 7.17–7.12

(m, 3H, ArH), 6.80 (d, *J* = 7.2 Hz, 2H, ArH), 5.49 (s, 1H, CH), 3.94 (d, *J* = 18.4 Hz, 1H, CH_2), 3.85 (s, 3H, OCH_3), 3.47 (d, *J* = 18.8 Hz, 1H, CH_2), 3.38 (s, 3H, CH_3), 3.36 (s, 3H, CH_3) ppm. ^{13}C NMR (100 MHz, $CDCl_3$): δ 200.9, 195.6, 165.6, 165.4, 159.9, 150.6, 145.5, 134.9, 132.0, 129.1, 128.2, 128.0, 127.4, 126.6, 105.9, 76.0, 73.0, 55.7, 55.2, 37.2, 29.7, 29.3 ppm. HRMS (ESI): *m*/*z* calcd. for $C_{24}H_{22}N_3O_5S$ $[M + H]^+$ 464.1275, found 464.1260.

(2*S*,3'*S*)-5-Fluoro-1'',3''-dimethyl-3'-phenyl-5'-thioxo-2''*H*-dispiro[indene-2,2'-pyrrolidine-4',5''-pyrimidine]-1,2'',4'',6''(1''*H*,3*H*,3''*H*)-tetraone (3ea). From **1e** (24.9 mg, 0.12 mmol) and **2a** (24.4 mg, 0.10 mmol), purified by silica gel (200–300 mesh) column chromatography using dichloromethane/ethyl acetate/petroleum ether 1:1:5 as eluent to obtain 36.2 mg (80% yield) compound **3ea** as a white solid, m.p. 236–238 °C. HPLC (Daicel Chiralpak ADH, *n*-hexane/2-propanol 70:30, flow rate 1.0 mL/min, detection at 254 nm): t_R = 13.6 min (major), t_R = 17.7 min (minor); 84% ee. $[\alpha]_D^{25}$ = +59.6° (*c* = 0.90, THF). ^1H NMR (400 MHz, $CDCl_3$): δ 8.61 (s, 1H, NH), 7.81 (dd, J_1 =8.4 Hz, J_2 = 5.2 Hz, 1H, ArH), 7.21–7.05 (m, 5H, ArH), 6.81 (d, *J* = 7.2 Hz, 2H, ArH), 5.45 (s, 1H, CH), 4.02 (d, *J* = 19.2 Hz, 1H, CH_2), 3.57 (d, *J* = 19.2 Hz, 1H, CH_2), 3.37 (s, 3H, CH_3), 3.36 (s, 3H, CH_3) ppm. ^{13}C NMR (100 MHz, $CDCl_3$): δ 199.1, 195.7, 168.4 (d, $^1J_{C–F}$ = 258.7 Hz), 165.5, 155.5 (d, $^3J_{C–F}$ = 10.5 Hz), 150.5, 131.7, 130.3 (d, $^4J_{C–F}$ = 1.3 Hz), 129.1, 128.4, 128.1, 127.8 (d, $^3J_{C–F}$ = 10.8 Hz), 116.9 (d, $^2J_{C–F}$ = 23.6 Hz), 113.4 (d, $^2J_{C–F}$ = 22.4 Hz), 75.6, 73.0, 55.3, 37.7, 29.7, 29.3 ppm. HRMS (ESI): *m*/*z* calcd. for $C_{23}H_{19}FN_3O_4S$ $[M + H]^+$ 452.1075, found 452.1069.

(2*S*,3'*S*)-5-Bromo-1'',3''-dimethyl-3'-phenyl-5'-thioxo-2''*H*-dispiro[indene-2,2'-pyrrolidine-4',5''-pyrimidine]-1,2'',4'',6''(1''*H*,3H,3''*H*)-tetraone (3fa). From **1f** (32.2 mg, 0.12 mmol) and **2a** (24.4 mg, 0.10 mmol), purified by silica gel (200–300 mesh) column chromatography using dichloromethane/ethyl acetate/petroleum ether 1:1:5 as eluent to obtain 35.0 mg (68% yield) compound **3fa** as a white solid, m.p. 235–237 °C. HPLC (Daicel Chiralpak ADH, *n*-hexane/2-propanol 70:30, flow rate 1.0 mL/min, detection at 254 nm): t_R = 14.3 min (major), t_R = 18.8 min (minor); 56% ee. $[\alpha]_D^{25}$ = +29.9° (*c* = 0.95, THF). ^1H NMR (400 MHz, $CDCl_3$): δ 8.76 (s, 1H, NH), 7.70 (d, *J* = 8.0 Hz, 1H, ArH), 7.57 (s, 1H, ArH), 7.54 (d, *J* = 8.0 Hz, 1H, ArH), 7.22–7.13 (m, 3H, ArH), 6.80 (d, *J* = 7.2 Hz, 2H, ArH), 5.45 (s, 1H, CH), 4.01 (d, *J* = 19.2 Hz, 1H, CH_2), 3.55 (d, *J* = 18.8 Hz, 1H, CH_2), 3.37 (s, 3H, CH_3), 3.36 (s, 3H, CH_3) ppm. ^{13}C NMR

(100 MHz, $CDCl_3$): δ 200.0, 195.8, 165.51, 165.49, 153.9, 150.5, 132.7, 132.6, 132.1, 131.7, 129.9, 129.2, 128.4, 128.1, 126.3, 75.3, 73.0, 55.3, 37.5, 29.7, 29.3 ppm. HRMS (ESI): *m*/*z* calcd. for $C_{23}H_{19}{}^{79}BrN_3O_4S$ $[M + H]^+$ 512.0274, found 512.0270; calcd. for $C_{23}H_{19}{}^{81}BrN_3O_4S$ $[M + H]^+$ 514.0254, found 514.0252.

(2S,3'S)-5,6-Dimethoxy-1'',3''-dimethyl-3'-phenyl-5'-thioxo-2''*H*-dispiro[indene-2,2'-pyrrolidine-4',5''-pyrimidine]-1,2'',4'',6''(1''*H*,3*H*,3''*H*)-tetraone (3ga). From **1g** (29.9 mg, 0.12 mmol) and **2a** (24.4 mg, 0.10 mmol), purified by silica gel (200–300 mesh) column chromatography using dichloromethane/ethyl acetate/petroleum ether 1:1:2 as eluent to obtain 25.2 mg (51% yield) compound **3ga** as a white solid, m.p. 261–263 °C. HPLC (Daicel Chiralpak ADH, *n*-hexane/2-propanol 70:30, flow rate 1.0 mL/min, detection at 254 nm): t_R = 23.8 min (minor), t_R = 47.2 min (major); 47% ee. $[\alpha]_D^{25}$ = +33.9° (*c* = 0.63, THF). ^1H NMR (400 MHz, $CDCl_3$): δ 8.54 (d, *J* = 4.0 Hz, 1H, NH), 7.21 (s, 1H, ArH), 7.19–7.13 (m, 3H, ArH), 6.80–6.78 (m, 2H, ArH), 6.76 (s, 1H, ArH), 5.53 (s, 1H, CH), 3.93 (s, 3H, OCH_3), 3.91 (s, 3H, OCH_3), 3.89 (d, *J* = 19.2 Hz, 1H, CH_2), 3.43 (d, *J* = 19.2 Hz, 1H, CH_2), 3.40 (s, 3H, CH_3), 3.38 (s, 3H, CH_3) ppm. ^{13}C NMR (176 MHz, $CDCl_3$): δ 198.7, 195.5, 165.7, 165.5, 157.2, 150.6, 150.0, 148.5, 132.3, 129.1, 128.1, 127.9, 126.5, 107.2, 105.1, 75.7, 73.0, 56.4, 56.2, 54.7, 37.7, 29.7, 29.3 ppm. HRMS (ESI): *m*/*z* calcd. for $C_{25}H_{24}N_3O_6S$ $[M + H]^+$ 494.1380, found 494.1381.

(2*S*,3'*S*)-3'-(4-Fluorophenyl)-1'',3''-dimethyl-5'-thioxo-2''*H*-dispiro[indene-2,2'-pyrrolidine-4',5''-pyrimidine]-1,2'',4'',6''(1''*H*,3*H*,3''*H*)-tetraone (3ab). From **1a** (22.7 mg, 0.12 mmol) and **2a** (26.2 mg, 0.10 mmol), purified by silica gel (200–300 mesh) column chromatography using dichloromethane/ethyl acetate/petroleum ether 1:1:5 as eluent to obtain 43.9 mg (97% yield) compound **3ab** as a white solid, m.p. 241–243 °C. HPLC (Daicel Chiralpak IC, *n*-hexane/2-propanol 70:30, flow rate 1.0 mL/min, detection at 254 nm): t_R = 13.5 min (major), t_R = 22.6 min (minor); 95% ee. $[\alpha]_D^{25}$ = +67.2° (*c* = 0.95, THF). ^1H NMR (400 MHz, DMSO): δ 11.57 (s, 1H, NH), 7.87 (d, *J* = 7.6 Hz, 1H, ArH), 7.64 (td, J_1 = 7.6 Hz, J_2 = 0.8 Hz, 1H, ArH), 7.60 (d, *J* = 7.6 Hz, 1H, ArH), 7.54 (t, *J* = 7.4 Hz, 1H, ArH), 7.03–6.98 (m, 2H, ArH), 6.77–6.73 (m, 2H, ArH), 5.30 (s, 1H, CH), 3.84 (d, *J* = 19.6 Hz, 1H, CH_2), 3.37 (d, *J* = 19.6 Hz, 1H, CH_2), 3.29 (s, 3H, CH_3), 3.27 (s, 3H, CH_3) ppm. ^{13}C NMR (100 MHz, DMSO): δ 201.9, 193.3, 165.2, 164.4, 161.2 (d, $^1J_{C–F}$ = 243.9 Hz), 152.6, 150.3, 137.1, 133.6, 129.4 (d, $^3J_{C–F}$ = 8.1 Hz), 128.42 (d,

$^4J_{C–F}$ = 3.3 Hz), 128.38, 126.9, 124.4, 115.8 (d, $^2J_{C–F}$ = 21.3 Hz), 74.5, 73.1, 52.8, 37.6, 29.3, 28.9 ppm. HRMS (ESI): *m*/*z* calcd. for $C_{23}H_{19}FN_3O_4S$ [M + H]$^+$ 452.1075, found 452.1069.

(2*S*,3'*S*)-3'-(4-Chlorophenyl)-1'',3''-dimethyl-5'-thioxo-2''*H*-dispiro[indene-2,2'-pyrrolidine-4',5''-pyrimidine]-1,2'',4'',6''(1''*H*,3*H*,3''*H*)-tetraone (3ac). From **1a** (22.7 mg, 0.12 mmol) and **2c** (27.9 mg, 0.10 mmol), purified by silica gel (200–300 mesh) column chromatography using dichloromethane/ethyl acetate/petroleum ether 1:1:5 as eluent to obtain 43.0 mg (92% yield) compound **3ac** as a white solid, m.p. 237–239 °C. HPLC (Daicel Chiralpak IC, *n*-hexane/2-propanol = 85:15, flow rate 1.0 mL/min, detection at 254 nm): t_R = 30.9 min (major), t_R = 37.9 min (minor); 96% ee. $[\alpha]_D^{25}$ = +84.1° (*c* = 2.35, THF). ^{1}H NMR (400 MHz, $CDCl_3$): δ 8.30 (s, 1H, NH), 7.84 (d, *J* = 7.6 Hz, 1H, ArH), 7.67–7.63 (m, 1H, ArH), 7.42 (t, *J* = 7.4 Hz, 2H, ArH), 7.12 (d, *J* = 8.4 Hz, 2H, ArH), 6.84 (d, *J* = 8.4 Hz, 2H, ArH), 5.48 (s, 1H, CH), 4.00 (d, *J* = 18.8 Hz, 1H, CH_2), 3.58 (d, *J* = 19.2 Hz, 1H, CH_2), 3.374 (s, 3H, CH_3), 3.365 (s, 3H, CH_3) ppm. ^{13}C NMR (100 MHz, $CDCl_3$): δ 200.5, 195.4, 165.26, 165.25, 152.2, 150.4, 137.1, 134.5, 133.7, 130.4, 130.0, 129.3, 128.5, 126.7, 125.2, 75.2, 73.2, 54.2, 37.8, 29.8, 29.3 ppm. HRMS (ESI): *m*/*z* calcd. for $C_{23}H_{19}ClN_3O_4S$ [M + H]$^+$ 468.0779, found 468.0781.

(2*S*,3'*S*)-3'-(4-Bromophenyl)-1'',3''-dimethyl-5'-thioxo-2''*H*-dispiro[indene-2,2'-pyrrolidine-4',5''-pyrimidine]-1,2'',4'',6''(1''*H*,3*H*,3''*H*)-tetraone (3ad). From **1a** (22.7 mg, 0.12 mmol) and **2d** (32.3 mg, 0.10 mmol), purified by silica gel (200–300 mesh) column chromatography using dichloromethane/ethyl acetate/petroleum ether 1:1:5 as eluent to obtain 46.3 mg (90% yield) compound **3ad** as a white solid, m.p. 236–238 °C. HPLC (Daicel Chiralpak IC, *n*-hexane/2-propanol 70:30, flow rate 1.0 mL/min, detection at 254 nm): t_R = 11.8 min (minor), t_R = 13.9 min (major); 84% ee. $[\alpha]_D^{25}$ = +53.5° (*c* = 0.65, THF). ^{1}H NMR (400 MHz, $CDCl_3$): δ 8.23 (s, 1H, NH), 7.84 (d, *J* = 7.6 Hz, 1H, ArH), 7.68–7.64 (m, 1H, ArH), 7.42 (t, *J* = 7.6 Hz, 2H, ArH), 7.28 (d, *J* = 8.8 Hz, 2H, ArH), 6.78 (d, *J* = 8.4 Hz, 2H, ArH), 5.48 (s, 1H, CH), 4.00 (d, *J* = 18.8 Hz, 1H, CH_2), 3.57 (d, *J* = 18.8 Hz, 1H, CH_2), 3.38 (s, 3H, CH_3), 3.37 (s, 3H, CH_3) ppm. ^{13}C NMR (100 MHz, $CDCl_3$): δ 200.5, 195.3, 165.23, 165.22, 152.1, 150.4, 137.1, 133.7, 132.3, 131.0, 130.2, 128.5, 126.7, 125.3, 122.7, 75.1, 73.1, 54.1, 37.8, 29.8, 29.3 ppm. HRMS (ESI): *m*/*z* calcd. for $C_{23}H_{19}{}^{79}BrN_3O_4S$ [M + H]$^+$ 512.0274, found 512.0290; calcd. for $C_{23}H_{19}{}^{81}BrN_3O_4S$ [M + H]$^+$ 514.0254, found 514.0271.

(2*S*,3'*S*)-1'',3''-Dimethyl-3'-(4-nitrophenyl)-5'-thioxo-2''*H*-dispiro[indene-2,2'-pyrrolidine-4',5''-pyrimidine]-1,2'',4'',6''(1''*H*,3*H*,3''*H*)-tetraone (3ae). From **1a** (22.7 mg, 0.12 mmol) and **2e** (28.9 mg, 0.10 mmol), purified by silica gel (200–300 mesh) column chromatography using dichloromethane/ethyl acetate/petroleum ether 1:1:5 as eluent to obtain 22.9 mg (48% yield) compound **3ae** as a white solid, m.p. 240–242 °C. HPLC (Daicel Chiralpak ADH, *n*-hexane/2-propanol = 85:15, flow rate 1.0 mL/min, detection at 254 nm): t_R = 37.4 min (minor), t_R = 47.3 min (major); 89% ee. $[\alpha]_D^{25}$ = +38.4° (*c* = 1.15, THF). ^{1}H NMR (400 MHz, DMSO): δ 11.68 (s, 1H, NH), 8.02 (d, *J* = 8.8 Hz, 2H, ArH), 7.93 (d, *J* = 7.6 Hz, 1H, ArH), 7.83 (td, J_1 = 7.6 Hz, J_2 = 0.8 Hz, 1H, ArH), 7.64 (d, *J* = 7.6 Hz, 1H, ArH), 7.58 (t, *J* = 7.4 Hz, 1H, ArH), 6.96 (d, *J* = 8.4 Hz, 2H, ArH), 5.48 (s, 1H, CH), 3.79 (d, *J* = 19.2 Hz, 1H, CH_2), 3.39 (d, *J* = 19.6 Hz, 1H, CH_2), 3.33 (s, 3H, CH_3), 3.28 (s, 3H, CH_3) ppm. ^{13}C NMR (100 MHz, DMSO): δ 201.5, 192.5, 165.0, 164.1, 152.6, 150.4, 146.5, 140.0, 137.2, 133.5, 128.6, 127.0, 124.7, 124.0, 74.2, 72.9, 52.3, 37.8, 29.4, 29.0 ppm. HRMS (ESI): *m*/*z* calcd. for $C_{23}H_{19}N_4O_6S$ $[M + H]^+$ 479.1020, found 479.1026.

(2*S*,3'*S*)-3'-(4-Methoxyphenyl)-1'',3''-dimethyl-5'-thioxo-2''*H*-dispiro[indene-2,2'-pyrrolidine-4',5''-pyrimidine]-1,2'',4'',6''(1''*H*,3*H*,3''*H*)-tetraone (3af). From **1a** (22.7 mg, 0.12 mmol) and **2f** (27.4 mg, 0.10 mmol), purified by silica gel (200–300 mesh) column chromatography using dichloromethane/ethyl acetate/petroleum ether 1:1:5 as eluent to obtain 45.8 mg (99% yield) compound **3af** as a white solid, m.p. 239–241 °C. HPLC (Daicel Chiralpak IB, *n*-hexane/2-propanol 70:30, flow rate 1.0 mL/min, detection at 254 nm): t_R = 7.8 min (major), t_R = 9.7 min (minor); 96% ee. $[\alpha]_D^{25}$ = +72.3° (*c* = 0.68, THF). ^{1}H NMR (400 MHz, $CDCl_3$): δ 8.58 (s, 1H, NH), 7.80 (d, *J* = 8.0 Hz, 1H, ArH), 7.61 (t, *J* = 7.2 Hz, 1H, ArH), 7.39–7.35 (m, 2H, ArH), 6.86 (d, *J* = 8.8 Hz, 2H, ArH), 6.65 (d, *J* = 8.8 Hz, 2H, ArH), 5.36 (s, 1H, CH), 4.03 (d, *J* = 18.8 Hz, 1H, CH_2), 3.67 (s, 3H, OCH_3), 3.59 (d, *J* = 18.8 Hz, 1H, CH_2), 3.34 (s, 3H, CH_3), 3.33 (s, 3H, CH_3) ppm. ^{13}C NMR (100 MHz, $CDCl_3$): δ 200.8, 196.1, 165.77, 165.76, 159.5, 152.3, 150.5, 136.8, 133.8, 130.0, 128.2, 126.6, 125.0, 123.3, 114.4, 75.7, 73.4, 55.4, 55.1, 37.5, 29.6, 29.2 ppm. HRMS (ESI): *m*/*z* calcd. for $C_{24}H_{22}N_3O_5S$ $[M + H]^+$ 464.1275, found 464.1274.

(2*S*,3'*S*)-3'-(3-Bromophenyl)-1'',3''-dimethyl-5'-thioxo-2''*H*-dispiro[indene-2,2'-pyrrolidine-4',5''-pyrimidine]-1,2'',4'',6''(1''*H*,3*H*,3''*H*)-tetraone (3ag). From **1a** (22.7 mg, 0.12 mmol) and **2g** (32.3 mg, 0.10 mmol), purified by silica gel (200–300 mesh) column chromatography using dichloromethane/ethyl acetate/petroleum ether 1:1:5 as eluent to obtain 47.1 mg (92% yield) compound **3ag** as a white solid, m.p. 236–238 °C. HPLC (Daicel Chiralpak IC, *n*-hexane/2-propanol 70:30, flow rate 1.0 mL/min, detection at 254 nm): t_R = 11.6 min (major), t_R = 20.7 min (minor); 99% ee. $[\alpha]_D^{25}$ = +56.7° (*c* = 1.23, THF). ^{1}H NMR (400 MHz, $CDCl_3$): δ 8.53 (s, 1H, NH), 7.84 (d, *J* = 7.6 Hz, 1H, ArH), 7.67–7.63 (m, 1H, ArH), 7.41 (t, *J* = 7.0 Hz, 2H, ArH), 7.31 (d, *J* = 8.0 Hz, 1H, ArH), 7.05–7.00 (m, 2H, ArH), 6.83 (d, *J* = 7.6 Hz, 1H, ArH), 5.43 (s, 1H, CH), 3.99 (d, *J* = 18.8 Hz, 1H, CH_2), 3.59 (d, *J* = 18.8 Hz, 1H, CH_2), 3.36 (s, 6H, CH_3) ppm. ^{13}C NMR (100 MHz, $CDCl_3$): δ 200.5, 195.2, 165.28, 165.26, 152.1, 150.4, 137.0, 134.1, 133.7, 131.61, 131.58, 130.5, 128.5, 127.4, 126.6, 125.2, 123.0, 75.3, 73.1, 54.4, 37.7, 29.7, 29.3 ppm. HRMS (ESI): *m*/*z* calcd. for $C_{23}H_{19}{}^{79}BrN_3O_4S$ [M + H]$^+$ 512.0274, found 512.0291; calcd. for $C_{23}H_{19}{}^{81}BrN_3O_4S$ [M + H]$^+$ 514.0254, found 514.0275.

(2*S*,3'*S*)-1'',3''-Dimethyl-5'-thioxo-3'-(*m*-tolyl)-2''*H*-dispiro[indene-2,2'-pyrrolidine-4',5''-pyrimidine]-1,2'',4'',6''(1''*H*,3*H*,3''*H*)-tetraone (3ah). From **1a** (22.7 mg, 0.12 mmol) and **2h** (25.8 mg, 0.10 mmol), purified by silica gel (200–300 mesh) column chromatography using dichloromethane/ethyl acetate/petroleum ether 1:1:5 as eluent to obtain 44.6 mg (>99% yield) compound **3ah** as a white solid, m.p. 235–237 °C. HPLC (Daicel Chiralpak IC, *n*-hexane/2-propanol 70:30, flow rate 1.0 mL/min, detection at 254 nm): t_R = 17.4 min (major), t_R = 57.8 min (minor); >99% ee. $[\alpha]_D^{25}$ = +63.1° (*c* = 3.87, THF). ^{1}H NMR (400 MHz, $CDCl_3$): δ 8.92 (s, 1H, NH), 7.83 (d, *J* = 8.0 Hz, 1H, ArH), 7.61 (t, *J* = 7.4 Hz, 1H, ArH), 7.38 (t, *J* = 7.4 Hz, 2H, ArH), 7.03 (t, *J* = 7.6 Hz, 1H, ArH), 6.97 (d, *J* = 7.6 Hz, 1H, ArH), 6.67 (d, *J* = 7.6 Hz, 1H, ArH), 6.61 (s, 1H, ArH), 5.38 (s, 1H, CH), 4.01 (d, *J* = 18.8 Hz, 1H, CH_2), 3.59 (d, *J* = 18.8 Hz, 1H, CH_2), 3.329 (s, 3H, CH_3), 3.326 (s, 3H, CH_3), 2.11 (s, 3H, CH_3) ppm. ^{13}C NMR (100 MHz, $CDCl_3$): δ 201.0, 195.8, 165.71, 165.66, 152.4, 150.5, 138.7, 136.8, 133.8, 131.5, 129.3, 129.1, 128.8, 128.2, 126.5, 125.12, 125.05, 75.6, 73.1, 55.7, 37.6, 29.6, 29.1, 21.3 ppm. HRMS (ESI): *m*/*z* calcd. for $C_{24}H_{22}N_3O_4S$ [M + H]$^+$ 448.1326, found 448.1325.

(2*S*,3'*S*)-3'-(3-Methoxyphenyl)-1'',3''-dimethyl-5'-thioxo-2''*H*-dispiro[indene-2,2'-pyrrolidine-4',5''-pyrimidine]-1,2'',4'',6''(1''*H*,3*H*,3''*H*)-tetraone (3ai). From **1a** (22.7 mg, 0.12 mmol) and **2i** (27.4 mg, 0.10 mmol), purified by silica gel (200–300 mesh) column chromatography using dichloromethane/ethyl acetate/petroleum ether 1:1:5 as eluent to obtain 46.2 mg (>99% yield) compound **3ai** as a white solid, m.p. 242–244 °C. HPLC (Daicel Chiralpak ADH, *n*-hexane/2-propanol 70:30, flow rate 1.0 mL/min, detection at 254 nm): t_R = 11.3 min (major), t_R = 17.6 min (minor); 93% ee. $[\alpha]_D^{25}$ = +54.3° (*c* = 1.23, THF). ^{1}H NMR (400 MHz, $CDCl_3$): δ 8.61 (s, 1H, NH), 7.85 (d, *J* = 7.2 Hz, 1H, ArH), 7.64 (td, J_1 = 7.6 Hz, J_2 = 0.8 Hz, 1H, ArH), 7.41 (t, *J* = 7.4 Hz, 2H, ArH), 7.05 (t, *J* = 8.0 Hz, 1H, ArH), 6.68 (dd, J_1 = 8.0 Hz, J_2 = 2.4 Hz, 1H, ArH), 6.35 (dt, J_1 = 7.6 Hz, J_2 = 0.8 Hz, 1H, ArH), 6.28–6.27 (m, 1H, ArH), 5.46 (s, 1H, CH), 4.03 (d, *J* = 19.2 Hz, 1H, CH_2), 3.56 (d, *J* = 19.2 Hz, 1H, CH_2), 3.49 (s, 3H, OCH_3), 3.37 (s, 3H, CH_3), 3.36 (s, 3H, CH_3) ppm. ^{13}C NMR (176 MHz, $CDCl_3$): δ 201.0, 195.6, 165.6, 165.4, 159.7, 152.6, 150.6, 136.9, 133.9, 133.4, 130.1, 128.3, 126.7, 125.1, 120.0, 113.8, 113.7, 75.3, 72.9, 55.2, 54.8, 37.9, 29.7, 29.2 ppm. HRMS (ESI): *m*/*z* calcd. for $C_{24}H_{22}N_3O_5S$ $[M + H]^+$ 464.1275, found 464.1267.

(2*S*,3'*S*)-3'-(3,4-Dimethoxyphenyl)-1'',3''-dimethyl-5'-thioxo-2''*H*-dispiro[indene-2,2'-pyrrolidine-4',5''-pyrimidine]-1,2'',4'',6''(1''*H*,3*H*,3''*H*)-tetraone (3aj). From **1a** (22.7 mg, 0.12 mmol) and **2j** (30.4 mg, 0.10 mmol), purified by silica gel (200–300 mesh) column chromatography using dichloromethane/ethyl acetate/petroleum ether 1:1:2 as eluent to obtain 47.8 mg (97% yield) compound **3aj** as a white solid, m.p. 201–203 °C. HPLC (Daicel Chiralpak IC, *n*-hexane/2-propanol 70:30, flow rate 1.0 mL/min, detection at 254 nm): t_R = 34.7 min (minor), t_R = 43.7 min (major); 94% ee. $[\alpha]_D^{25}$ = +57.8° (*c* = 1.18, THF). ^{1}H NMR (400 MHz, $CDCl_3$): δ 8.74 (s, 1H, NH), 7.78 (d, *J* = 7.6 Hz, 1H, ArH), 7.63–7.59 (m, 1H, ArH), 7.37 (t, *J* = 7.2 Hz, 2H, ArH), 6.61 (d, *J* = 8.4 Hz, 1H, ArH), 6.42 (dd, J_1 = 8.4 Hz, J_2 = 2.0 Hz, 1H, ArH), 6.26 (d, *J* = 2.0 Hz, 1H, ArH), 5.36 (s, 1H, CH), 4.04 (d, *J* = 18.8 Hz, 1H, CH_2), 3.73 (s, 3H, OCH_3), 3.55 (d, *J* = 19.2 Hz, 1H, CH_2), 3.43 (s, 3H, OCH_3), 3.37 (s, 3H, CH_3), 3.36 (s, 3H, CH_3) ppm. ^{13}C NMR (100 MHz, $CDCl_3$): δ 201.3, 196.0, 165.70, 165.66, 152.5, 150.5, 148.9, 148.8, 136.8, 133.9, 128.3, 126.6, 124.9, 123.9, 120.5, 111.6, 111.2, 75.4, 73.1, 55.70, 55.66, 55.3, 37.8, 29.7, 29.2 ppm. HRMS (ESI): *m*/*z* calcd. for $C_{25}H_{24}N_3O_6S$ $[M + H]^+$ 494.1380, found 494.1387.

(2*S*,3'*S*)-1'',3''-Dimethyl-5'-thioxo-3'-(*o*-tolyl)-2''*H*-dispiro[indene-2,2'-pyrrolidine-4',5''-pyrimidine]-1,2'',4'',6''(1''*H*,3*H*,3''*H*)-tetraone (3ak). From **1a** (22.7 mg, 0.12 mmol) and **2k** (25.8 mg, 0.10 mmol), purified by silica gel (200–300 mesh) column chromatography using dichloromethane/ethyl acetate/petroleum ether 1:1:5 as eluent to obtain 42.1 mg (94% yield) compound **3ak** as a white solid, m.p. 237–239 °C. HPLC (Daicel Chiralpak IB, *n*-hexane/2-propanol 70:30, flow rate 1.0 mL/min, detection at 254 nm): t_R = 8.9 min (major), t_R = 11.9 min (minor); 84% ee. $[\alpha]_D^{25}$ = +95.1° (*c* = 1.15, THF). ^{1}H NMR (400 MHz, DMSO): δ 11.52 (s, 1H, NH), 7.69 (d, *J* = 7.6 Hz, 1H, ArH), 7.66–7.62 (m, 1H, ArH), 7.46 (d, *J* = 7.6 Hz, 1H, ArH), 7.42 (d, *J* = 7.6 Hz, 1H, ArH), 7.38 (t, *J* = 7.4 Hz, 1H, ArH), 7.14–7.10 (m, 1H, ArH), 7.05 (td, J_1 =7.4 Hz, J_2 = 0.8 Hz, 1H, ArH), 6.99 (d, *J* = 7.2 Hz, 1H, ArH), 5.44 (s, 1H, CH), 4.13 (d, *J* = 18.8 Hz, 1H, CH_2), 3.47 (d, *J* = 18.8 Hz, 1H, CH_2), 3.26 (s, 3H, CH_3), 3.15 (s, 3H, CH_3), 2.09 (s, 3H, CH_3) ppm. ^{13}C NMR (100 MHz, DMSO): δ 202.4, 195.4, 166.3, 166.1, 152.0, 149.6, 137.9, 136.8, 133.2, 131.1, 128.9, 128.5, 128.23, 128.16, 126.5, 126.3, 123.8, 75.4, 74.2, 51.2, 36.2, 29.0, 28.8, 19.2 ppm. HRMS (ESI): *m*/*z* calcd. for $C_{24}H_{22}N_3O_4S$ $[M + H]^+$ 448.1326, found 448.1337.

(2*S*,3'*R*)-3'-(2-Bromophenyl)-1'',3''-dimethyl-5'-thioxo-2''*H*-dispiro[indene-2,2'-pyrrolidine-4',5''-pyrimidine]-1,2'',4'',6''(1''*H*,3*H*,3''*H*)-tetraone (3al). From **1a** (22.7 mg, 0.12 mmol) and **2l** (32.3 mg, 0.10 mmol), purified by silica gel (200–300 mesh) column chromatography using dichloromethane/ethyl acetate/petroleum ether 1:1:5 as eluent to obtain 22.0 mg (43% yield) compound **3al** as a white solid, m.p. 242–244 °C. HPLC (Daicel Chiralpak ADH, *n*-hexane/2-propanol 70:30, flow rate 1.0 mL/min, detection at 254 nm): t_R = 12.2 min (minor), t_R = 14.7 min (major); 76% ee. $[\alpha]_D^{25}$ = +91.0° (*c* = 0.90, THF). ^{1}H NMR (400 MHz, DMSO): δ 11.55 (s, 1H, NH), 7.68 (d, *J* = 7.6 Hz, 1H, ArH), 7.60–7.55 (m, 2H, ArH), 7.40–7.35 (m, 3H, ArH), 7.34–7.30 (m, 1H, ArH), 7.08 (td, J_1 = 7.6 Hz, J_2 = 1.2 Hz, 1H, ArH), 5.67 (s, 1H, CH), 4.06 (d, *J* = 18.4 Hz, 1H, CH_2), 3.38 (d, *J* = 18.4 Hz, 1H, CH_2), 3.25 (s, 3H, CH_3), 3.16 (s, 3H, CH_3) ppm. ^{13}C NMR (100 MHz, DMSO): δ 201.2, 195.1, 165.8, 165.6, 150.8, 149.6, 136.4, 133.6, 133.4, 130.5, 130.0, 129.8, 128.1, 127.9, 126.23, 126.21, 123.8, 75.9, 73.9, 53.9, 35.8, 29.1, 28.8 ppm. HRMS (ESI): *m*/*z* calcd. for $C_{23}H_{19}{}^{79}BrN_3O_4S$ $[M + H]^+$ 512.0274, found 512.0271; calcd. for $C_{23}H_{19}{}^{81}BrN_3O_4S$ $[M + H]^+$ 514.0254, found 514.0253.

(2*S*,3'*S*)-1'',3''-Dimethyl-3'-(naphthalen-1-yl)-5'-thioxo-2''*H*-dispiro[indene-2,2'-pyrrolidine-4',5''-pyrimidine]-1,2'',4'',6''(1''*H*,3*H*,3''*H*)-tetraone (3am). From **1a** (22.7 mg, 0.12 mmol) and **2m** (29.4 mg, 0.10 mmol), purified by silica gel (200–300 mesh) column chromatography using dichloromethane/ethyl acetate/petroleum ether (1:1:4) as eluent to obtain 33.1 mg (68% yield) compound **3am** as a white solid, m.p. 240–242 °C. HPLC (Daicel Chiralpak ADH, *n*-hexane/2-propanol = 85:15, flow rate 1.0 mL/min, detection at 254 nm): t_R = 26.9 min (minor), t_R = 30.9 min (major); 57% ee. $[\alpha]_D^{25}$ = +70.8° (*c* = 0.93, THF). ^{1}H NMR (400 MHz, DMSO): δ 11.63 (s, 1H, NH), 8.04 (d, *J* = 8.8 Hz, 1H, ArH), 7.76 (d, *J* = 8.0 Hz, 2H, ArH), 7.73 (d, *J* = 7.2 Hz, 1H, ArH), 7.56–7.51 (m, 2H, ArH), 7.49 (d, *J* = 7.6 Hz, 1H, ArH), 7.46–7.40 (m, 3H, ArH), 7.21 (t, *J* = 7.4 Hz, 1H, ArH), 6.15 (s, 1H, CH), 4.18 (d, *J* = 18.8 Hz, 1H, CH_2), 3.53 (d, *J* = 18.4 Hz, 1H, CH_2), 3.32 (s, 3H, CH_3), 3.02 (s, 3H, CH_3) ppm. ^{13}C NMR (100 MHz, DMSO): δ 202.1, 195.3, 166.1, 166.0, 151.9, 149.7, 136.6, 133.4, 133.2, 131.7, 129.4, 128.8, 127.9, 127.1, 126.9, 126.6, 126.4, 125.8, 125.0, 123.8, 121.8, 75.4, 74.5, 48.8, 36.6, 29.0, 28.9 ppm. HRMS (ESI): *m*/*z* calcd. for $C_{27}H_{22}N_3O_4S$ $[M + H]^+$ 484.1326, found 484.1337.

(2*S*,3'*S*)-3'-(Furan-2-yl)-1'',3''-dimethyl-5'-thioxo-2''*H*-dispiro[indene-2,2'-pyrrolidine-4',5''-pyrimidine]-1,2'',4'',6''(1''*H*,3*H*,3''*H*)-tetraone (3an). From **1a** (22.7 mg, 0.12 mmol) and **2n** (23.4 mg, 0.10 mmol), purified by silica gel (200–300 mesh) column chromatography using dichloromethane/ethyl acetate/petroleum ether (1:1:4) as eluent to obtain 38.9 mg (92% yield) compound **3an** as a white solid, m.p. 245–247 °C. HPLC (Daicel Chiralpak IC, *n*-hexane/2-propanol = 65:35, flow rate 1.0 mL/min, detection at 254 nm): t_R = 12.1 min (major), t_R = 26.7 min (minor); 97% ee. $[\alpha]_D^{25}$ = +65.6° (*c* = 1.08, THF). ^{1}H NMR (400 MHz, $CDCl_3$): δ 8.28 (s, 1H, NH), 7.86 (d, *J* = 7.2 Hz, 1H, ArH), 7.71–7.67 (m, 1H, ArH), 7.45 (t, *J* = 7.2 Hz, 2H, ArH), 7.14 (d, *J* = 1.6 Hz, 1H, ArH), 6.13 (dd, J_1 =3.2 Hz, J_2 = 2.0 Hz, 1H, ArH), 5.66 (d, *J* = 3.2 Hz, 1H, ArH), 5.38 (s, 1H, CH), 4.18 (d, *J* = 19.2 Hz, 1H, CH_2), 3.48 (d, *J* = 19.2 Hz, 1H, CH_2), 3.42 (s, 3H, CH_3), 3.36 (s, 3H, CH_3) ppm. ^{13}C NMR (100 MHz, $CDCl_3$): δ 200.8, 195.5, 165.5, 164.8, 152.3, 150.7, 146.0, 142.6, 137.0, 133.7, 128.4, 126.5, 125.2, 110.8, 108.7, 74.4, 71.9, 50.4, 38.0, 29.8, 29.3 ppm. HRMS (ESI): *m*/*z* calcd. for $C_{21}H_{18}N_3O_5S$ $[M + H]^+$ 424.0962, found 424.0969.

(2*S*,3'*S*)-1'',3''-Dimethyl-3'-(thiophen-2-yl)-5'-thioxo-2''*H*-dispiro[indene-2,2'-pyrrolidine-4',5''-pyrimidine]-1,2'',4'',6''(1''*H*,3*H*,3''*H*)-tetraone (3ao). From **1a** (22.7 mg, 0.12 mmol) and **2o** (25.0 mg, 0.10 mmol), purified by silica gel (200–300 mesh) column chromatography using dichloromethane/ethyl acetate/petroleum ether (1:1:4) as eluent to obtain 36.0 mg (82% yield) compound **3ao** as a white solid, m.p. 232–234 °C. HPLC (Daicel Chiralpak IB, *n*-hexane/2-propanol = 90:10, flow rate 1.0 mL/min, detection at 254 nm): t_R = 17.2 min (major), t_R = 22.4 min (minor); 92% ee. $[\alpha]_D^{25}$ = +80.8° (*c* = 0.55, THF). ^{1}H NMR (400 MHz, $CDCl_3$): δ 8.24 (s, 1H, NH), 7.79 (d, *J* = 7.6 Hz, 1H, ArH), 7.63–7.60 (m, 1H, ArH), 7.41–7.36 (m, 2H, ArH), 7.12 (dd, J_1 = 5.2 Hz, J_2 = 0.8 Hz, 1H, ArH), 6.78 (dd, J_1 = 5.2 Hz, J_2 = 3.6 Hz, 1H, ArH), 6.73 (d, *J* = 3.2 Hz, 1H, ArH), 5.70 (s, 1H, CH), 4.32 (d, *J* = 18.8 Hz, 1H, CH_2), 3.57 (d, *J* = 18.4 Hz, 1H, CH_2), 3.38 (s, 3H, CH_3), 3.36 (s, 3H, CH_3) ppm. ^{13}C NMR (176 MHz, $CDCl_3$): δ 200.7, 195.6, 165.24, 165.17, 152.2, 150.4, 136.8, 133.7, 132.1, 129.5, 128.3, 126.9, 126.5, 125.0, 75.4, 73.7, 52.0, 38.0, 29.7, 29.3 ppm. HRMS (ESI): *m/z* calcd. for $C_{21}H_{18}N_3O_4S_2$ $[M + H]^+$ 440.0733, found 440.0732.

2. Characterization data of compound 4

(2*S*,3'*S*)-1'',3''-Dimethyl-3'-(*m*-tolyl)-2''*H*-dispiro[indene-2,2'-pyrrolidine-4',5''-pyrimidine]-1,2'',4'',5',6''(1''*H*,3*H*,3''*H*)-pentaone (4). From **3ah** (44.8 mg, 0.10 mmol, 1.0 equiv.) and *m*-CPBA (~85%, 60.9 mg, 0.30 mmol, 3.0 equiv.), purified by silica gel (200–300 mesh) column chromatography using ethyl acetate/petroleum ether (1:2) as eluent to obtain 35.1 mg (81% yield) compound **4** as a white solid, m.p. 234–236 °C. HPLC (Daicel Chiralpak ADH, *n*-hexane/2-propanol 70:30, flow rate 1.0 mL/min, detection at 254 nm): t_R = 11.7 min (minor), t_R = 20.3 min (major); 99% ee. $[\alpha]_D^{25}$ = +59.7° (*c* = 0.70, THF). ^{1}H NMR (400 MHz, $CDCl_3$): δ 7.80 (d, *J* = 7.6 Hz, 1H, ArH), 7.58 (t, *J* = 7.4 Hz, 1H, ArH), 7.38–7.34 (m, 2H, ArH), 7.01 (t, *J* = 7.6 Hz, 1H, ArH), 6.96 (d, *J* = 7.6 Hz, 1H, ArH), 6.69–6.63 (m, 3H, ArH + NH), 5.16 (s, 1H, CH), 4.00 (d, *J* = 18.4 Hz, 1H, CH_2), 3.44 (d, *J* = 18.4 Hz, 1H, CH_2), 3.37–3.36 (m, 3H, CH_3), 3.32–3.31 (m, 3H, CH_3), 2.11 (s, 3H, CH_3) ppm. ^{13}C NMR (100 MHz, $CDCl_3$): δ 202.3, 168.0, 166.0, 165.8, 152.5, 150.5, 138.7, 136.5, 134.0, 131.7, 129.4, 129.2, 128.8, 128.0, 126.5, 125.1, 124.8, 68.8, 65.3, 55.3, 38.1, 29.5, 29.0, 21.3 ppm. HRMS (ESI): *m/z* calcd. for $C_{24}H_{22}N_3O_5$ $[M + H]^+$ 432.1554, found 432.1545.

3. Characterization data of compound 5

(2*S*,3'*S*)-1'',3''-Dimethyl-5'-(methylthio)-3'-(*m*-tolyl)-2''*H*,3'*H*-dispiro[indene-2,2'-pyrrole-4',5''-pyrimidine]-1,2'',4'',6''(1''*H*,3*H*,3''*H*)-tetraone (5). From **3ah** (44.8 mg, 0.10 mmol, 1.0 equiv.), dry K_2CO_3 (21.0 mg, 0.23 mmol, 1.50 equiv.) and iodomethane (12.5 μl, 0.20 mmol, 2.0 equiv.), purified by silica gel (200–300 mesh) column chromatography using ethyl acetate/petroleum ether (1:4) as eluent to obtain 44.0 mg (95% yield) compound **5** as a white solid, m.p. 202–204 °C. HPLC (Daicel Chiralpak ADH, *n*-hexane/2-propanol 70:30, flow rate 1.0 mL/min, detection at 254 nm): t_R = 12.8 min (minor), t_R = 21.2 min (major); 99% ee. $[\alpha]_D^{25}$ = +64.8° (*c* = 1.10, THF). ^{1}H NMR (400 MHz, $CDCl_3$): δ 7.83 (d, *J* = 7.6 Hz, 1H, ArH), 7.60–7.56 (m, 1H, ArH), 7.41 (d, *J* = 7.6 Hz, 1H, ArH), 7.36 (t, *J* = 7.4 Hz, 1H, ArH), 7.07 (t, *J* = 7.6 Hz, 1H, ArH), 6.99 (d, *J* = 7.6 Hz, 1H, ArH), 6.78–6.75 (m, 2H, ArH), 5.18 (s, 1H, CH), 3.67 (d, *J* = 17.6 Hz, 1H, CH_2), 3.40 (d, *J* = 17.6 Hz, 1H, CH_2), 3.33 (s, 3H, CH_3), 3.32 (s, 3H, CH_3), 2.44 (s, 3H, CH_3), 2.18 (s, 3H, CH_3) ppm. ^{13}C NMR (100 MHz, $CDCl_3$): δ 202.5, 167.6, 166.6, 166.3, 153.1, 150.2, 138.7, 135.6, 134.5, 132.7, 130.0, 129.1, 128.7, 127.6, 126.5, 125.8, 125.0, 86.3, 73.9, 61.0, 37.4, 29.4, 28.9, 21.3, 14.1 ppm. HRMS (ESI): *m*/*z* calcd. for $C_{25}H_{24}N_3O_4S$ $[M + H]^+$ 462.1482, found 462.1478.

4. Copies of ^{1}H and ^{13}C spectra of new compounds

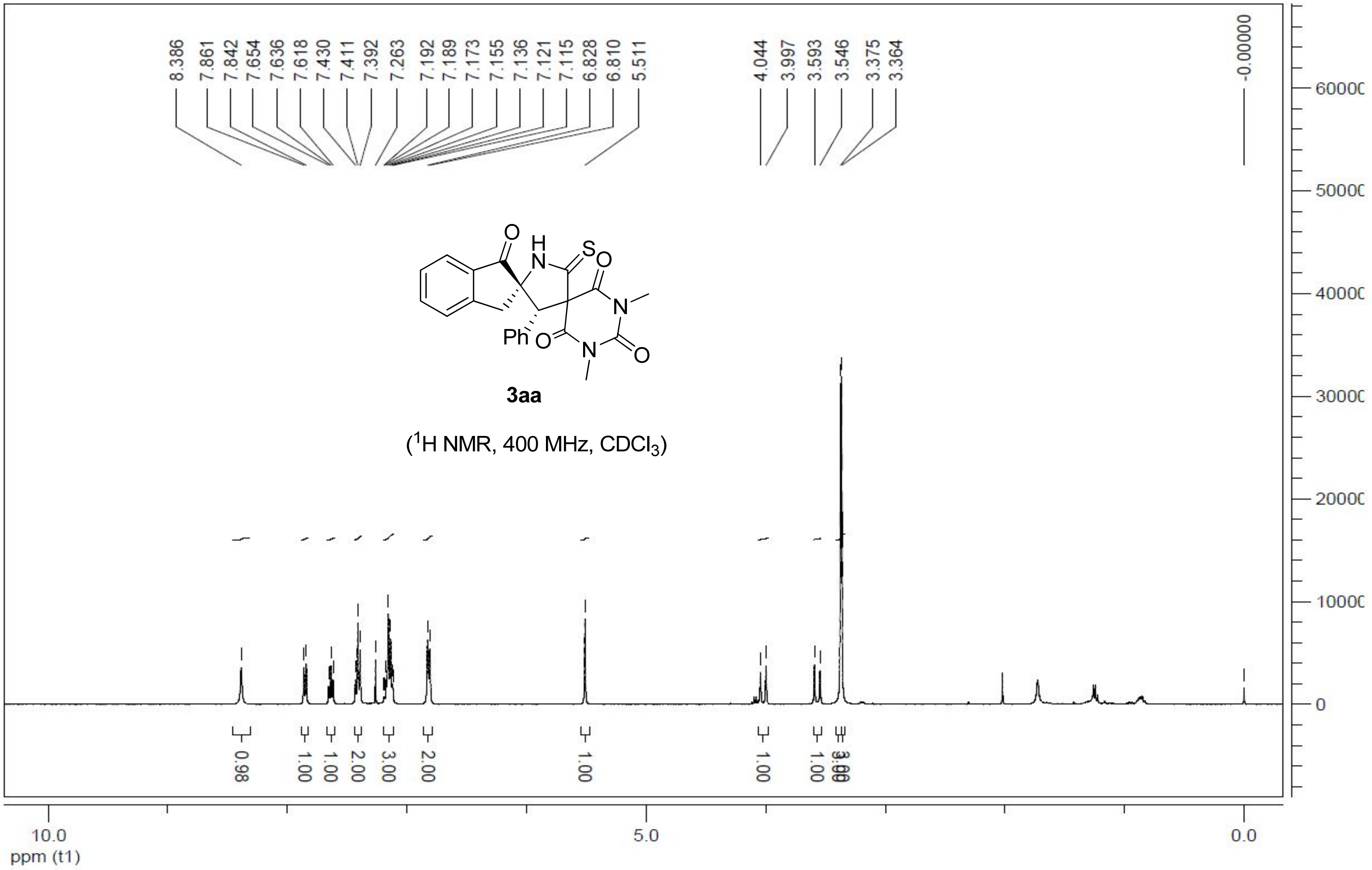

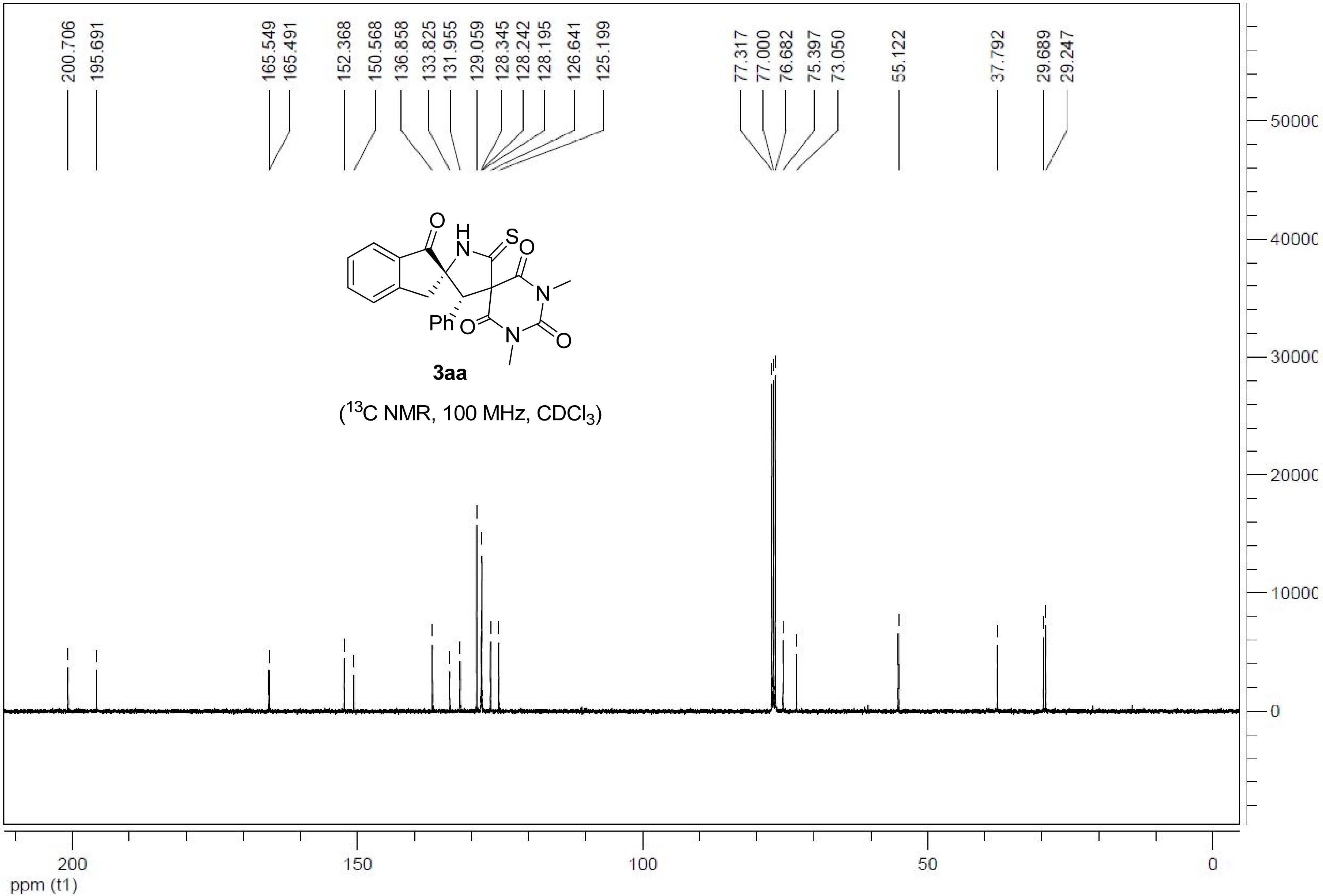
200.706
195.691
165.549
165.491
152.368
150.568
136.858
133.825
131.955
129.059
128.345
128.242
128.195
126.641
125.199
77.317
77.000
76.682
75.397
73.050
55.122
37.792
29.689
29.247
3aa
(13C NMR, 100 MHz, CDCl3)
50000
40000
30000
20000
10000
0
200
150
100
50
0
ppm (t1)

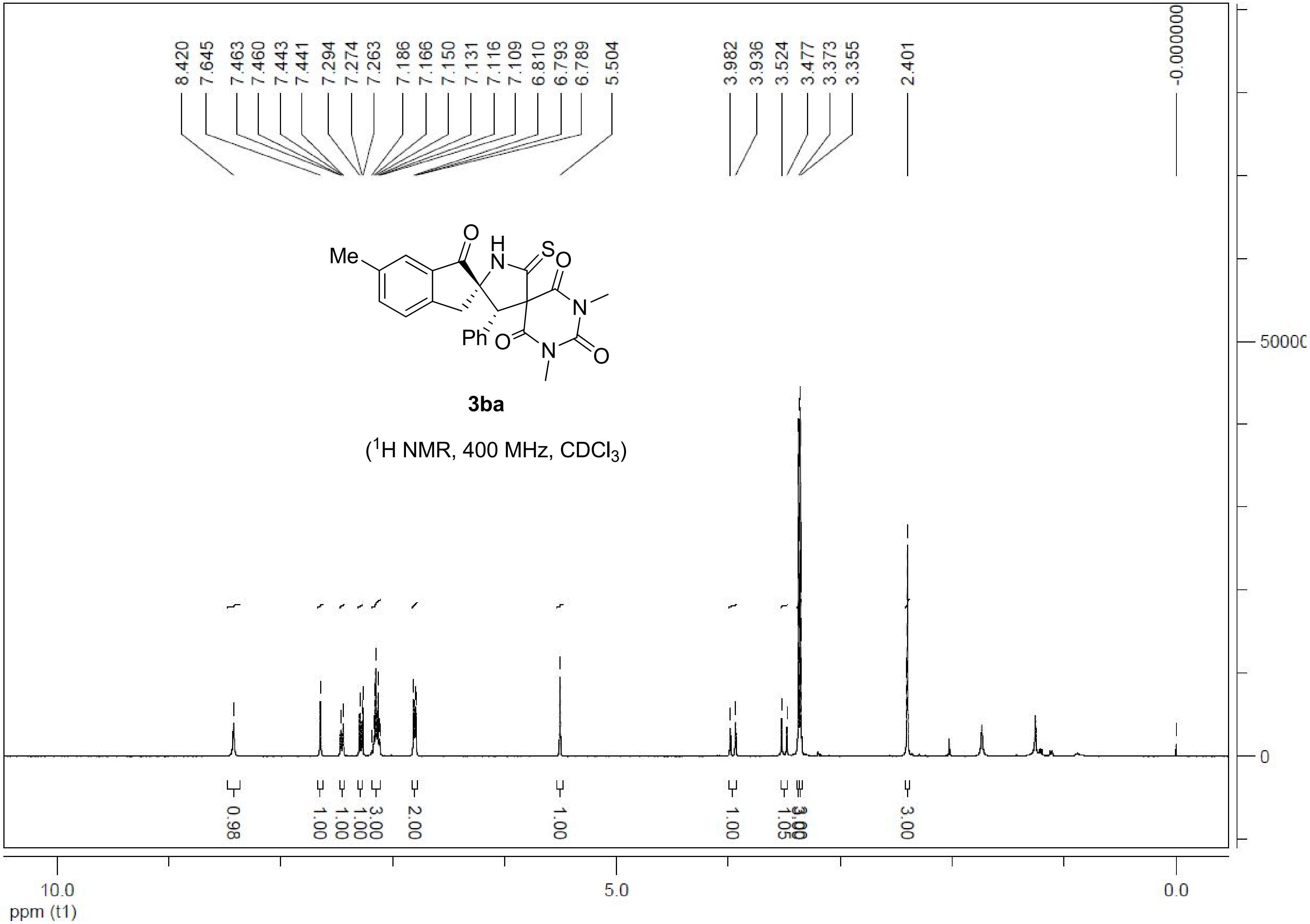
8.420
7.645
7.463
7.460
7.443
7.441
7.294
7.274
7.263
7.186
7.166
7.150
7.131
7.116
7.109
6.810
6.793
6.789
5.504
3.982
3.936
3.524
3.477
3.373
3.355
2.401
-0.000000
Me
O
H
N
S
O
N
Ph
O
N
O
3ba
(1H NMR, 400 MHz, CDCl3)
50000
0
0.98
1.00
1.00
1.00
3.00
2.00
1.00
1.00
1.05
3.00
3.00
10.0
ppm (t1)
5.0
0.0

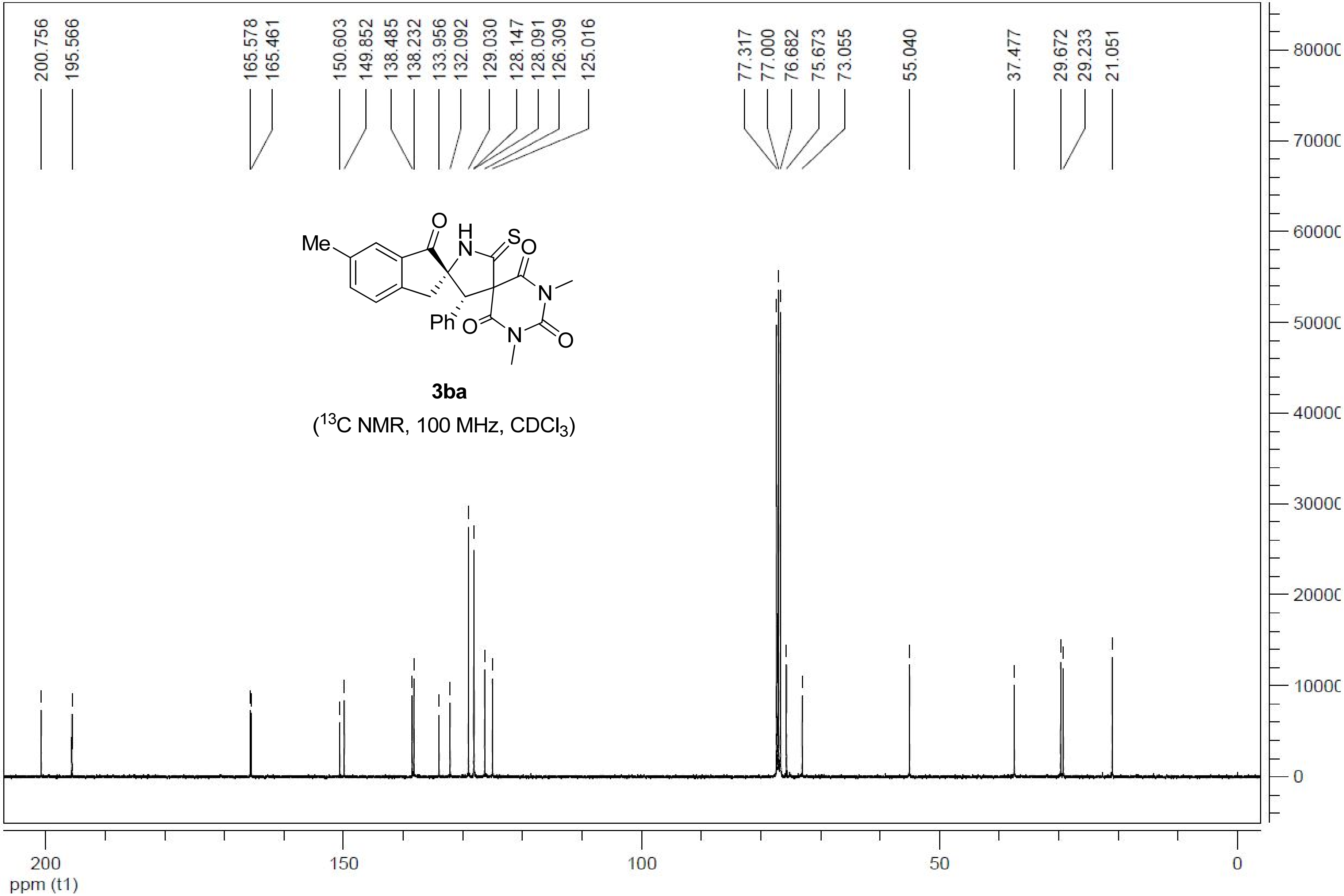
200.756
195.566
165.578
165.461
150.603
149.852
138.485
138.232
133.956
132.092
129.030
128.147
128.091
126.309
125.016
77.317
77.000
76.682
75.673
73.055
55.040
37.477
29.672
29.233
21.051
Me
O
H
N
S
O
N
Ph
O
N
O
3ba
(13C NMR, 100 MHz, CDCl3)
200
150
100
50
0
ppm (t1)

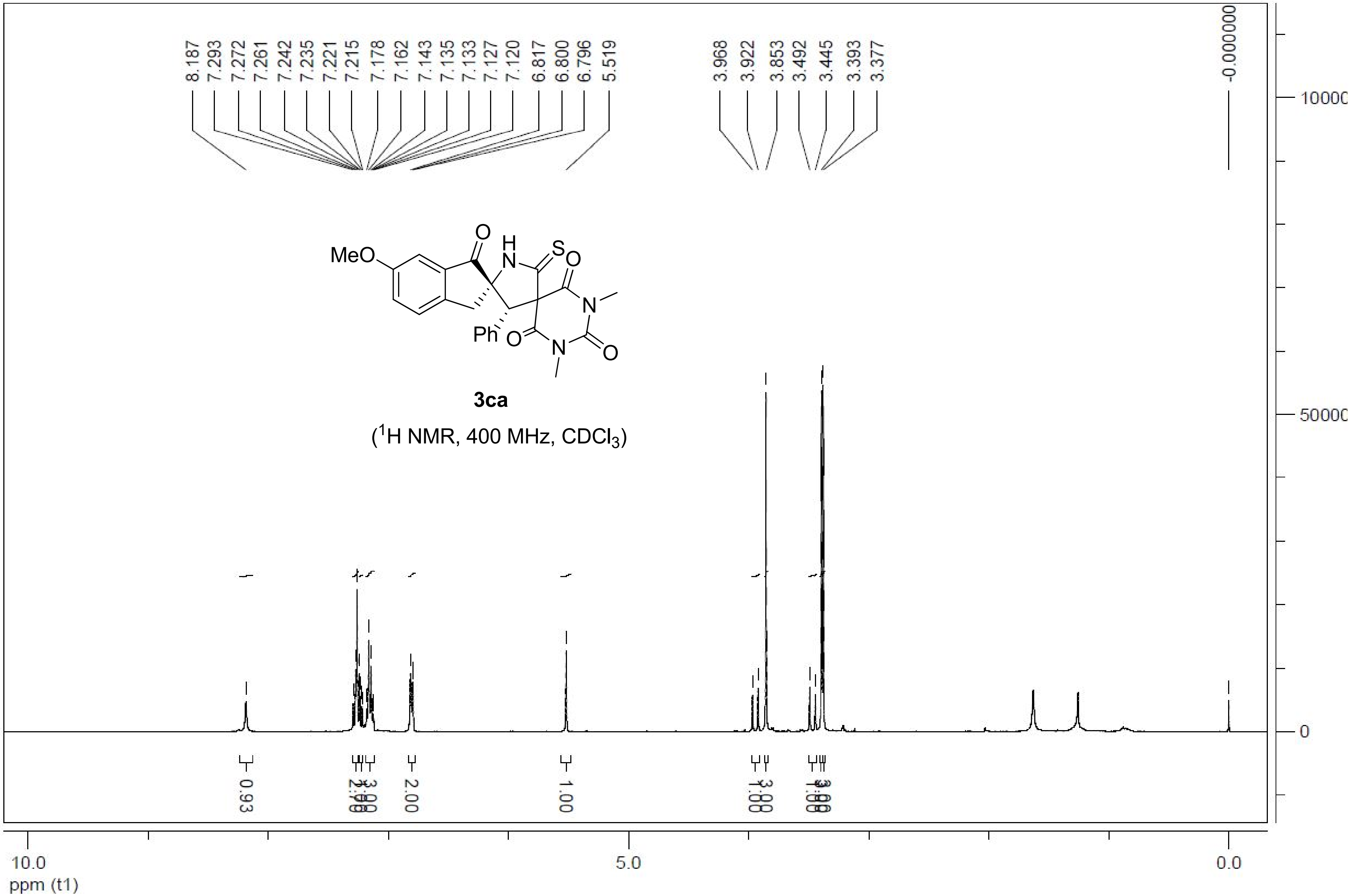

8.187
7.293
7.272
7.261
7.242
7.235
7.221
7.215
7.178
7.162
7.143
7.135
7.133
7.127
7.120
6.817
6.800
6.796
5.519
3.968
3.922
3.853
3.492
3.445
3.393
3.377
-0.000000
MeO
O
H
N
S
O
N
Ph
O
N
O
3ca
(^{1}H NMR, 400 MHz, $CDCl_3$)
0.93
2.00
1.00
10000
50000
0
10.0
5.0
0.0
ppm (t1)

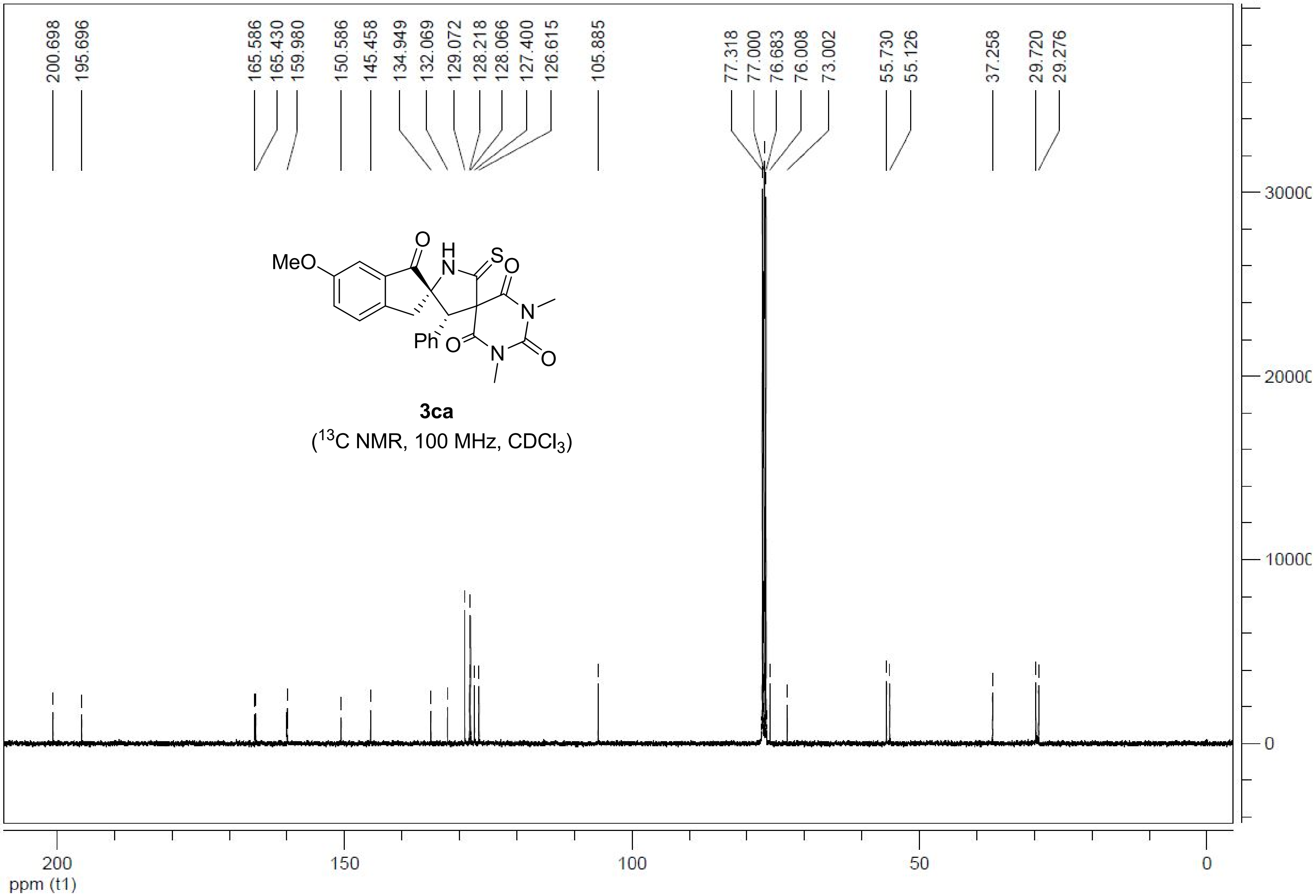

3ca

(^{13}C NMR, 100 MHz, $CDCl_3$)

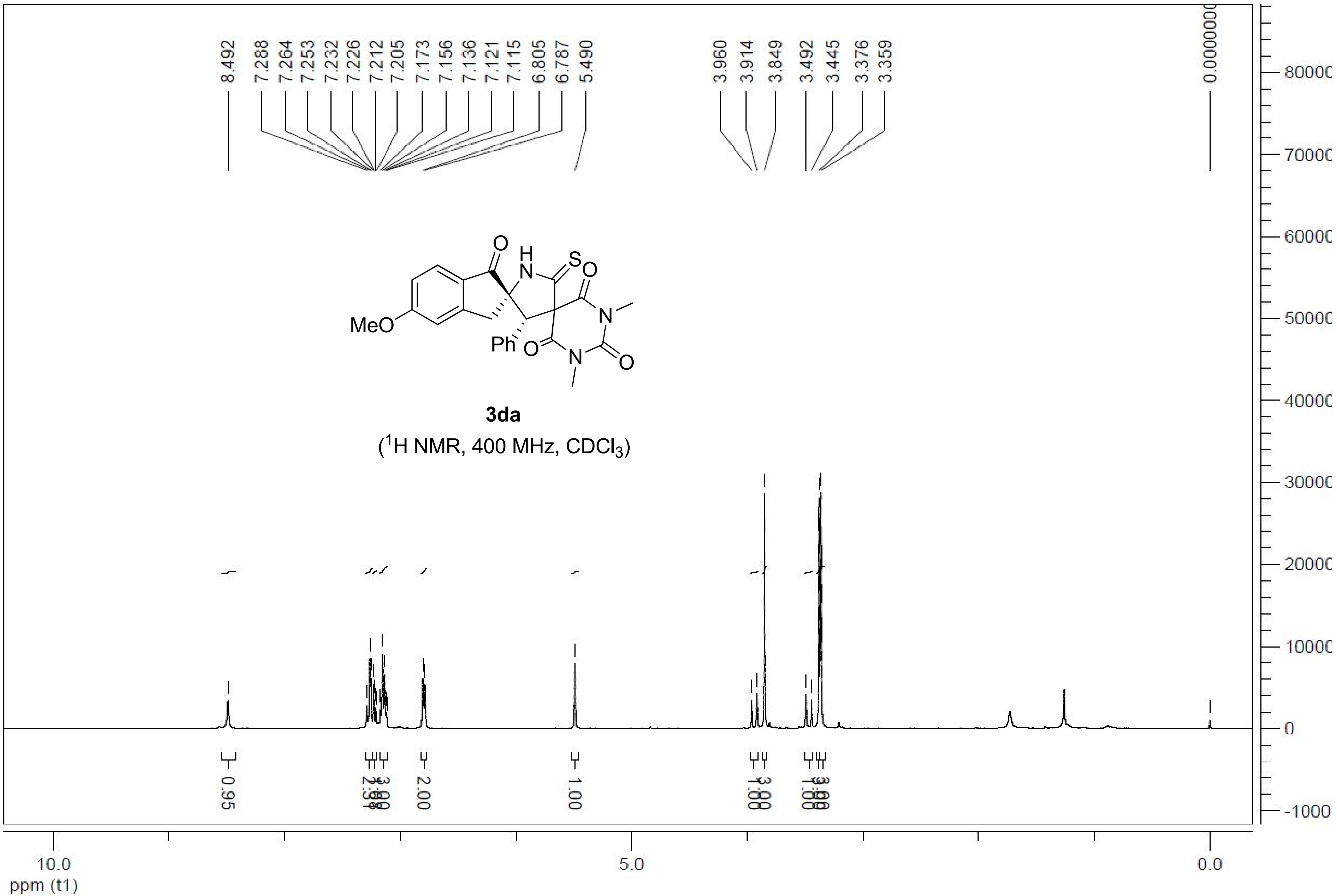

8.492
7.288
7.264
7.253
7.232
7.226
7.212
7.205
7.173
7.156
7.136
7.121
7.115
6.805
6.787
5.490
3.960
3.914
3.849
3.492
3.445
3.376
3.359
0.000000
MeO
Ph
3da
(¹H NMR, 400 MHz, CDCl₃)
0.95
2.00
1.00
10.0
5.0
0.0
ppm (t1)

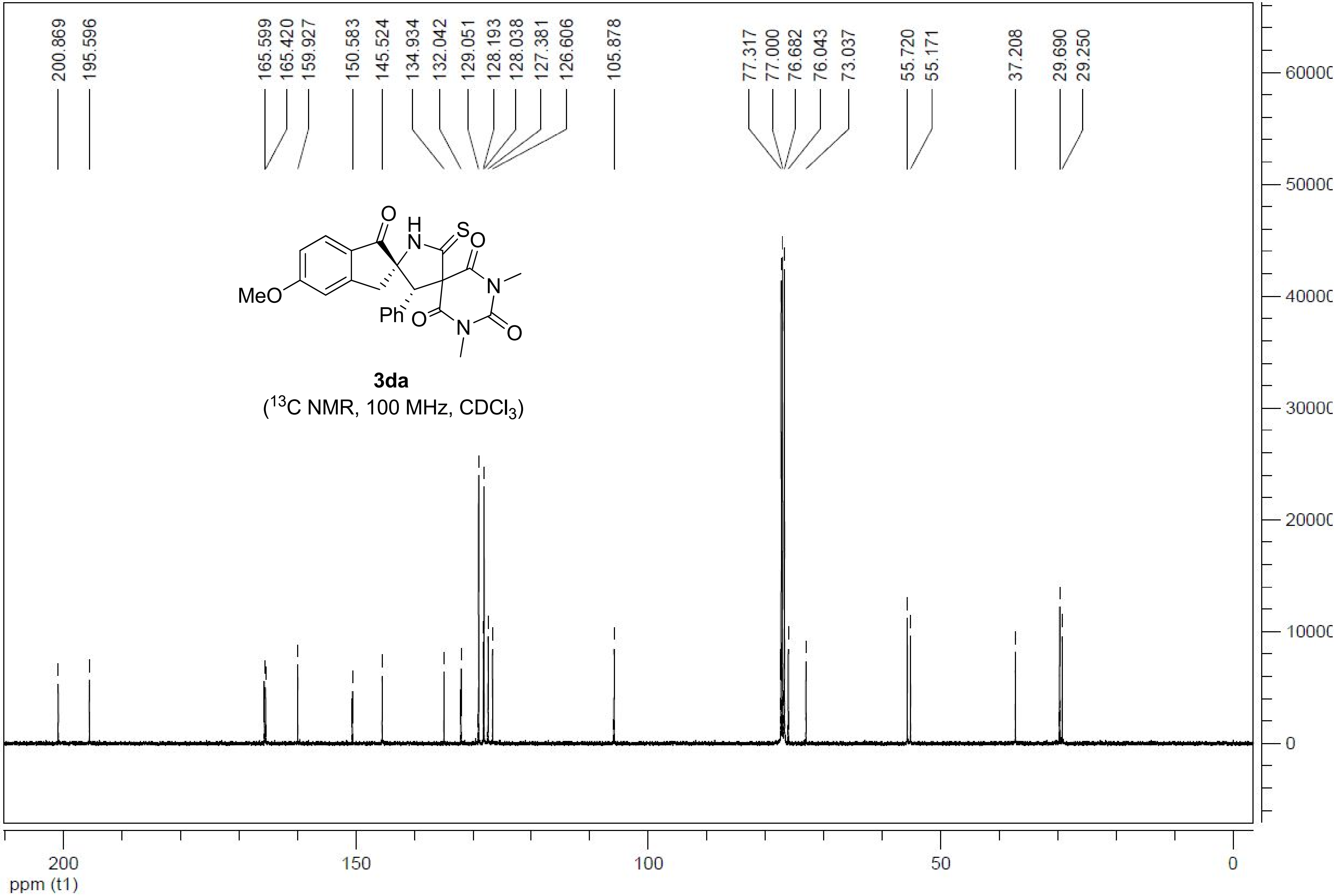

3da

(^{13}C NMR, 100 MHz, $CDCl_3$)

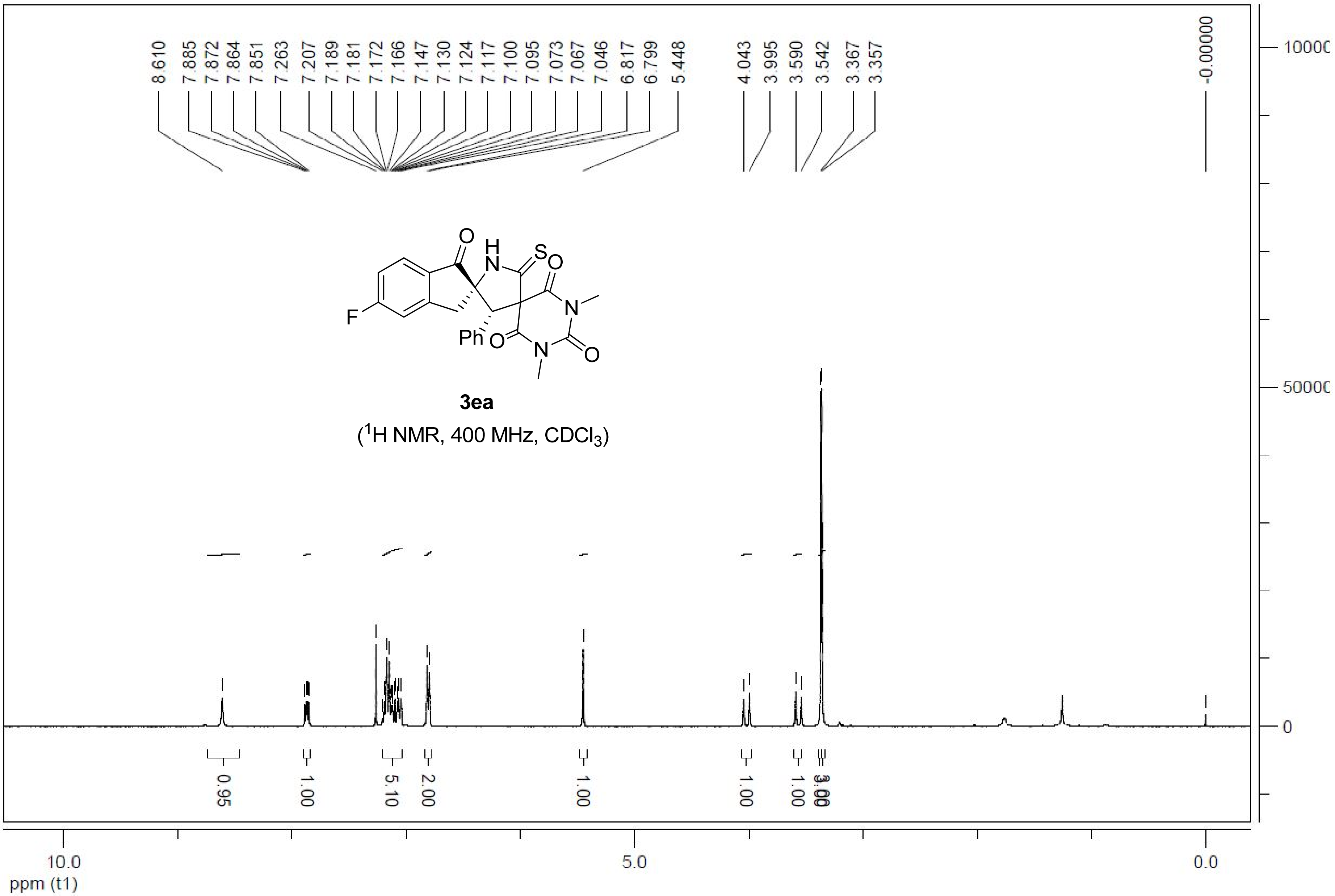

8.610
7.885
7.872
7.864
7.851
7.263
7.207
7.189
7.181
7.172
7.166
7.147
7.130
7.124
7.117
7.100
7.095
7.073
7.067
7.046
6.817
6.799
5.448
4.043
3.995
3.590
3.542
3.367
3.357
-0.00000
3ea
(¹H NMR, 400 MHz, CDCl₃)
0.95
1.00
5.10
2.00
1.00
1.00
1.00
10.0
5.0
0.0
ppm (t1)

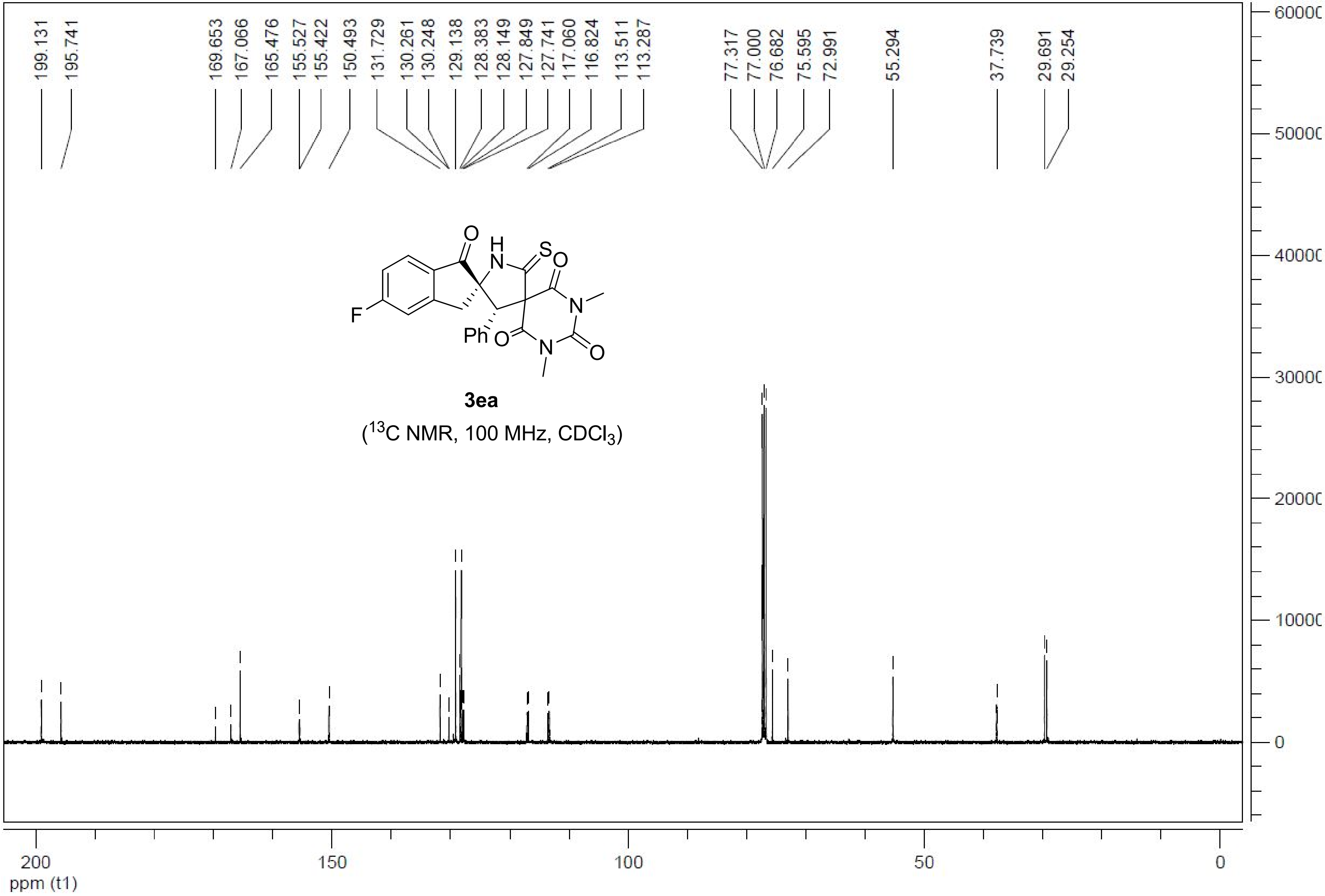

3ea

(^{13}C NMR, 100 MHz, $CDCl_3$)

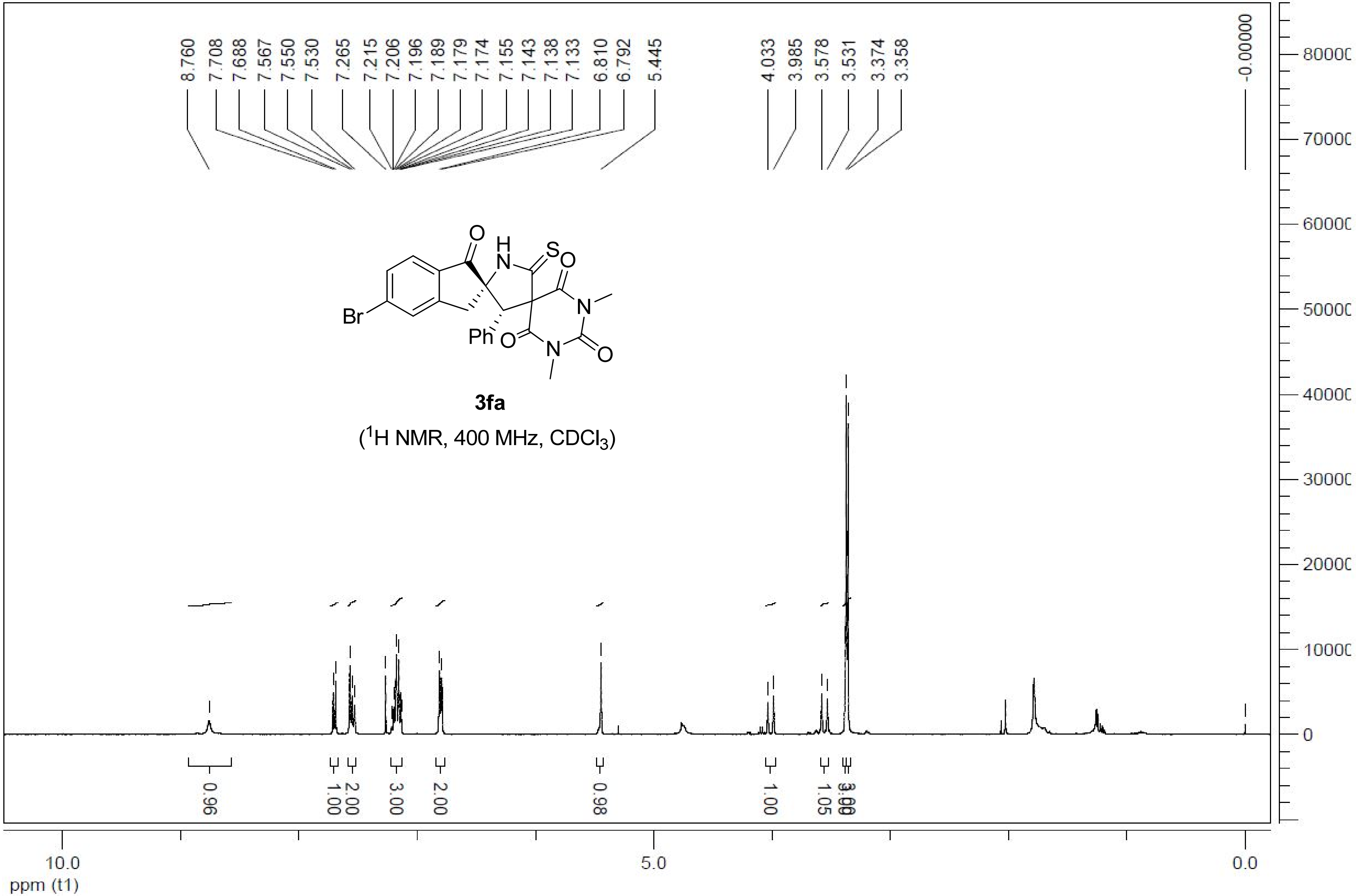

3fa

(^{1}H NMR, 400 MHz, $CDCl_3$)

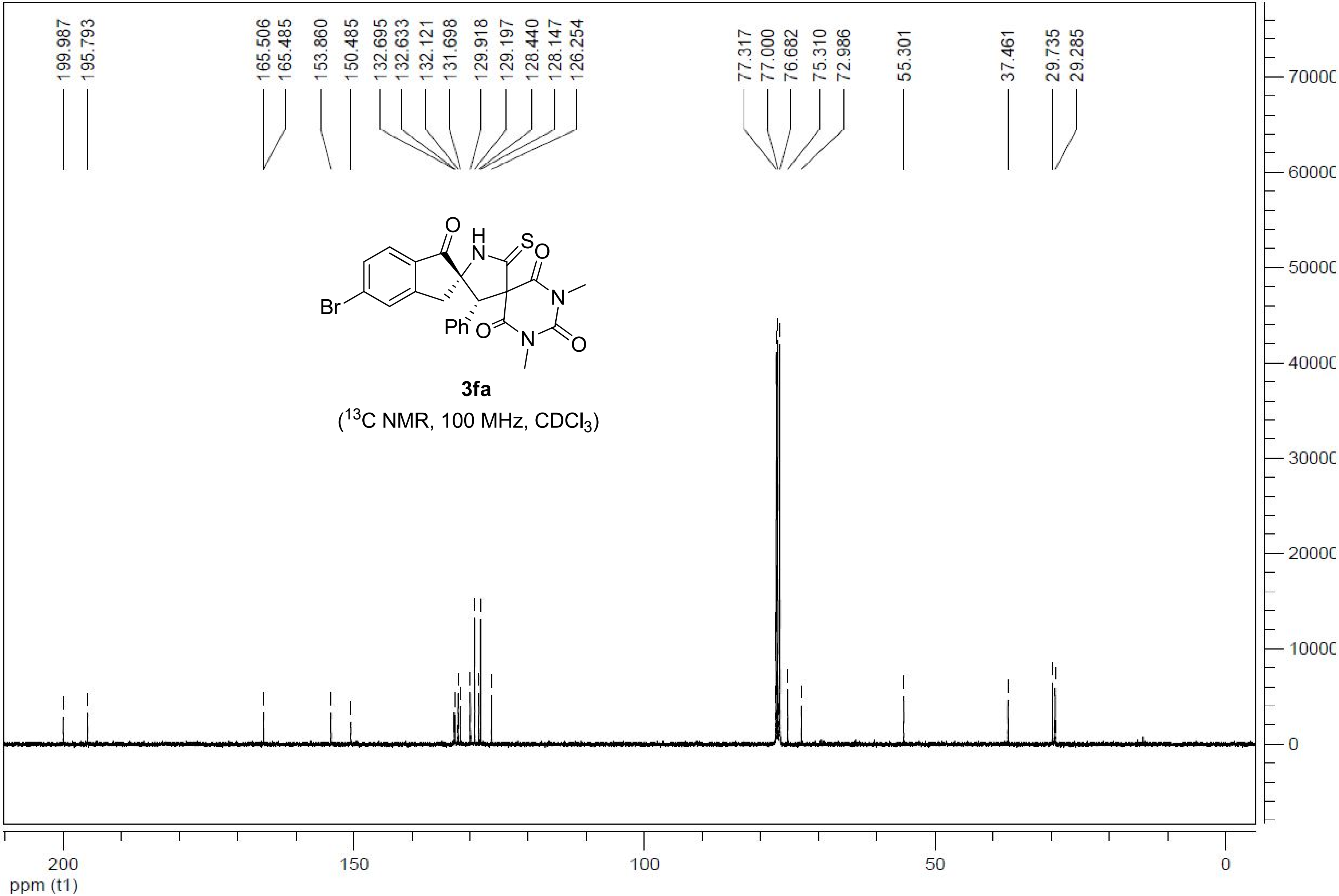

3fa

(^{13}C NMR, 100 MHz, $CDCl_3$)

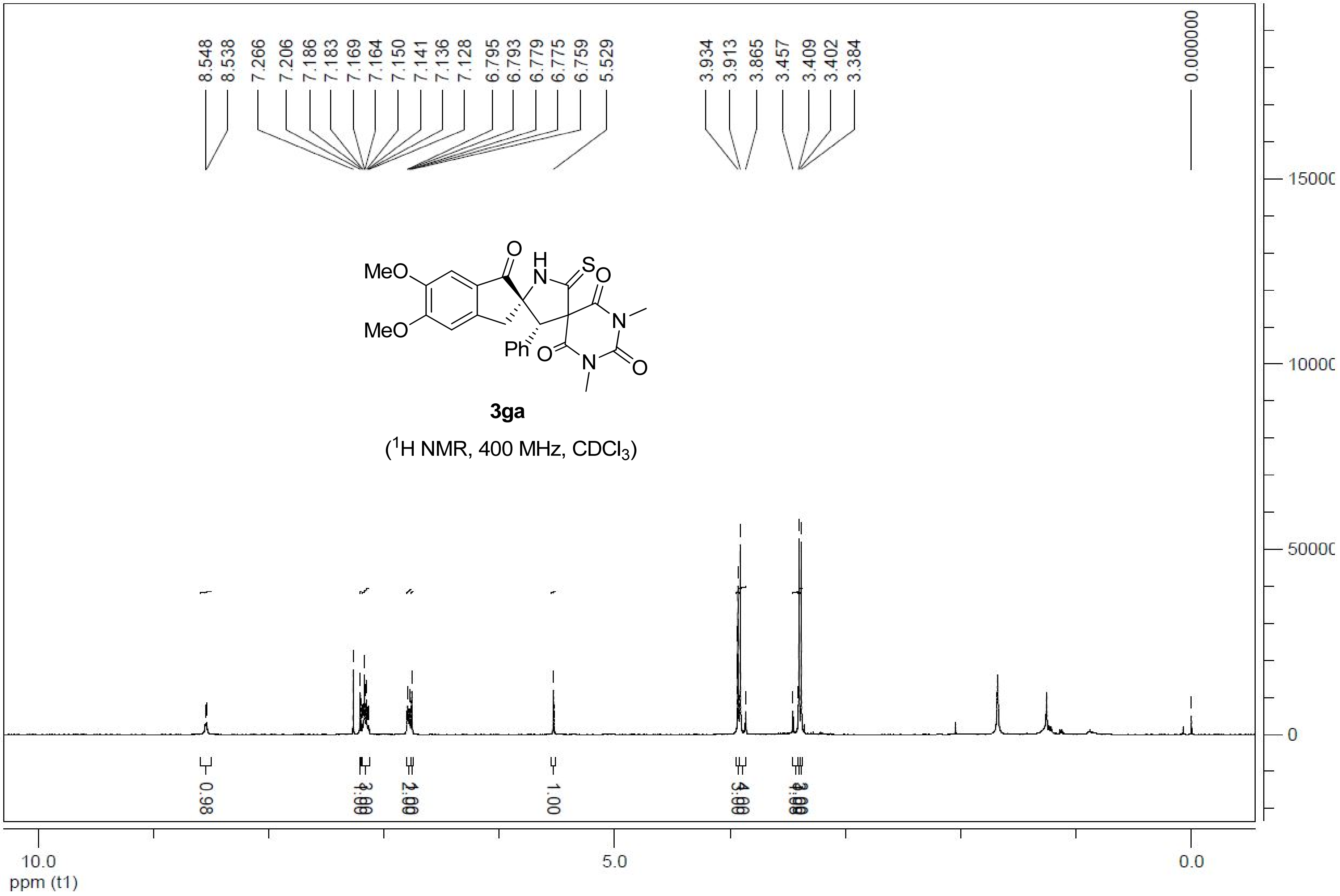

8.548
8.538
7.266
7.206
7.186
7.183
7.169
7.164
7.150
7.141
7.136
7.128
6.795
6.793
6.779
6.775
6.759
5.529
3.934
3.913
3.865
3.457
3.409
3.402
3.384
0.000000
MeO
MeO
O
H
N
S
O
N
Ph
O
N
O
3ga
(1H NMR, 400 MHz, CDCl3)
0.98
1.00
1.00
10.0
5.0
0.0
ppm (t1)
15000
10000
5000
0

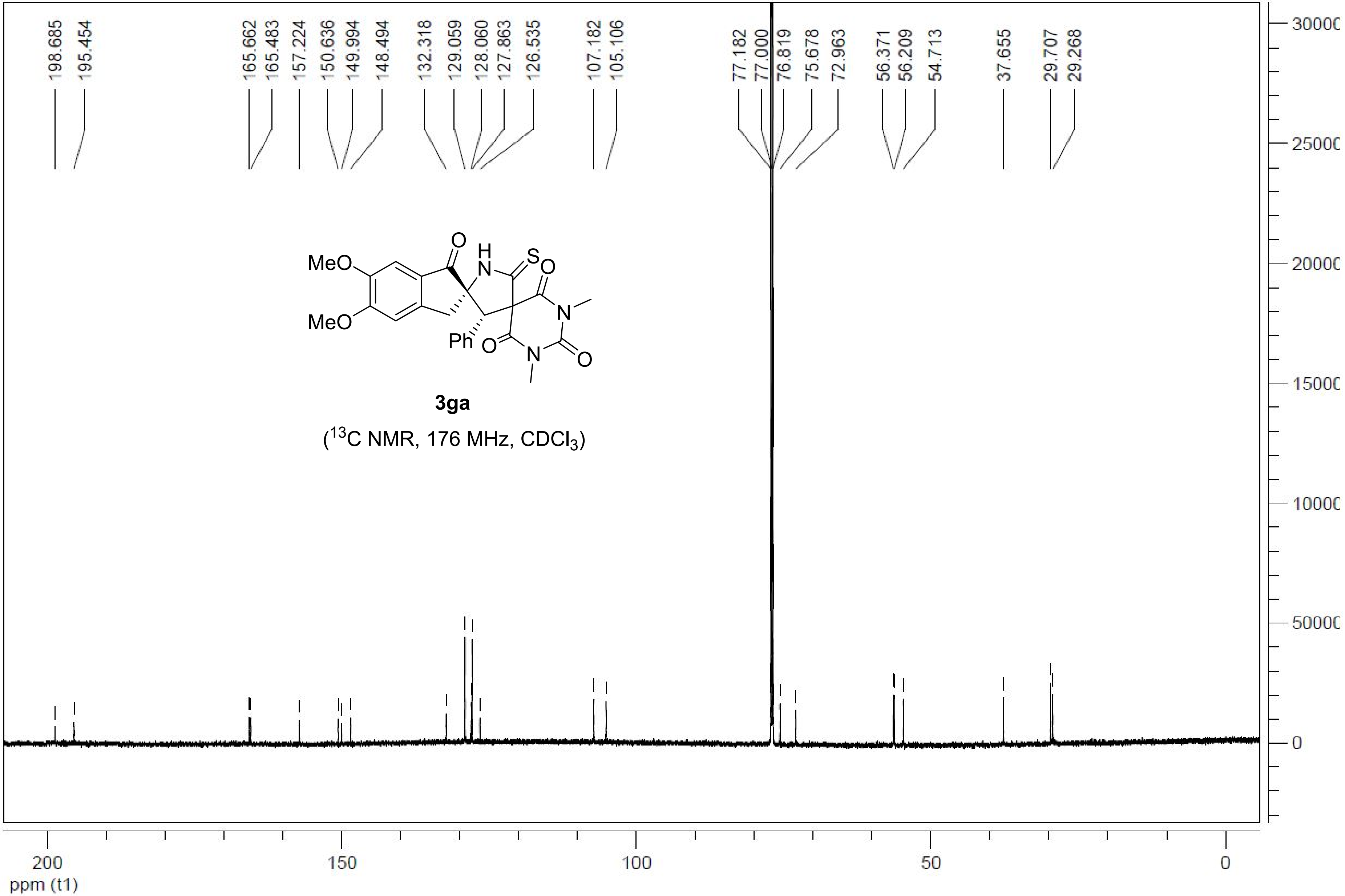

3ga

(^{13}C NMR, 176 MHz, $CDCl_3$)

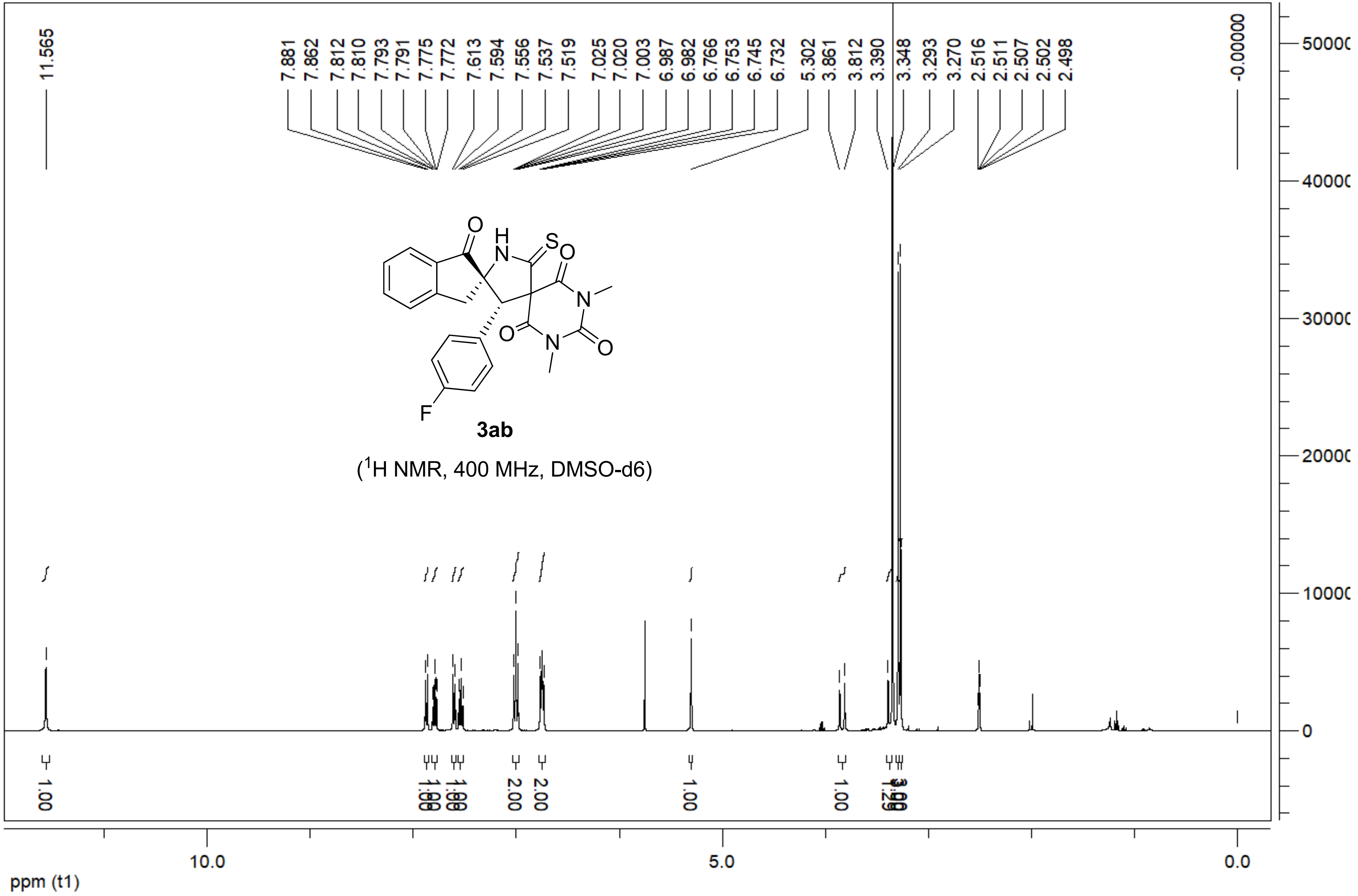
11.565
7.881
7.862
7.812
7.810
7.793
7.791
7.775
7.772
7.613
7.594
7.556
7.537
7.519
7.025
7.020
7.003
6.987
6.982
6.766
6.753
6.745
6.732
5.302
3.861
3.812
3.390
3.348
3.293
3.270
2.516
2.511
2.507
2.502
2.498
-0.00000
3ab
(1H NMR, 400 MHz, DMSO-d6)
1.00
2.00
2.00
1.00
1.00
10.0
5.0
0.0
ppm (t1)

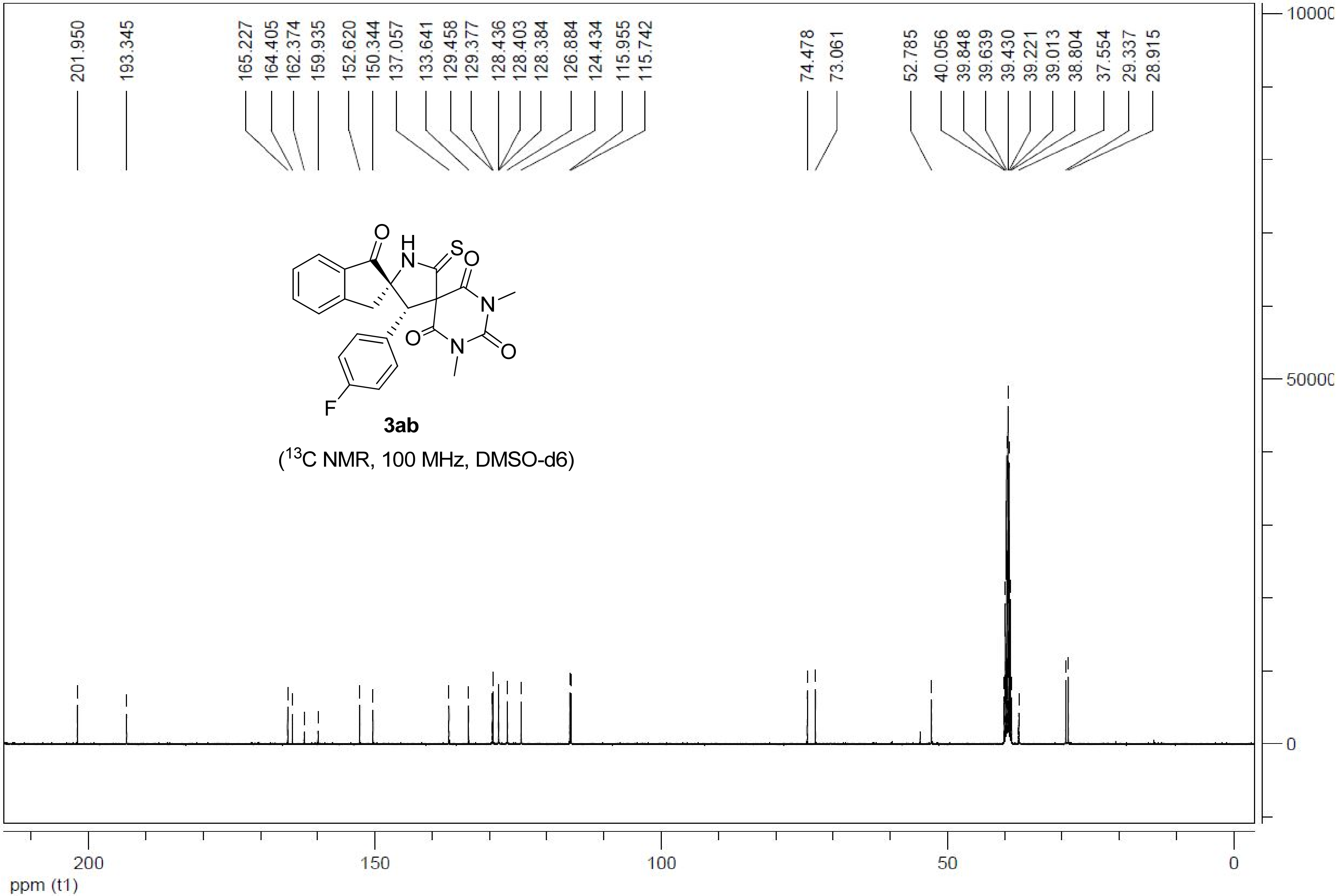

3ab

(^{13}C NMR, 100 MHz, DMSO-d6)

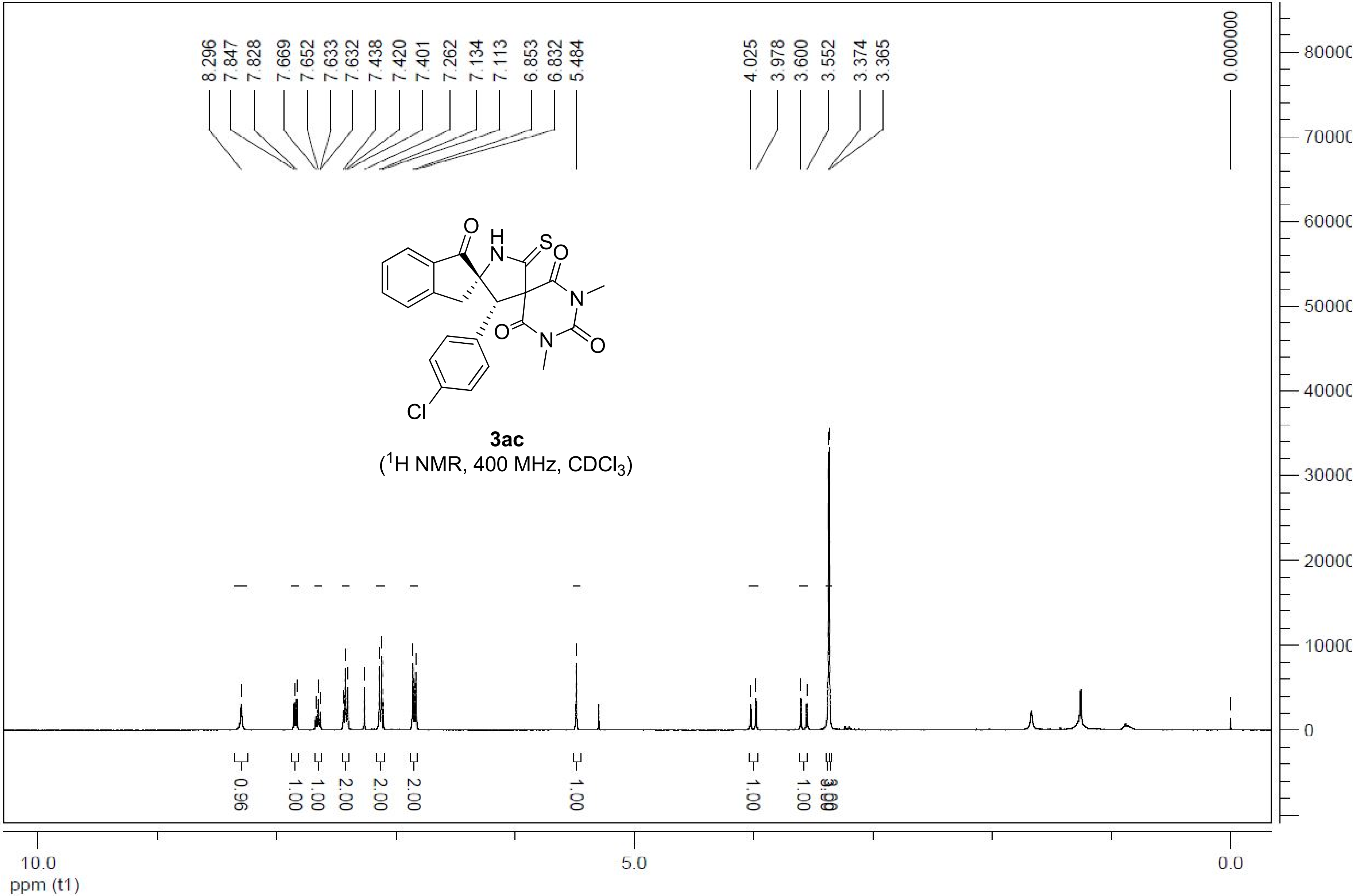

3ac
(^{1}H NMR, 400 MHz, $CDCl_3$)

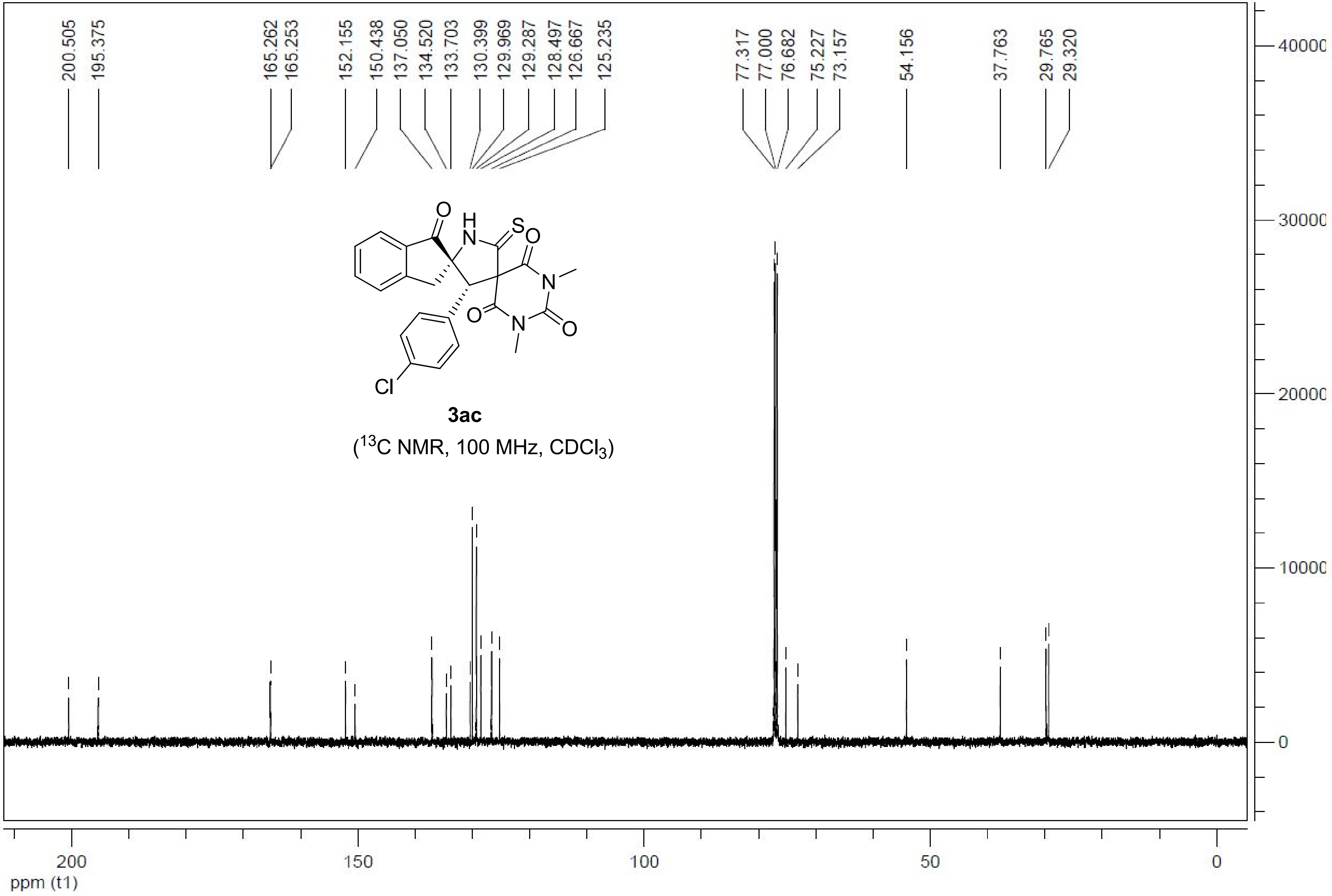
200.505
195.375
165.262
165.253
152.155
150.438
137.050
134.520
133.703
130.399
129.969
129.287
128.497
126.667
125.235
77.317
77.000
76.682
75.227
73.157
54.156
37.763
29.765
29.320
3ac
(^{13}C NMR, 100 MHz, CDCl$_3$)
40000
30000
20000
10000
0
200
150
100
50
0
ppm (t1)

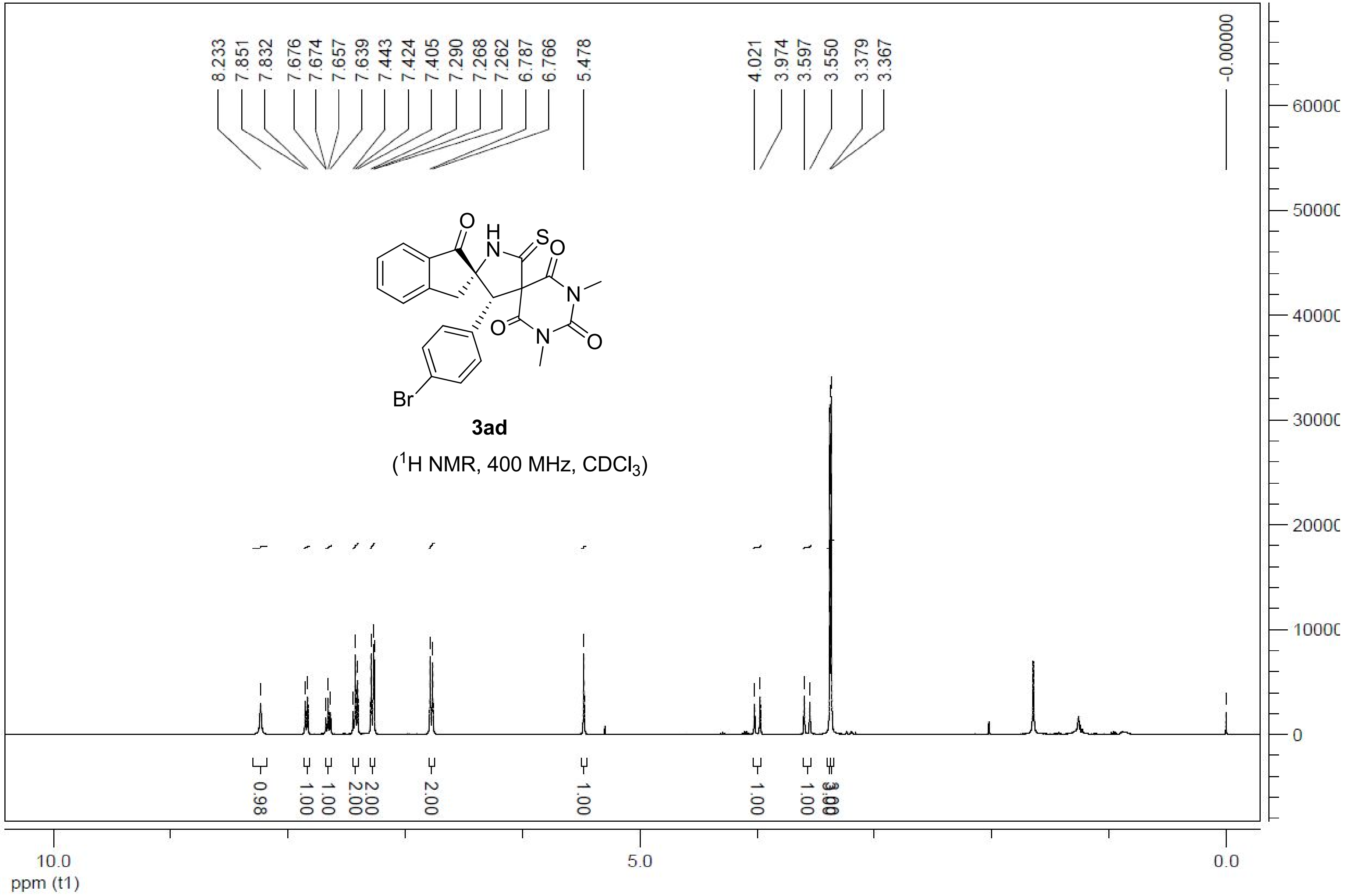

8.233
7.851
7.832
7.676
7.674
7.657
7.639
7.443
7.424
7.405
7.290
7.268
7.262
6.787
6.766
5.478
4.021
3.974
3.597
3.550
3.379
3.367
-0.00000
3ad
(¹H NMR, 400 MHz, CDCl₃)
0.98
1.00
1.00
2.00
2.00
2.00
1.00
1.00
1.00
10.0
5.0
0.0
ppm (t1)

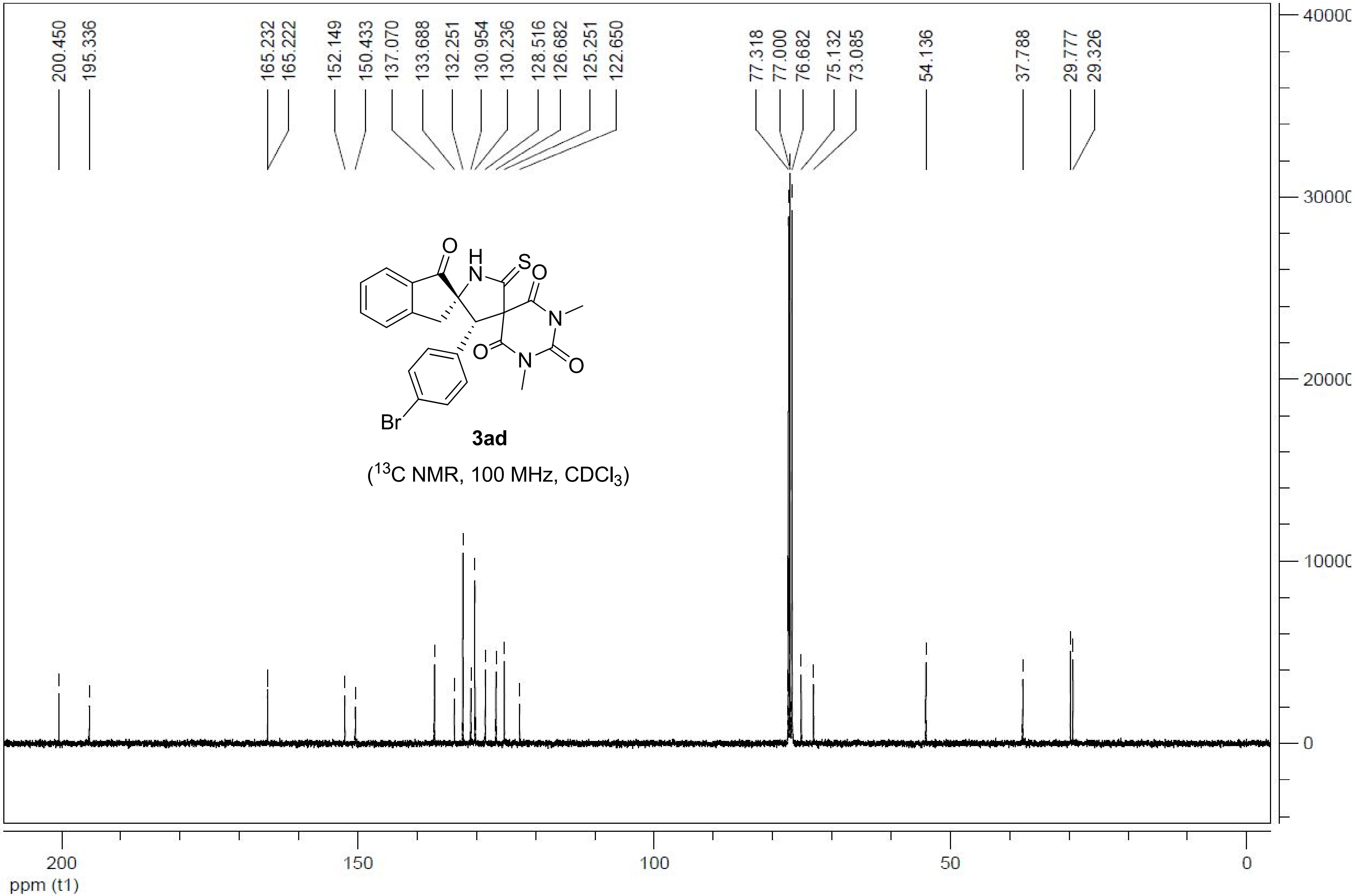

3ad

(^{13}C NMR, 100 MHz, $CDCl_3$)

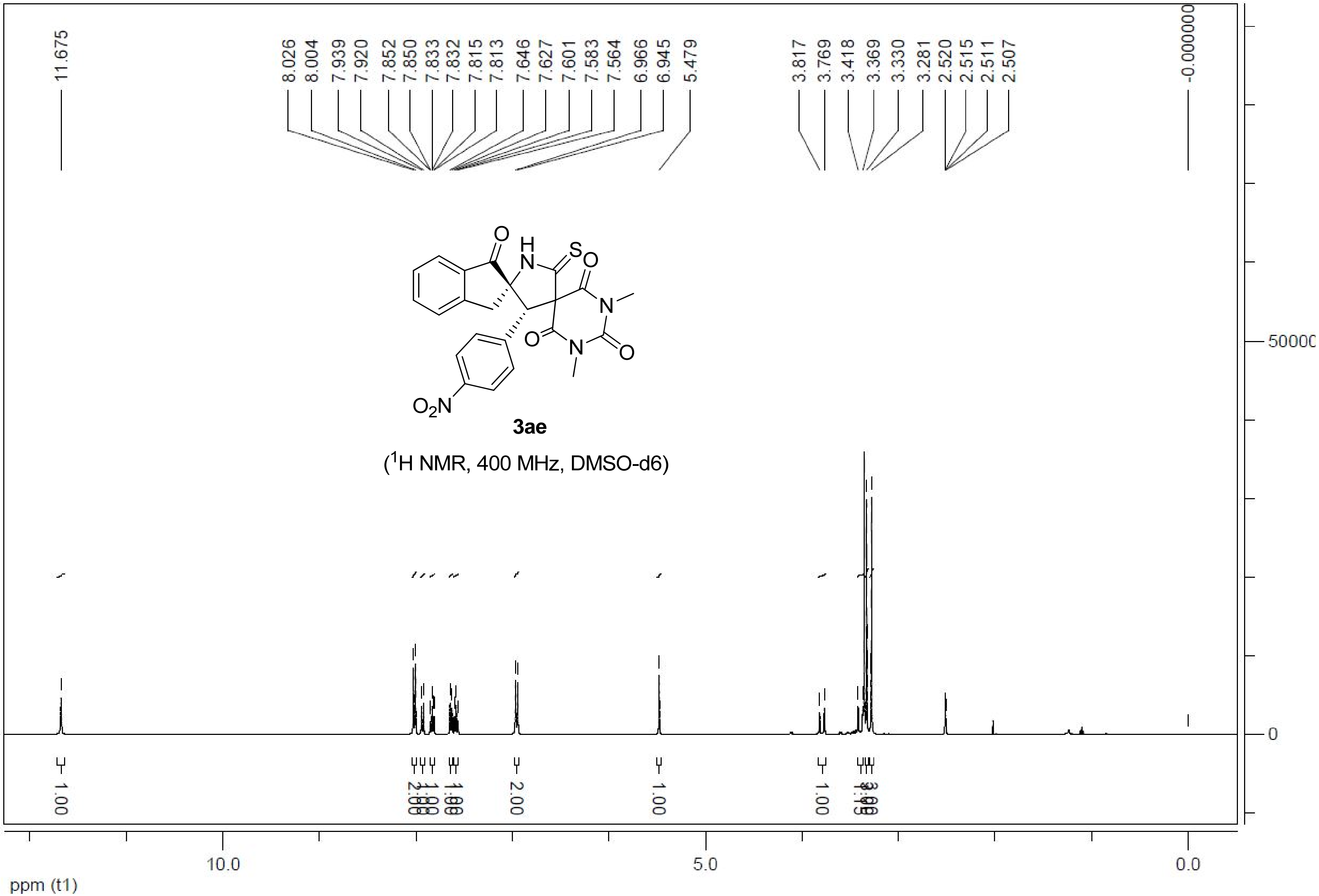
11.675
8.026
8.004
7.939
7.920
7.852
7.850
7.833
7.832
7.815
7.813
7.646
7.627
7.601
7.583
7.564
6.966
6.945
5.479
3.817
3.769
3.418
3.369
3.330
3.281
2.520
2.515
2.511
2.507
-0.000000
O
H
N
S
O
N
O
N
O
O2N
3ae
(1H NMR, 400 MHz, DMSO-d6)
50000
0
1.00
2.00
1.00
1.00
10.0
5.0
0.0
ppm (t1)

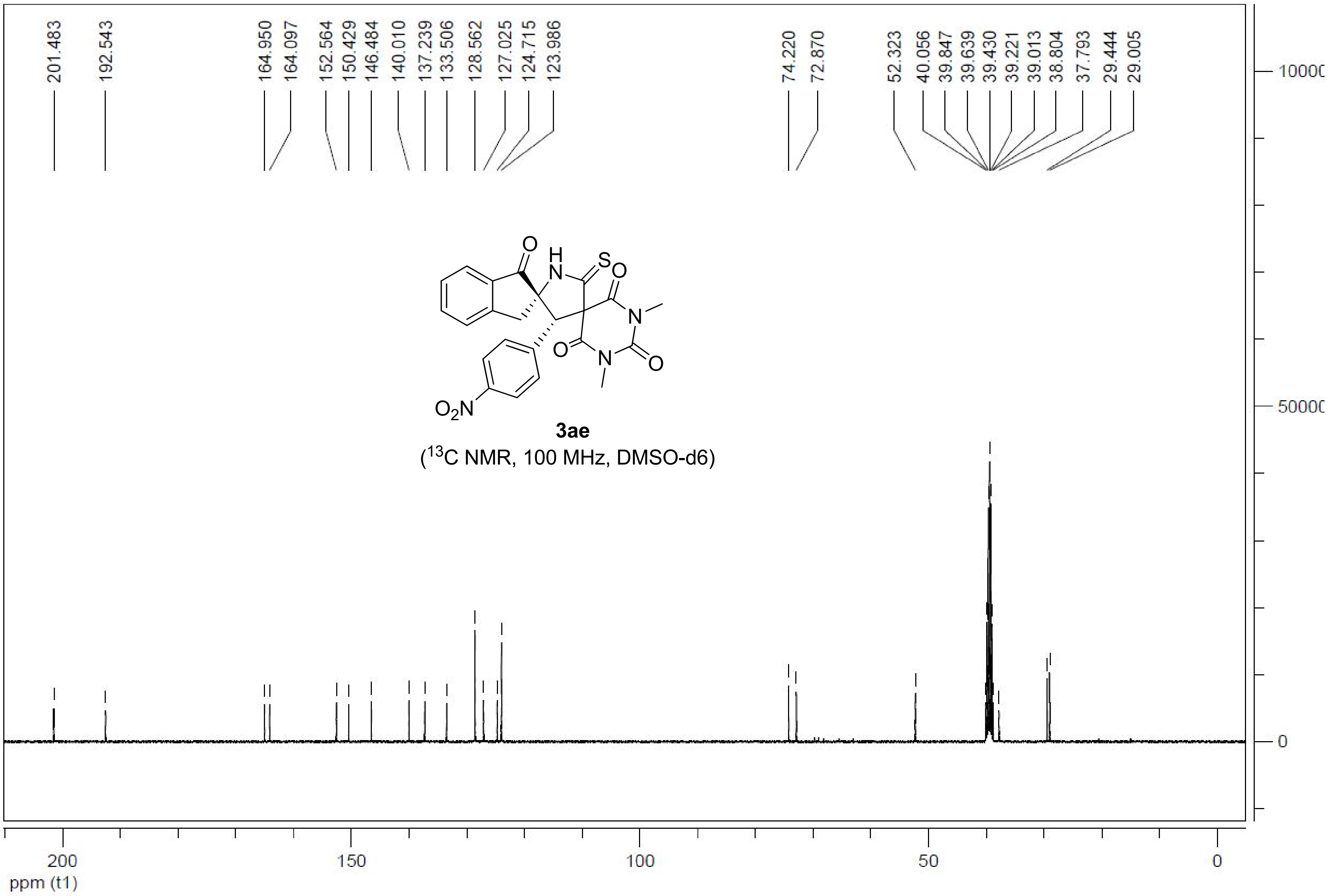
201.483
192.543
164.950
164.097
152.564
150.429
146.484
140.010
137.239
133.506
128.562
127.025
124.715
123.986
74.220
72.870
52.323
40.056
39.847
39.639
39.430
39.221
39.013
38.804
37.793
29.444
29.005
O
H
N
S
O
N
O
N
O
O2N
3ae
(13C NMR, 100 MHz, DMSO-d6)
200
150
100
50
0
ppm (t1)
0
50000
10000

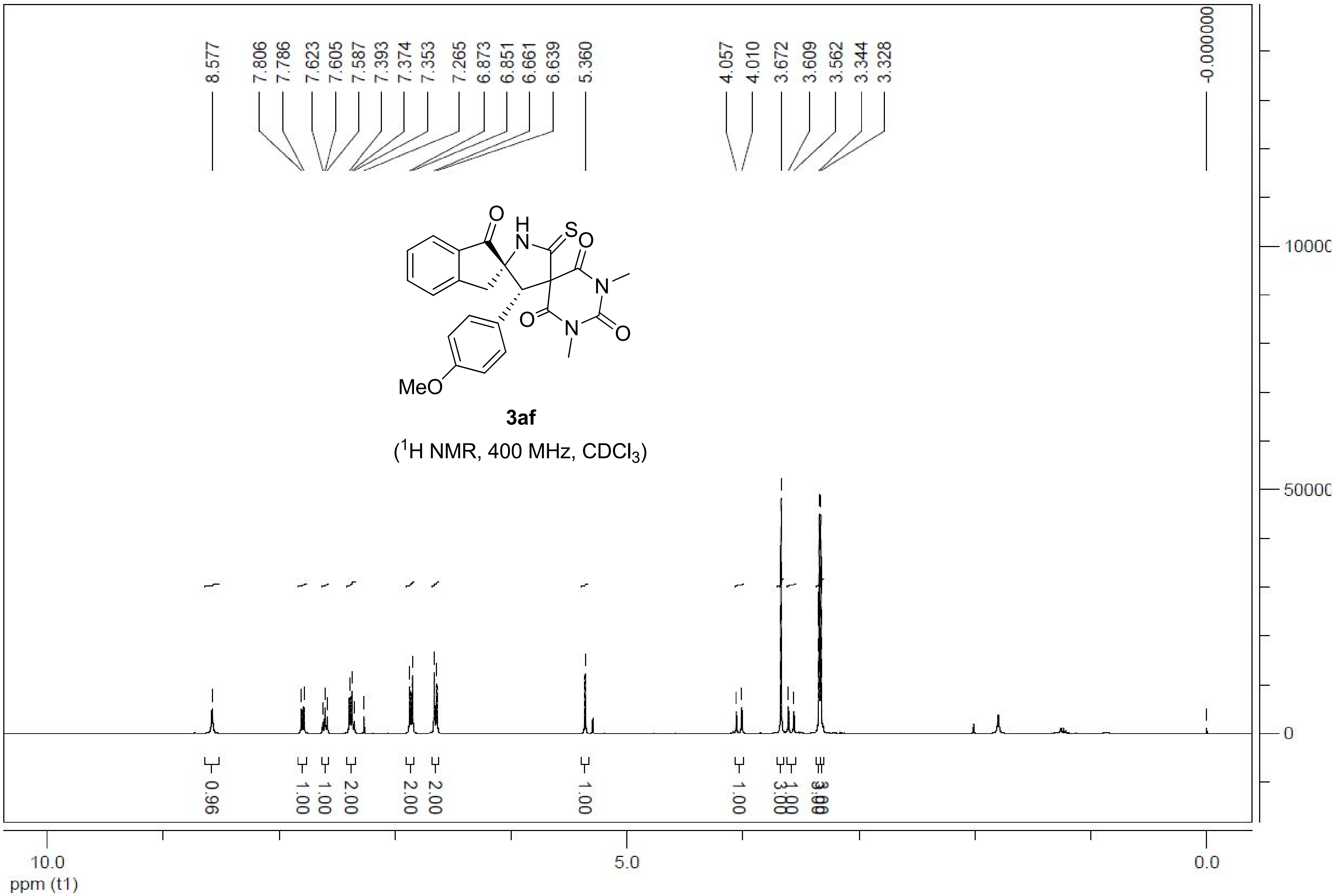
8.577
7.806
7.786
7.623
7.605
7.587
7.393
7.374
7.353
7.265
6.873
6.851
6.661
6.639
5.360
4.057
4.010
3.672
3.609
3.562
3.344
3.328
-0.000000
O
H
N
S
O
O
N
N
O
O
MeO
3af
(1H NMR, 400 MHz, CDCl3)
100000
50000
0
0.96
1.00
1.00
2.00
2.00
2.00
1.00
1.00
10.0
5.0
0.0
ppm (t1)

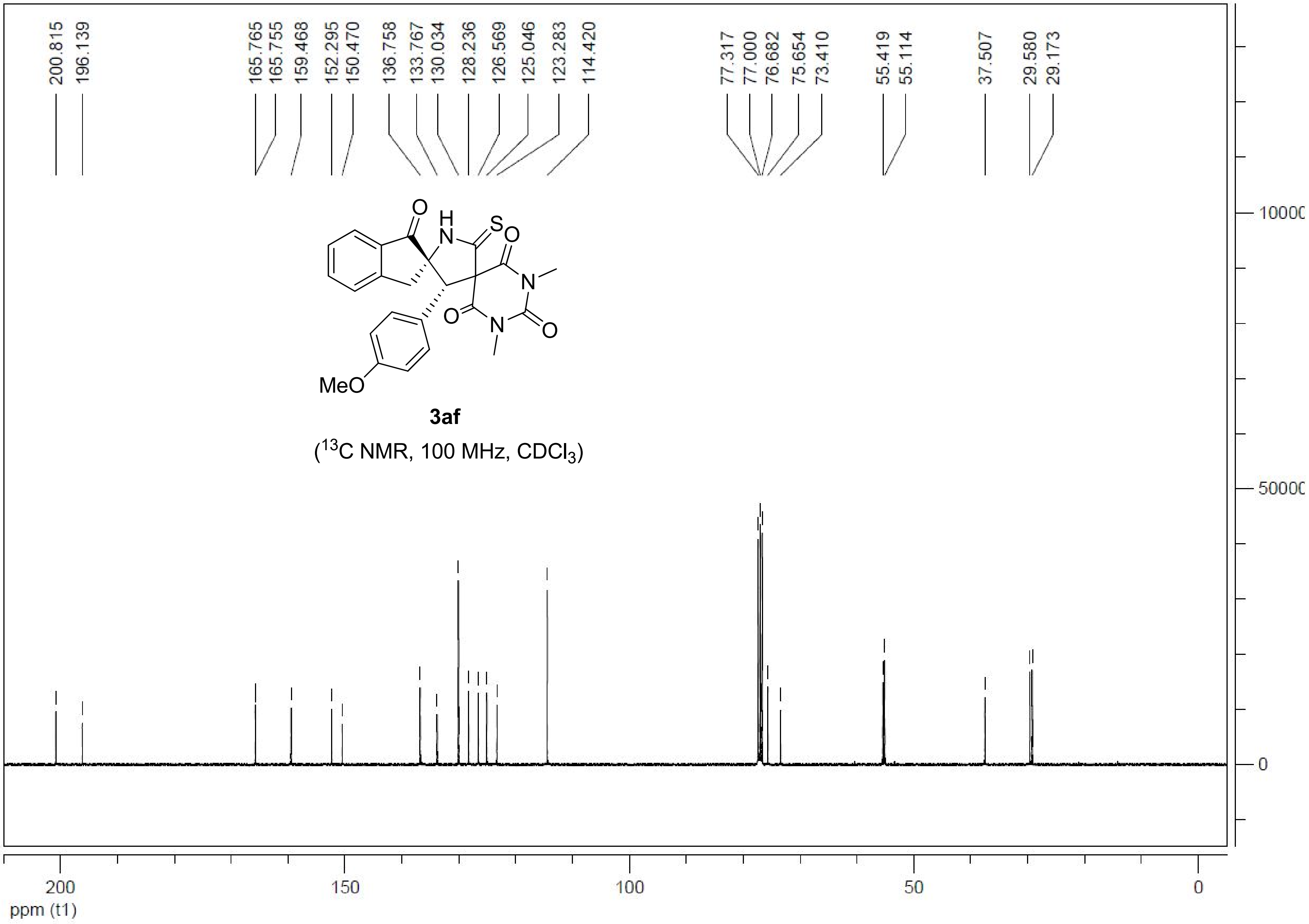
200.815
196.139
165.765
165.755
159.468
152.295
150.470
136.758
133.767
130.034
128.236
126.569
125.046
123.283
114.420
77.317
77.000
76.682
75.654
73.410
55.419
55.114
37.507
29.580
29.173
O
H
N
S
O
N
O
N
O
MeO
3af
(^{13}C NMR, 100 MHz, $CDCl_3$)
200
150
100
50
0
ppm (t1)
0
50000
10000

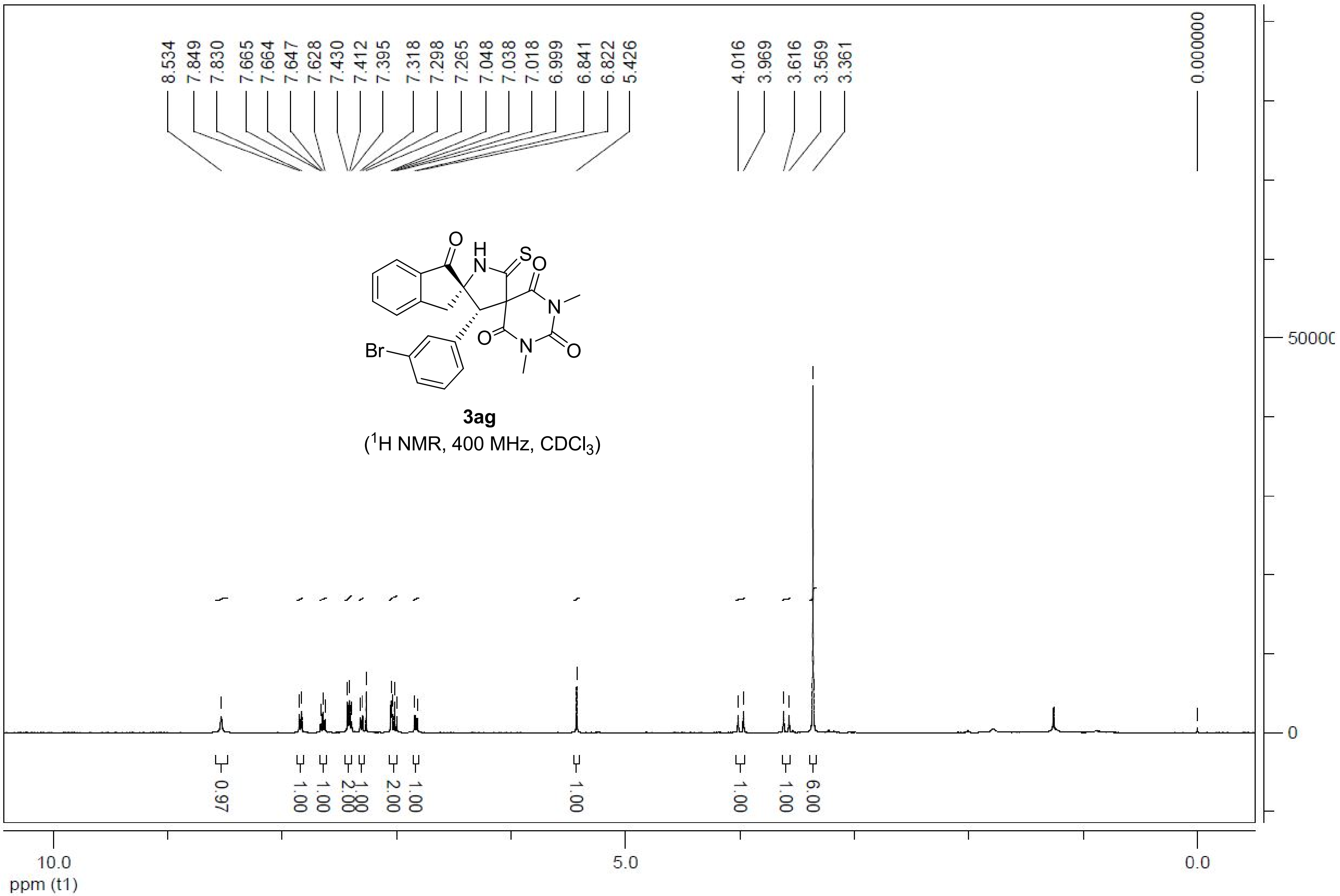
8.534 7.849 7.830 7.665 7.664 7.647 7.628 7.430 7.412 7.395 7.318 7.298 7.265 7.048 7.038 7.018 6.999 6.841 6.822 5.426
4.016 3.969 3.616 3.569 3.361
0.000000
0.97 1.00 1.00 2.00 1.00 2.00 1.00 1.00 1.00 1.00 6.00
50000
0
10.0
5.0
0.0
ppm (t1)
Br
O
H
N
S
O
N
O
N
O
3ag
(^{1}H NMR, 400 MHz, $CDCl_3$)

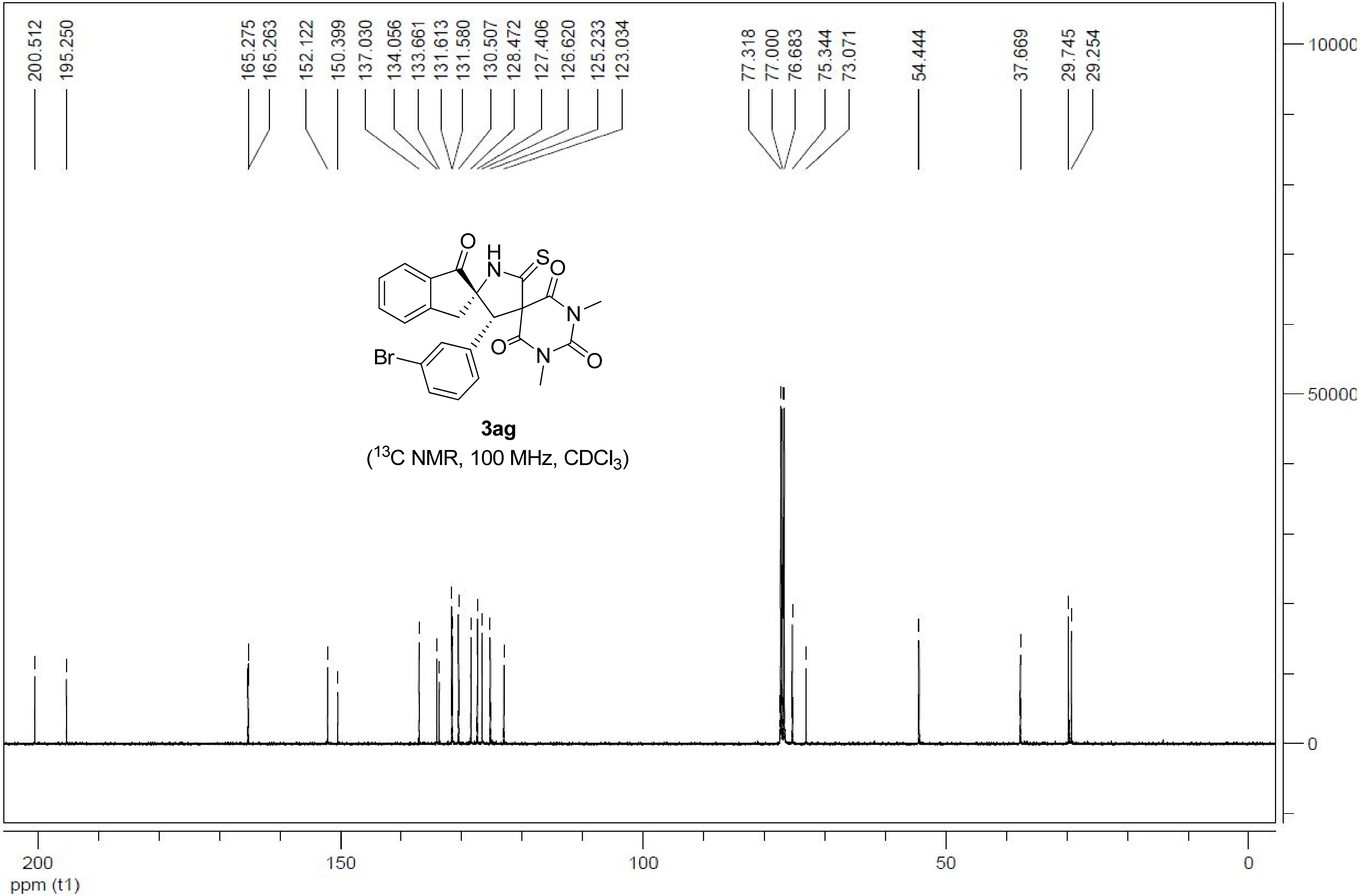
200.512
195.250
165.275
165.263
152.122
150.399
137.030
134.056
133.661
131.613
131.580
130.507
128.472
127.406
126.620
125.233
123.034
77.318
77.000
76.683
75.344
73.071
54.444
37.669
29.745
29.254
O
H
N
S
O
N
Br
O
N
O
3ag
(^{13}C NMR, 100 MHz, CDCl$_3$)
10000
50000
0
200
150
100
50
0
ppm (t1)

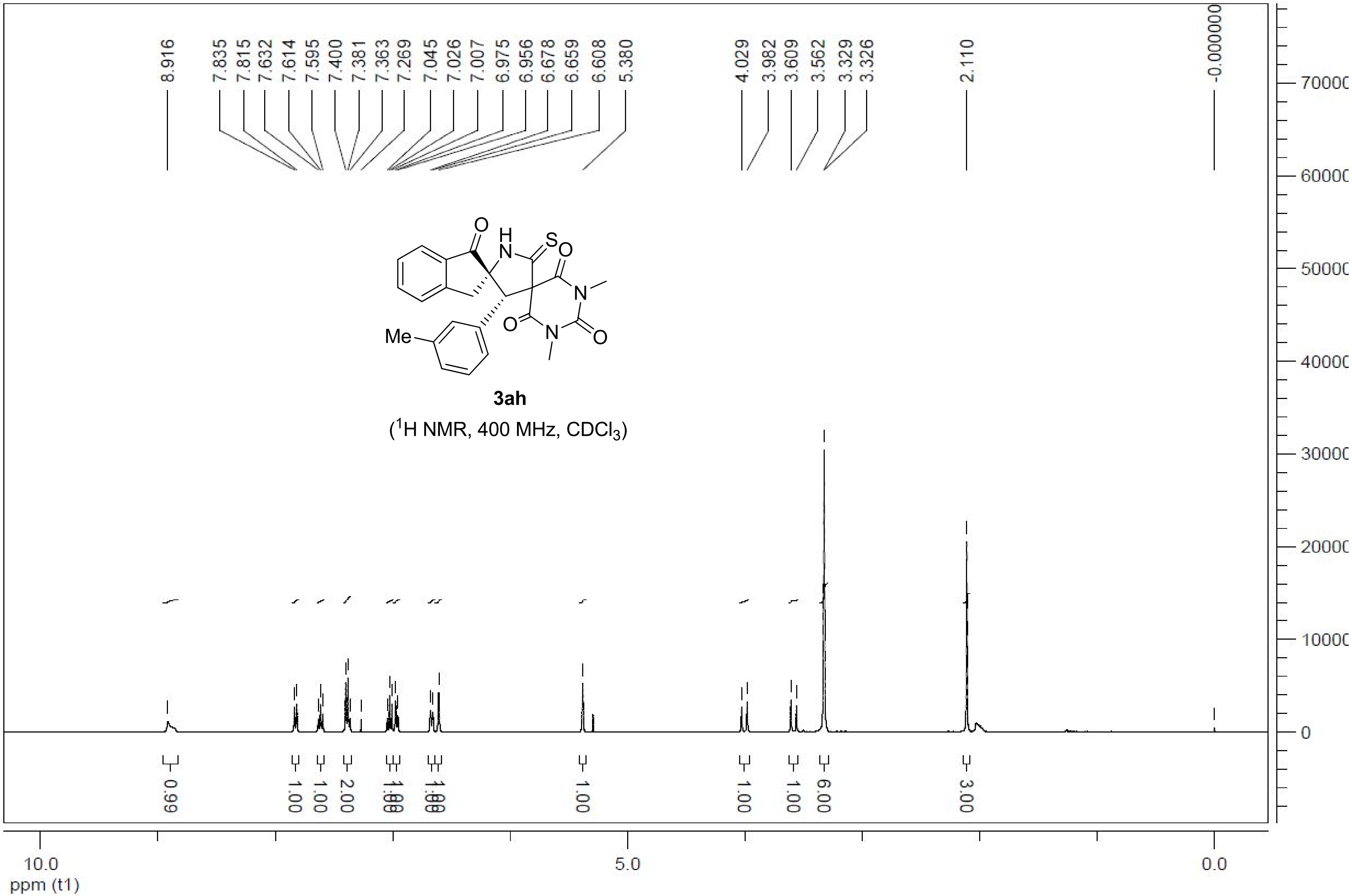

3ah
(^{1}H NMR, 400 MHz, $CDCl_3$)

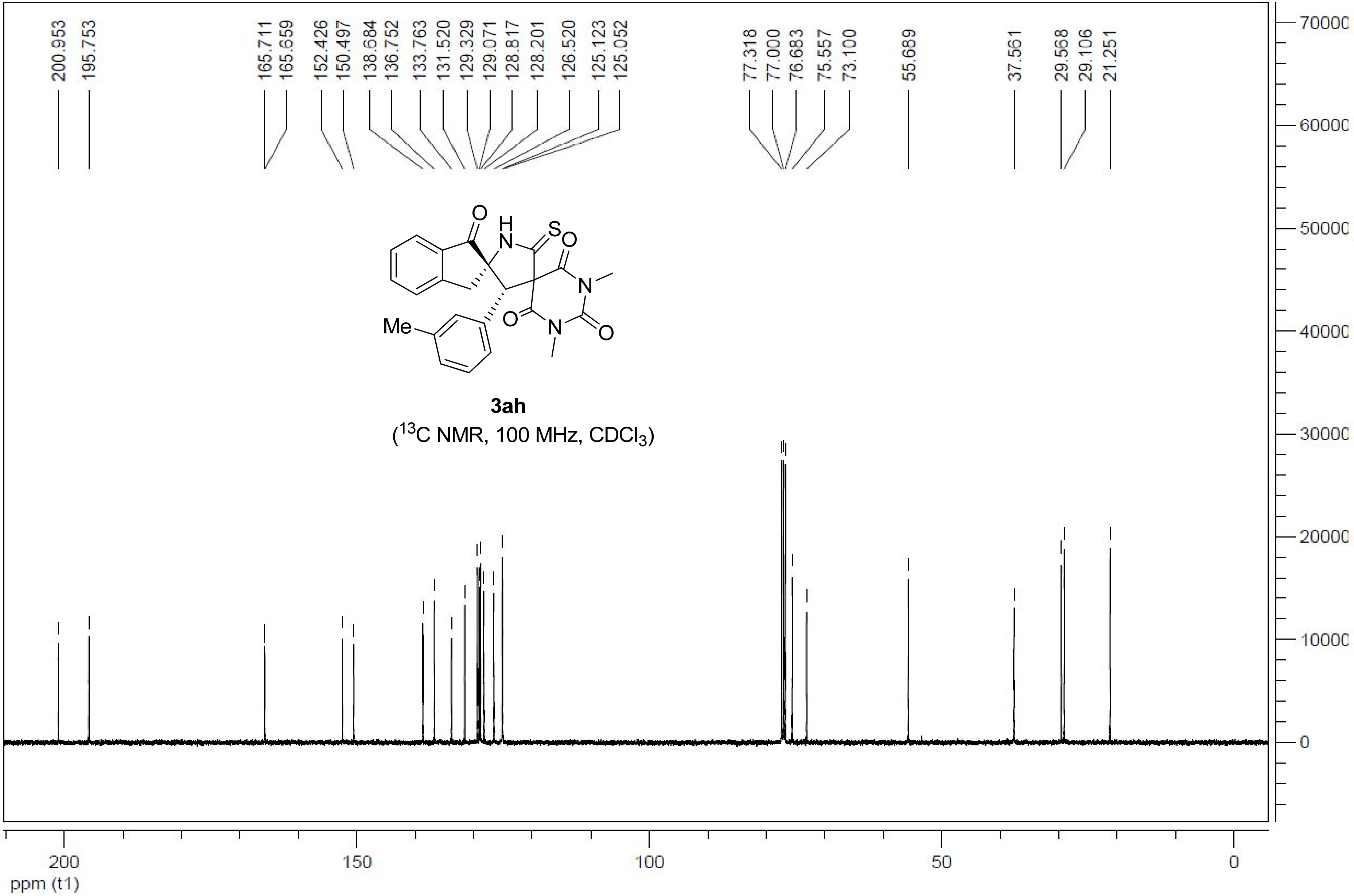

3ah

(^{13}C NMR, 100 MHz, $CDCl_3$)

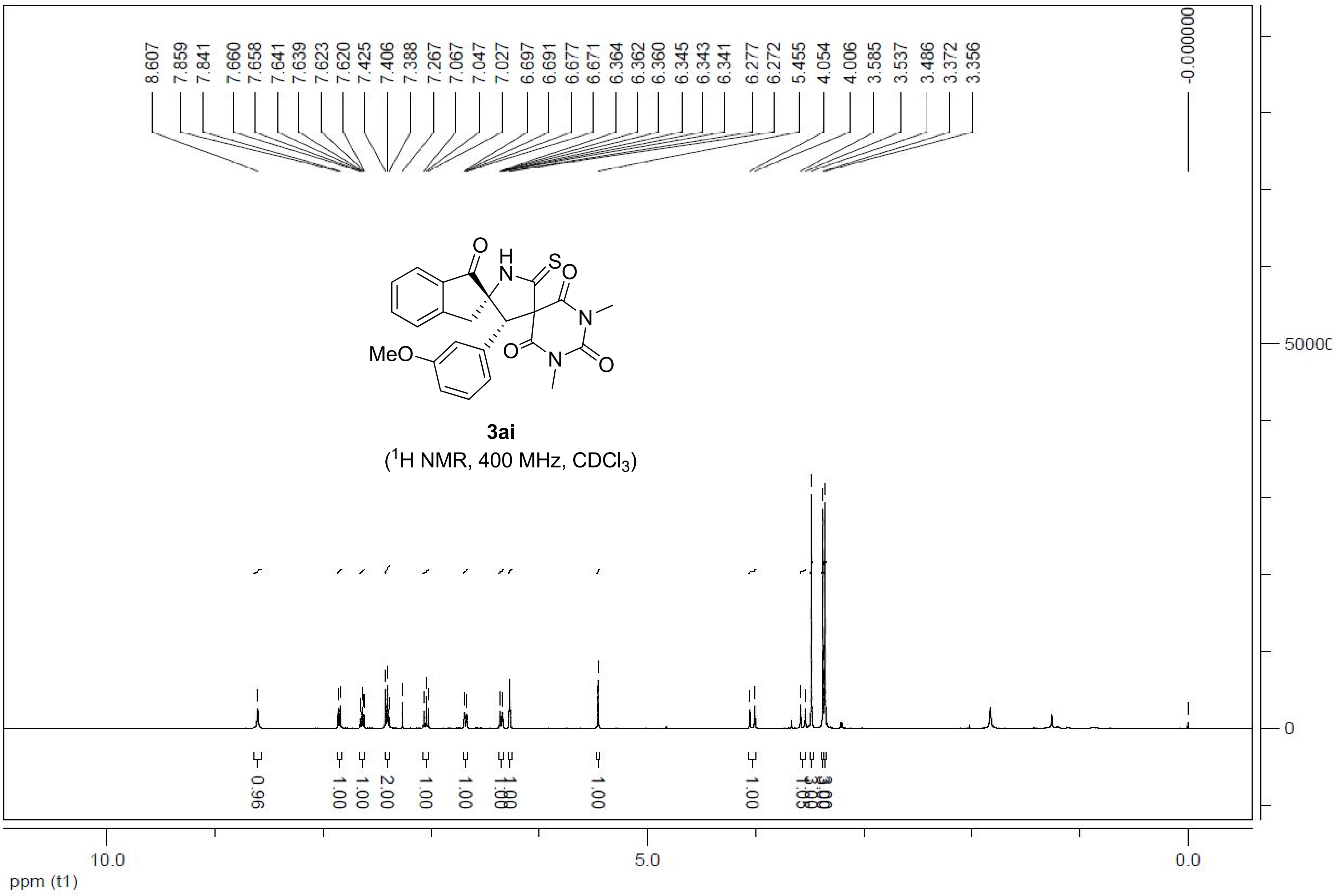
8.607
7.859
7.841
7.660
7.658
7.641
7.639
7.623
7.620
7.425
7.406
7.388
7.267
7.067
7.047
7.027
6.697
6.691
6.677
6.671
6.364
6.362
6.360
6.345
6.343
6.341
6.277
6.272
5.455
4.054
4.006
3.585
3.537
3.486
3.372
3.356
-0.000000
3ai
(¹H NMR, 400 MHz, CDCl3)
50000
0
0.96
1.00
1.00
2.00
1.00
1.00
1.00
1.00
1.00
10.0
5.0
0.0
ppm (t1)

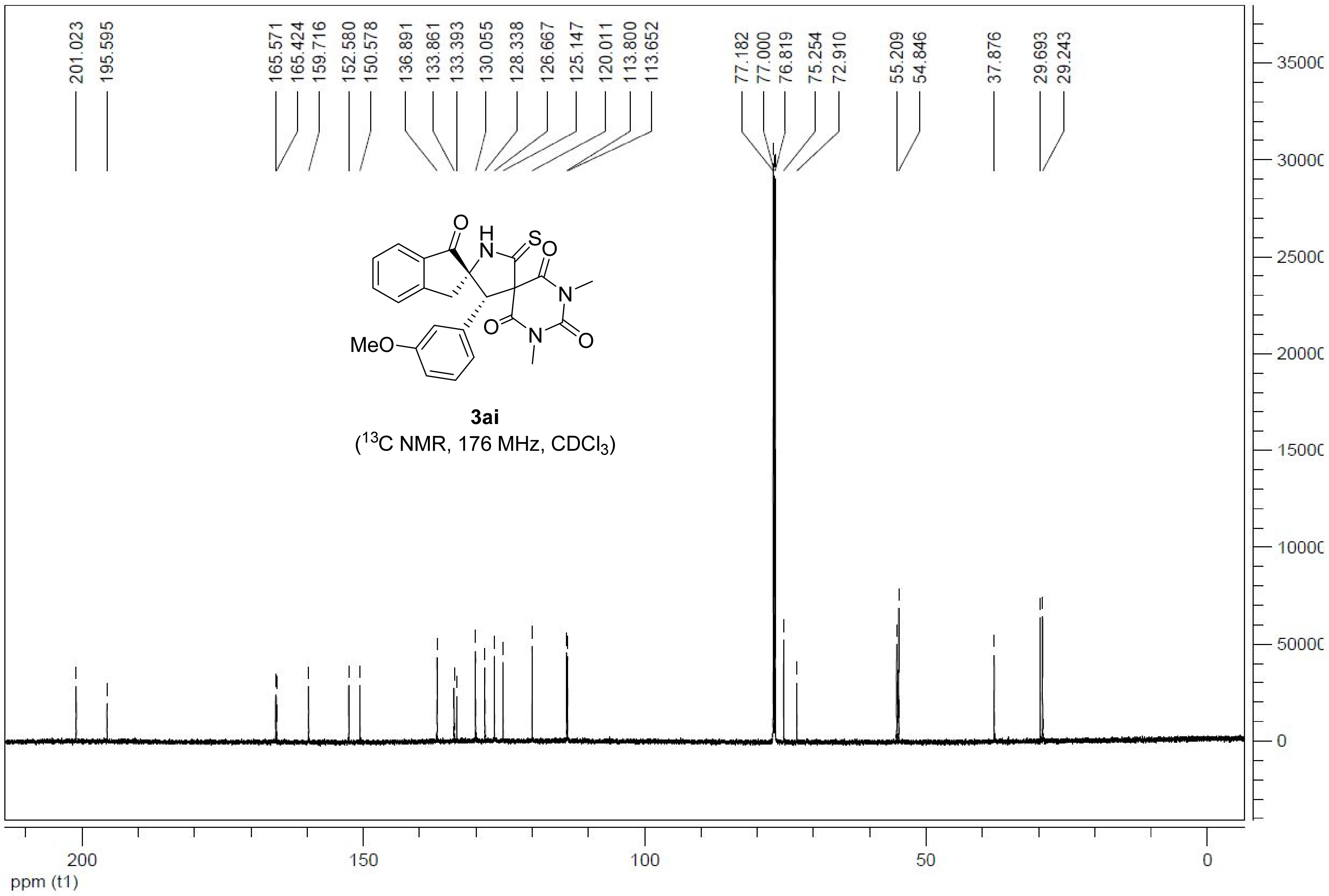
201.023
195.595
165.571
165.424
159.716
152.580
150.578
136.891
133.861
133.393
130.055
128.338
126.667
125.147
120.011
113.800
113.652
77.182
77.000
76.819
75.254
72.910
55.209
54.846
37.876
29.693
29.243
3ai
(^{13}C NMR, 176 MHz, CDCl$_3$)
ppm (t1)

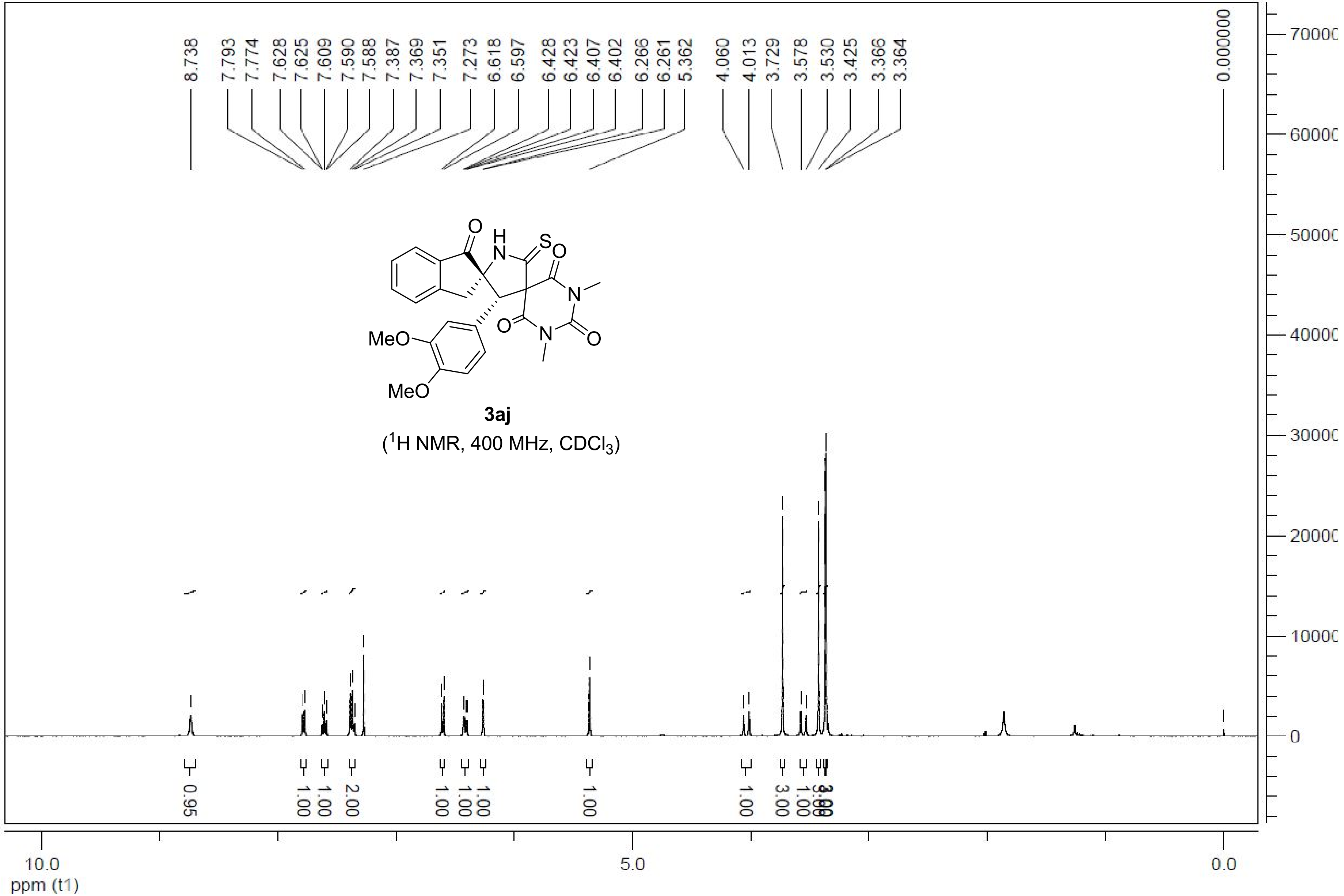
8.738 7.793 7.774 7.628 7.625 7.609 7.590 7.588 7.387 7.369 7.351 7.273 6.618 6.597 6.428 6.423 6.407 6.402 6.266 6.261 5.362 4.060 4.013 3.729 3.578 3.530 3.425 3.366 3.364 0.000000
MeO
MeO
3aj
(^{1}H NMR, 400 MHz, $CDCl_3$)
0.95 1.00 1.00 2.00 1.00 1.00 1.00 1.00 1.00 3.00 1.00
10.0
5.0
0.0
ppm (t1)
70000
60000
50000
40000
30000
20000
10000
0

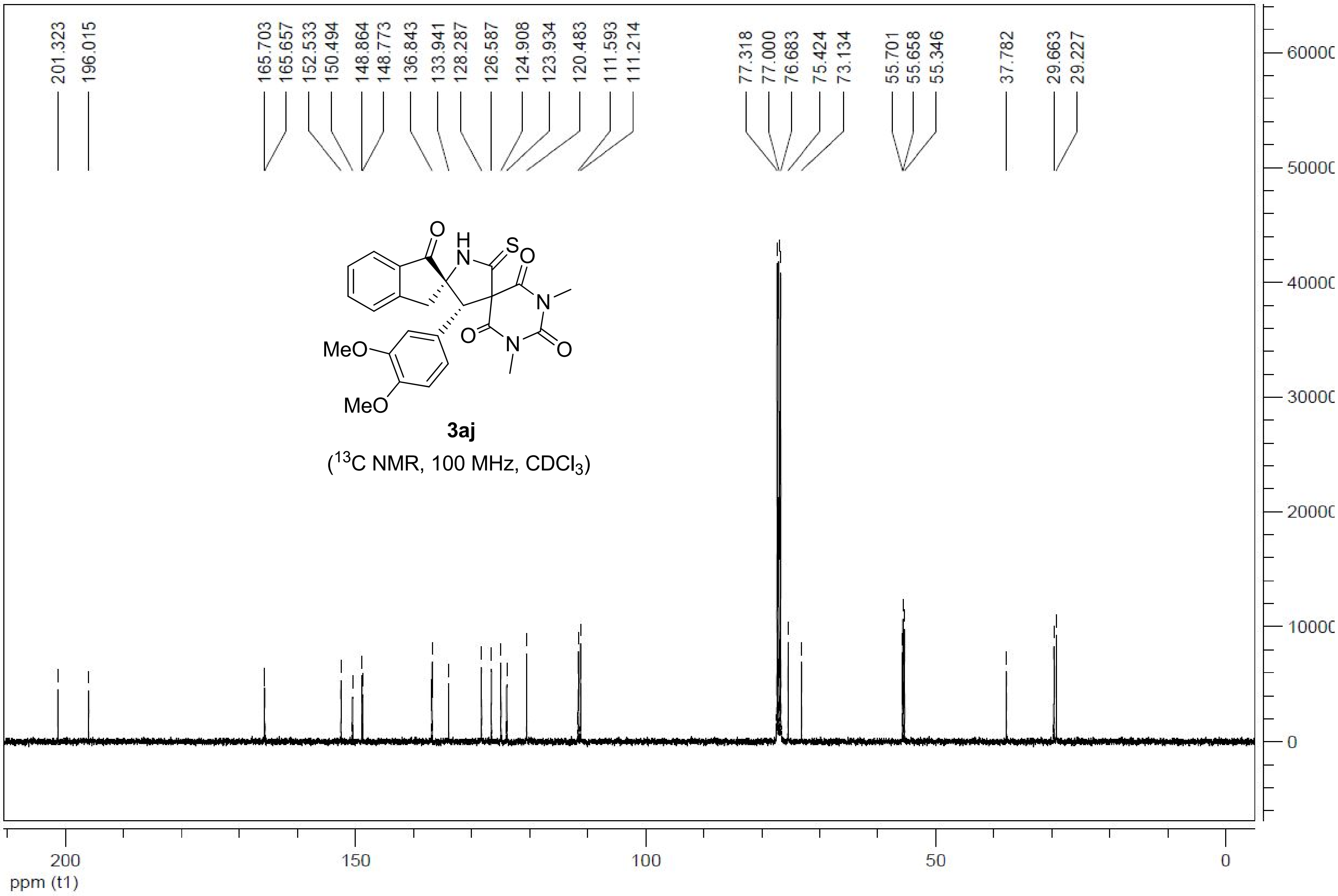
201.323
196.015
165.703
165.657
152.533
150.494
148.864
148.773
136.843
133.941
128.287
126.587
124.908
123.934
120.483
111.593
111.214
77.318
77.000
76.683
75.424
73.134
55.701
55.658
55.346
37.782
29.663
29.227
MeO
MeO
3aj
(^{13}C NMR, 100 MHz, $CDCl_3$)
ppm (t1)

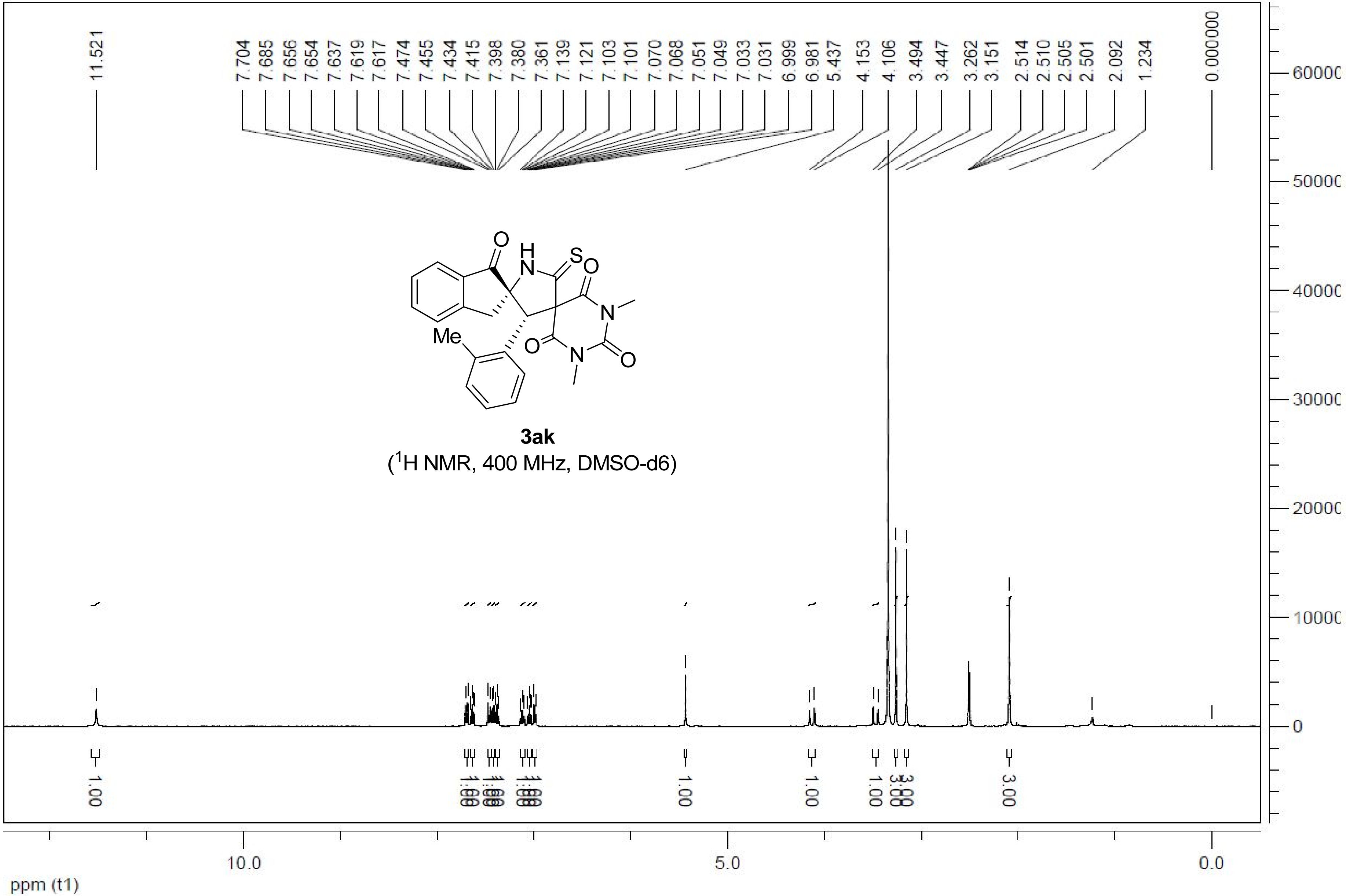

11.521
7.704
7.685
7.656
7.654
7.637
7.619
7.617
7.474
7.455
7.434
7.415
7.398
7.380
7.361
7.139
7.121
7.103
7.101
7.070
7.068
7.051
7.049
7.033
7.031
6.999
6.981
5.437
4.153
4.106
3.494
3.447
3.262
3.151
2.514
2.510
2.505
2.501
2.092
1.234
0.000000
Me
3ak
(^{1}H NMR, 400 MHz, DMSO-d6)
1.00
1.00
1.00
1.00
3.00
3.00
60000
50000
40000
30000
20000
10000
0
10.0
5.0
0.0
ppm (t1)

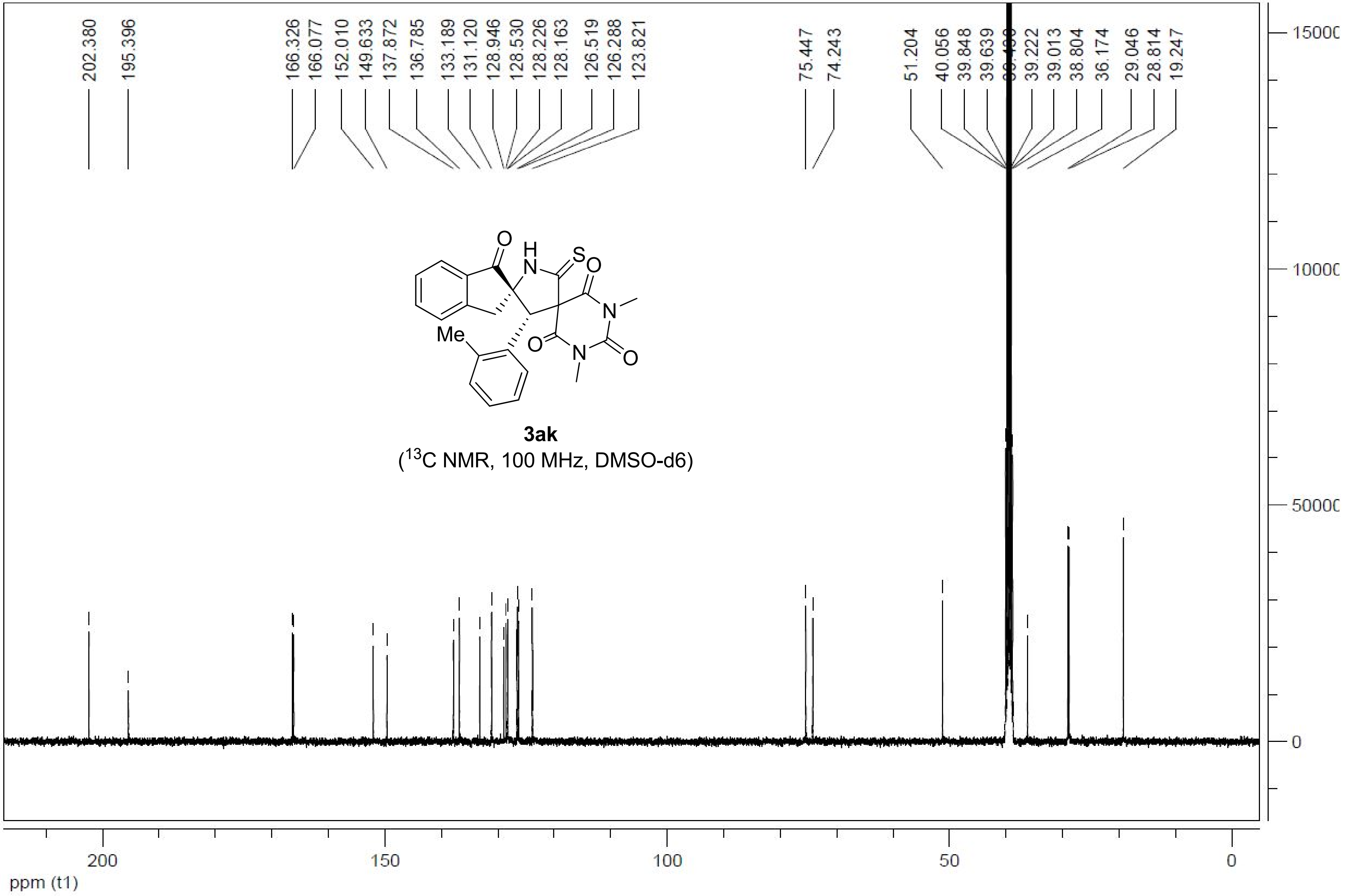
202.380
195.396
166.326
166.077
152.010
149.633
137.872
136.785
133.189
131.120
128.946
128.530
128.226
128.163
126.519
126.288
123.821
75.447
74.243
51.204
40.056
39.848
39.639
39.222
39.013
38.804
36.174
29.046
28.814
19.247
3ak
(^{13}C NMR, 100 MHz, DMSO-d6)
ppm (t1)

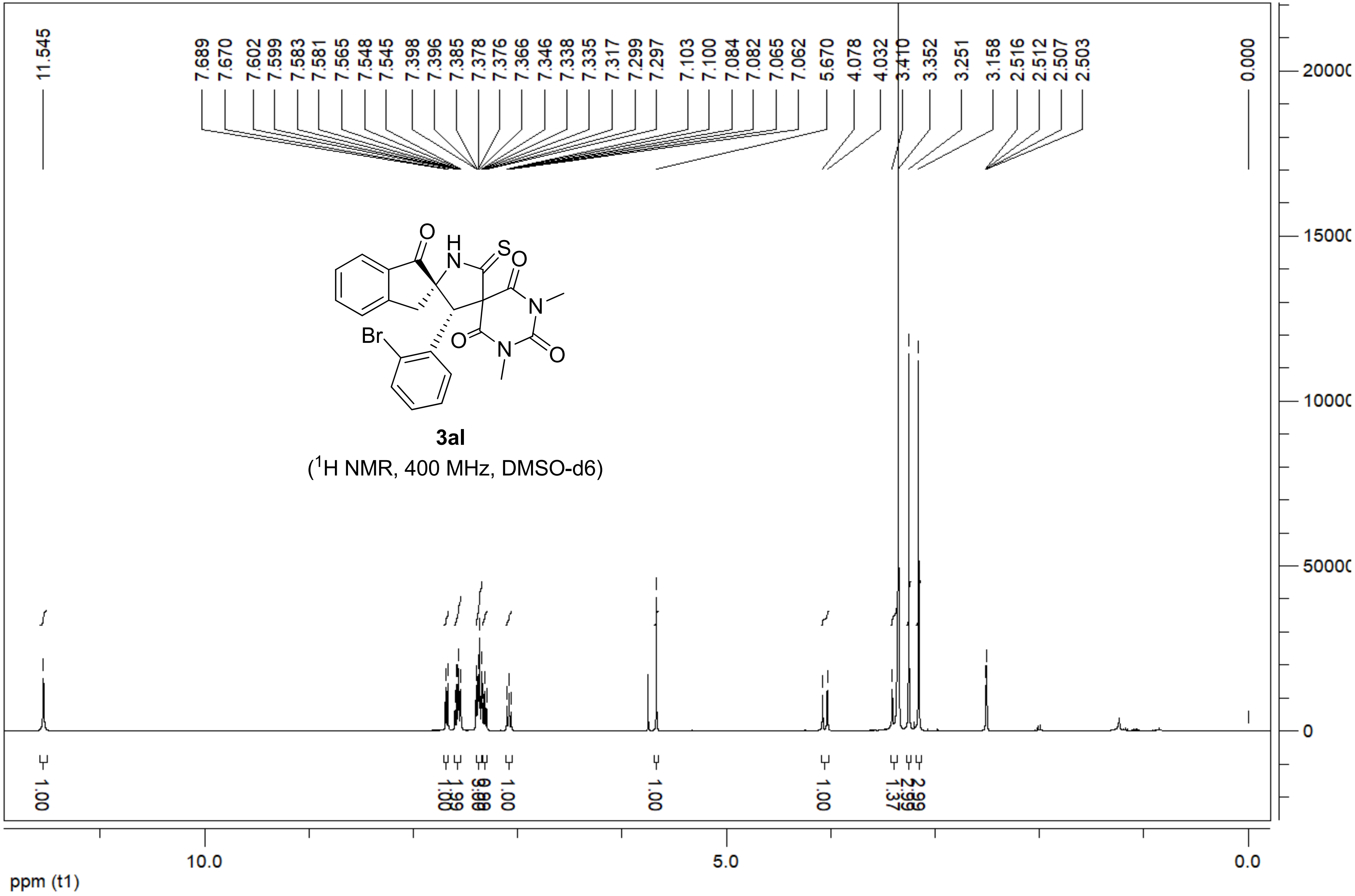

3al

(^{1}H NMR, 400 MHz, DMSO-d6)

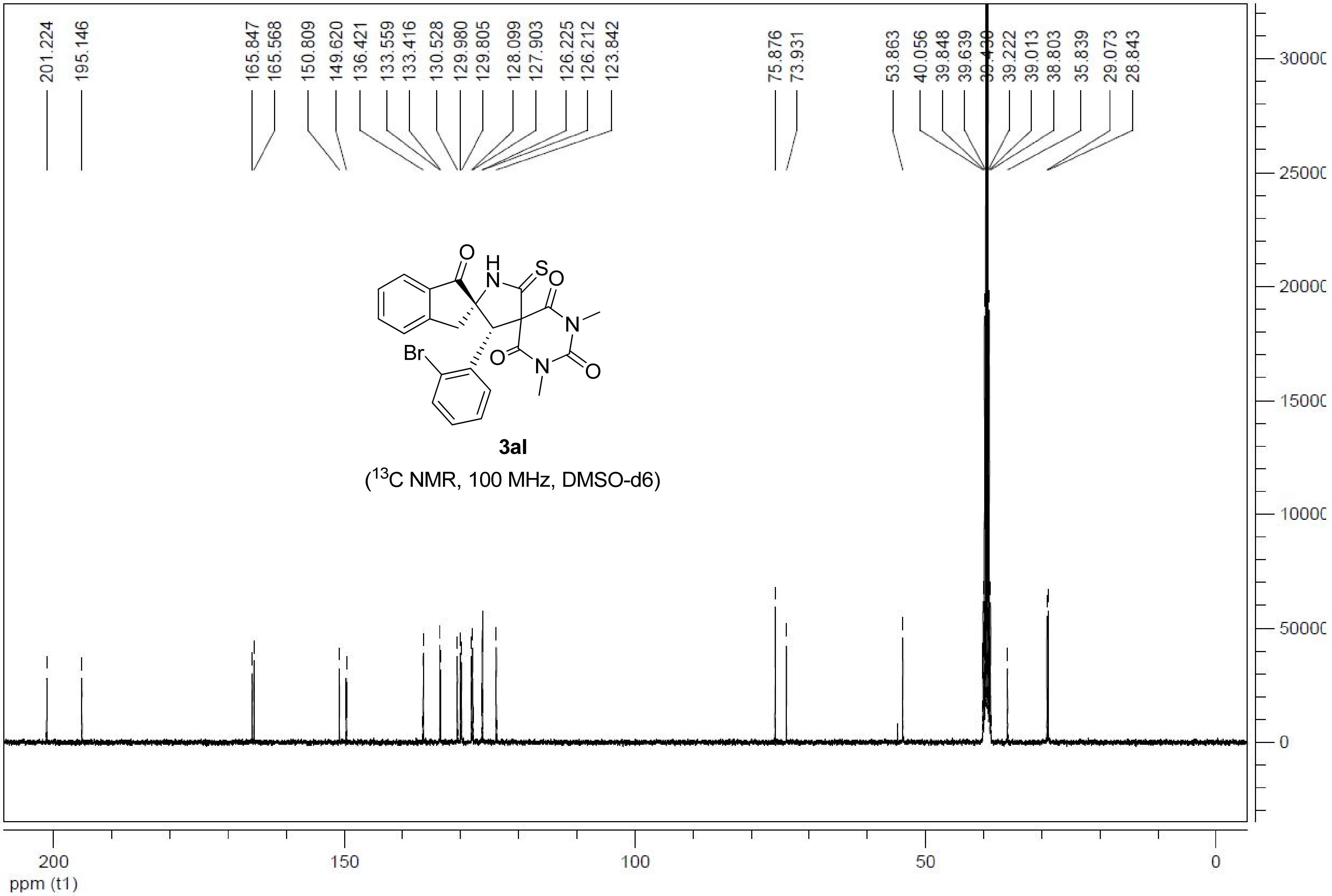
201.224
195.146
165.847
165.568
150.809
149.620
136.421
133.559
133.416
130.528
129.980
129.805
128.099
127.903
126.225
126.212
123.842
75.876
73.931
53.863
40.056
39.848
39.639
39.222
39.013
38.803
35.839
29.073
28.843
3al
(^{13}C NMR, 100 MHz, DMSO-d6)
200
150
100
50
0
ppm (t1)
30000
25000
20000
15000
10000
50000
0

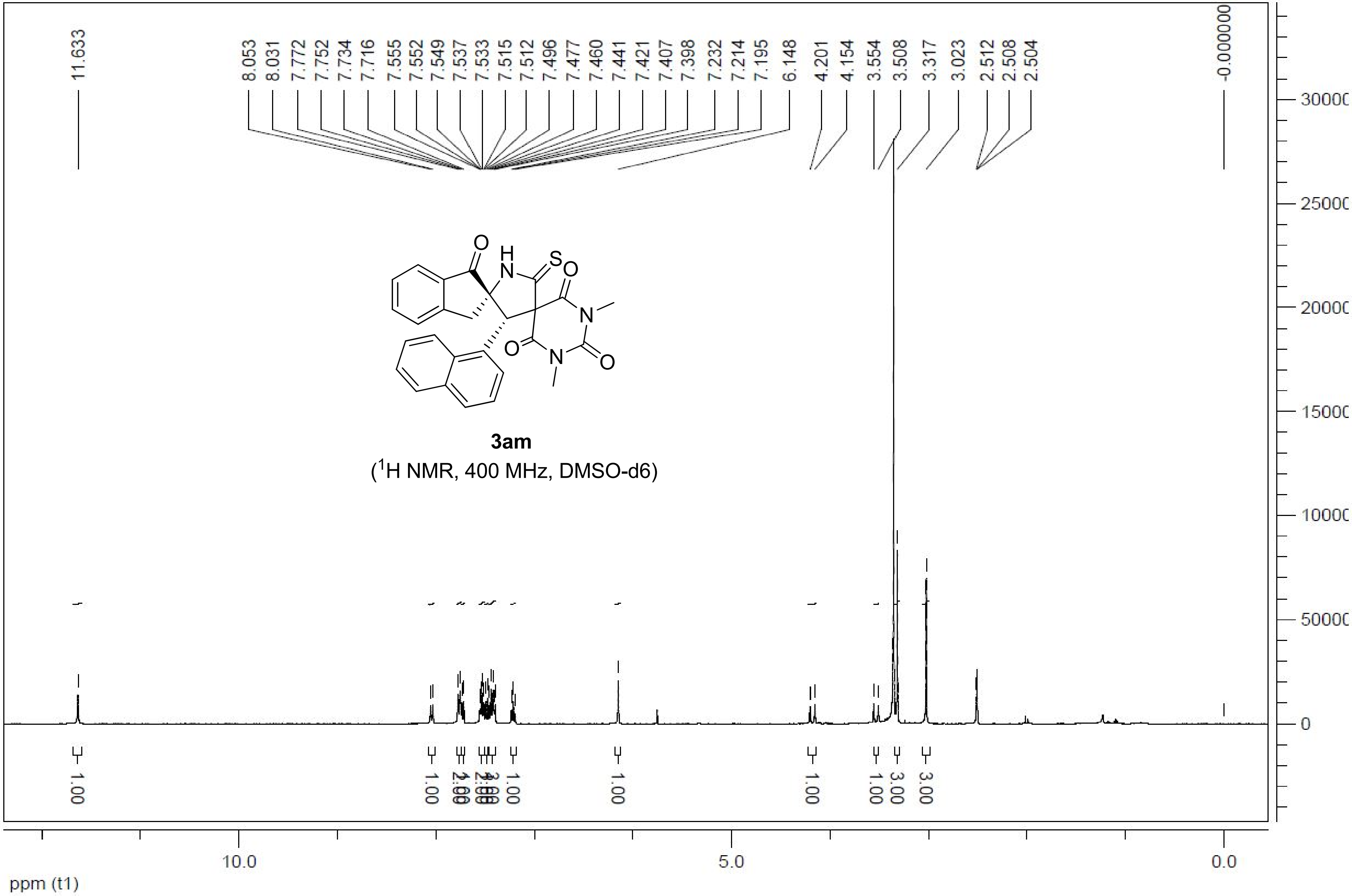

3am

(^{1}H NMR, 400 MHz, DMSO-d6)

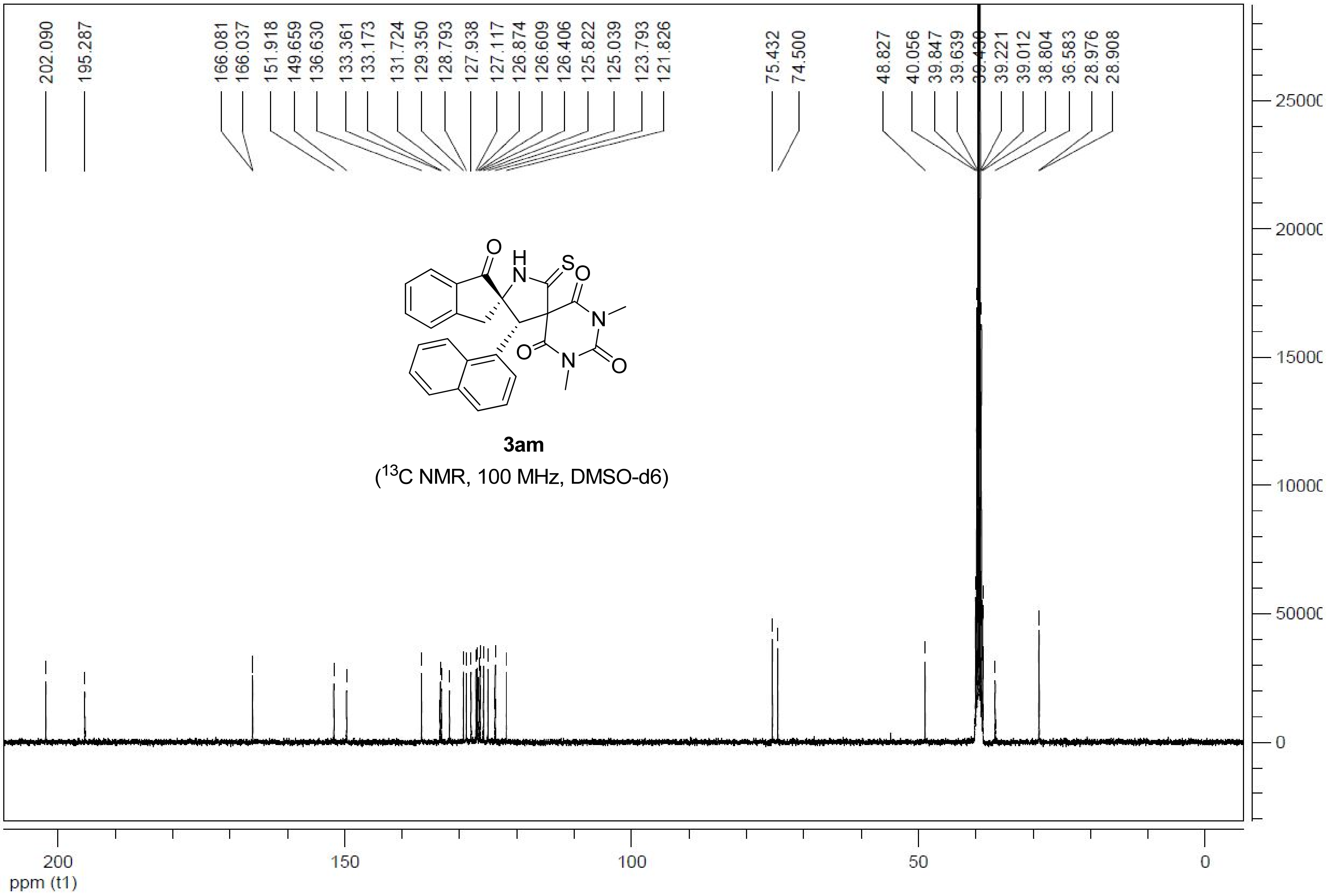

3am

(^{13}C NMR, 100 MHz, DMSO-d6)

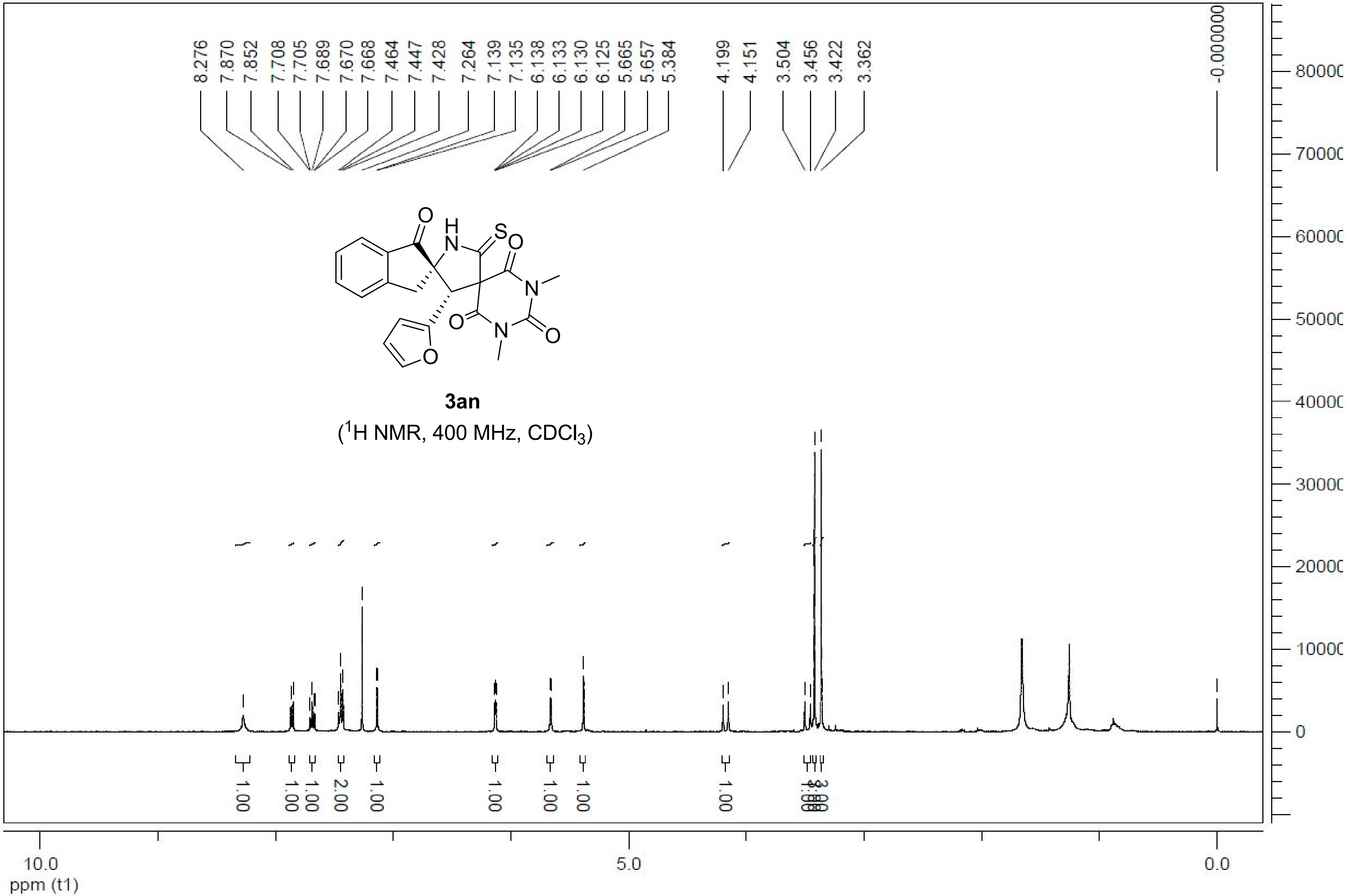

3an

(^{1}H NMR, 400 MHz, $CDCl_3$)

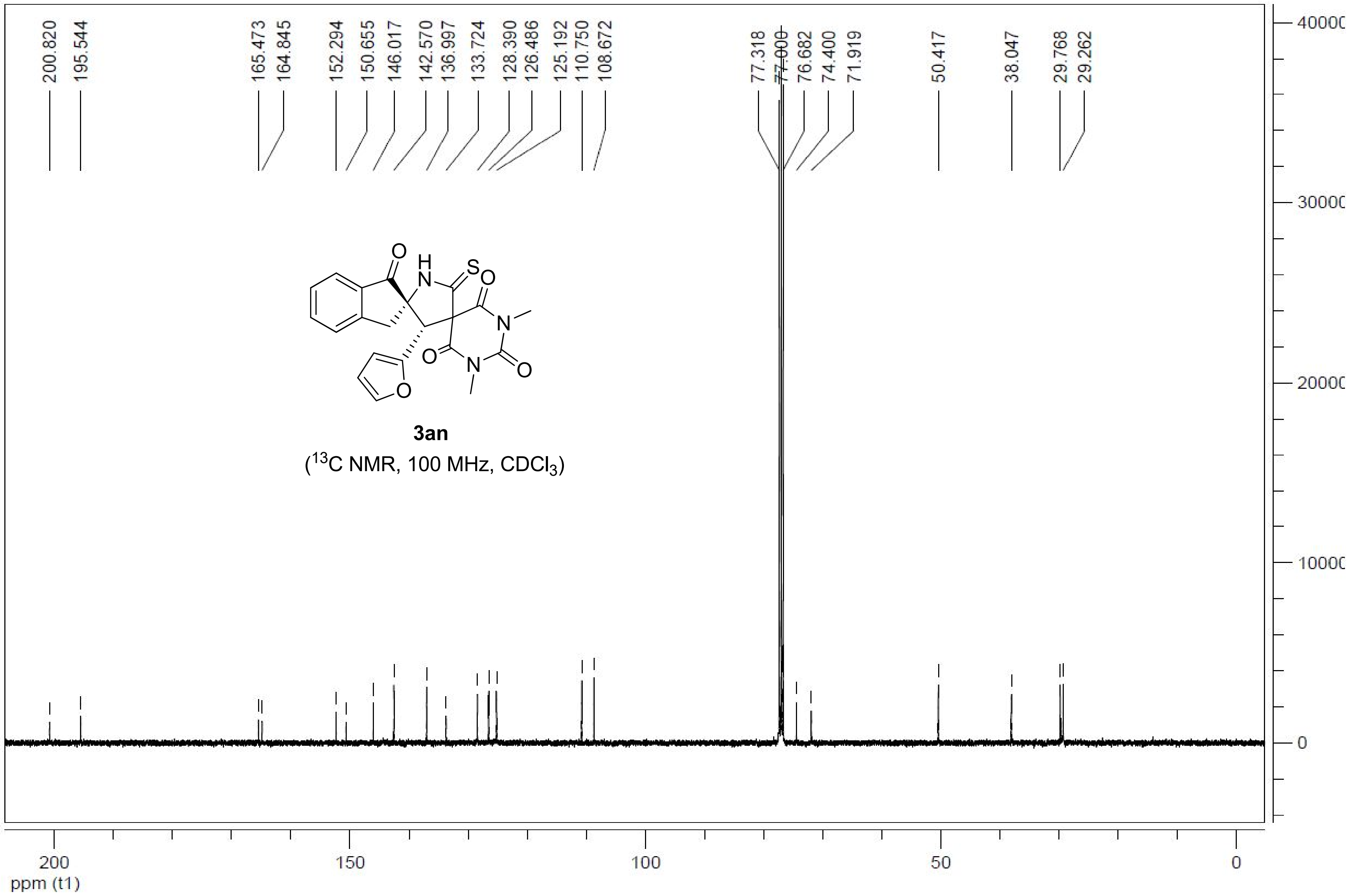
200.820
195.544
165.473
164.845
152.294
150.655
146.017
142.570
136.997
133.724
128.390
126.486
125.192
110.750
108.672
77.318
77.000
76.682
74.400
71.919
50.417
38.047
29.768
29.262
3an
(13C NMR, 100 MHz, CDCl3)
40000
30000
20000
10000
0
200
150
100
50
0
ppm (t1)

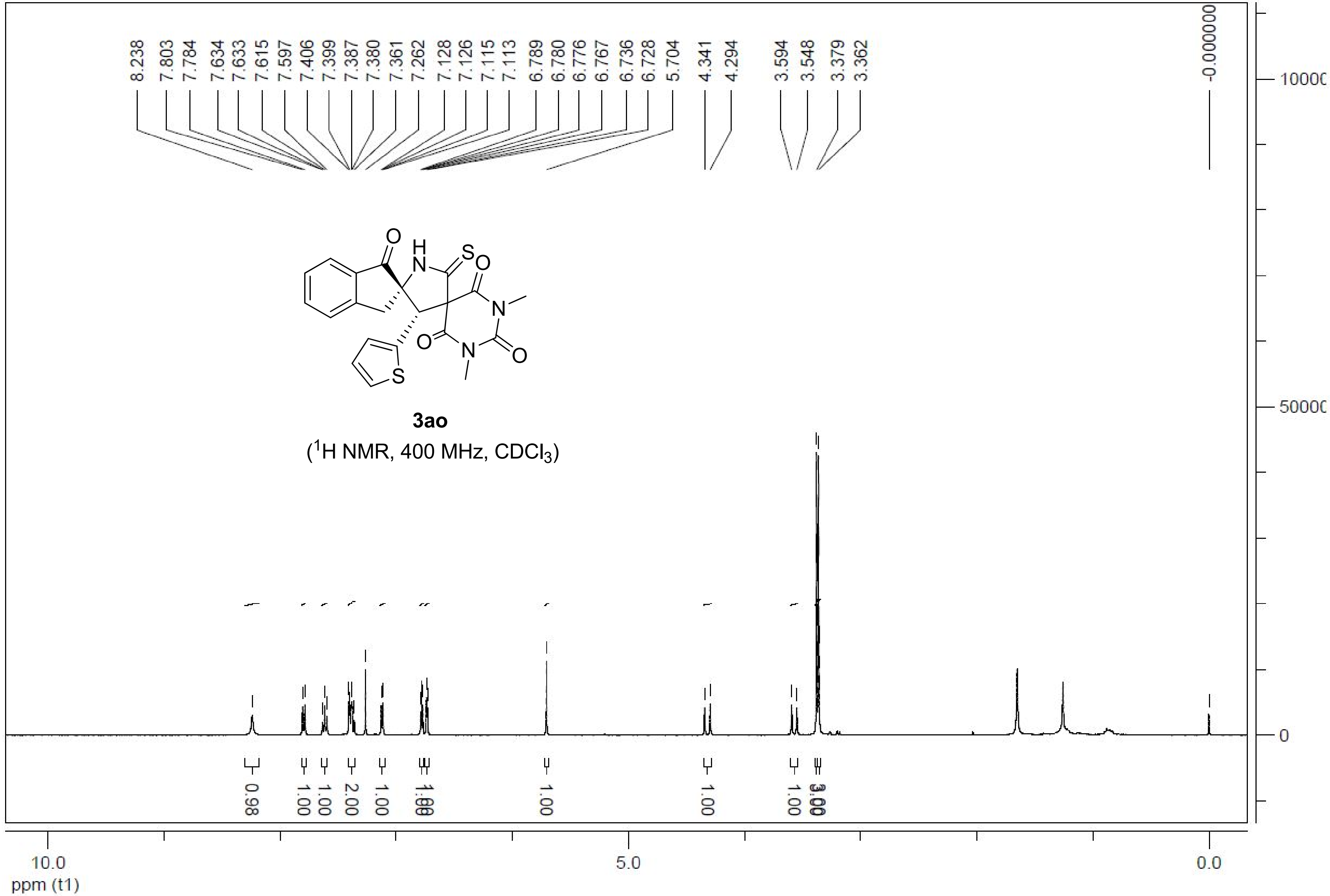

3ao

(^{1}H NMR, 400 MHz, $CDCl_3$)

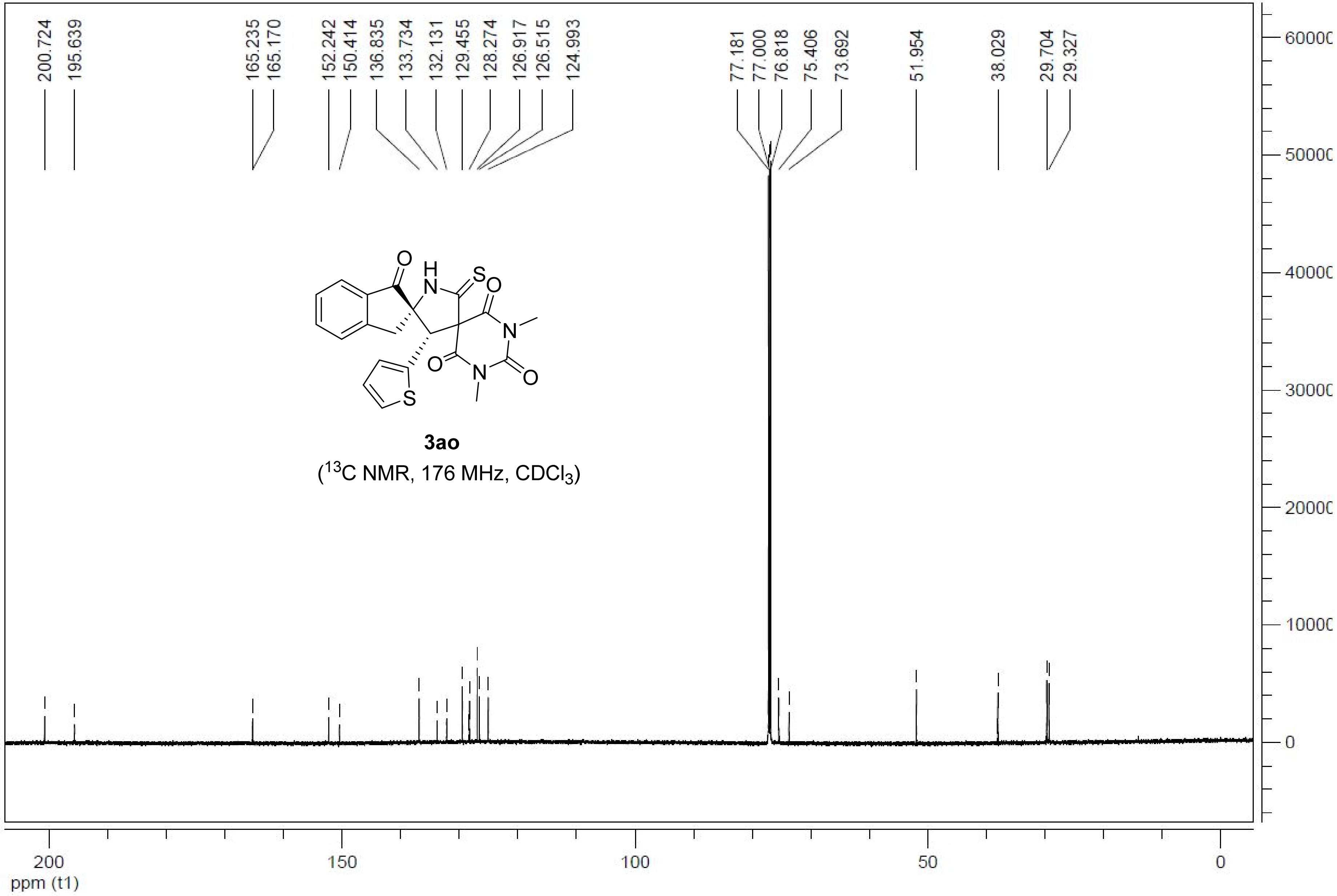

3ao
(^{13}C NMR, 176 MHz, $CDCl_3$)

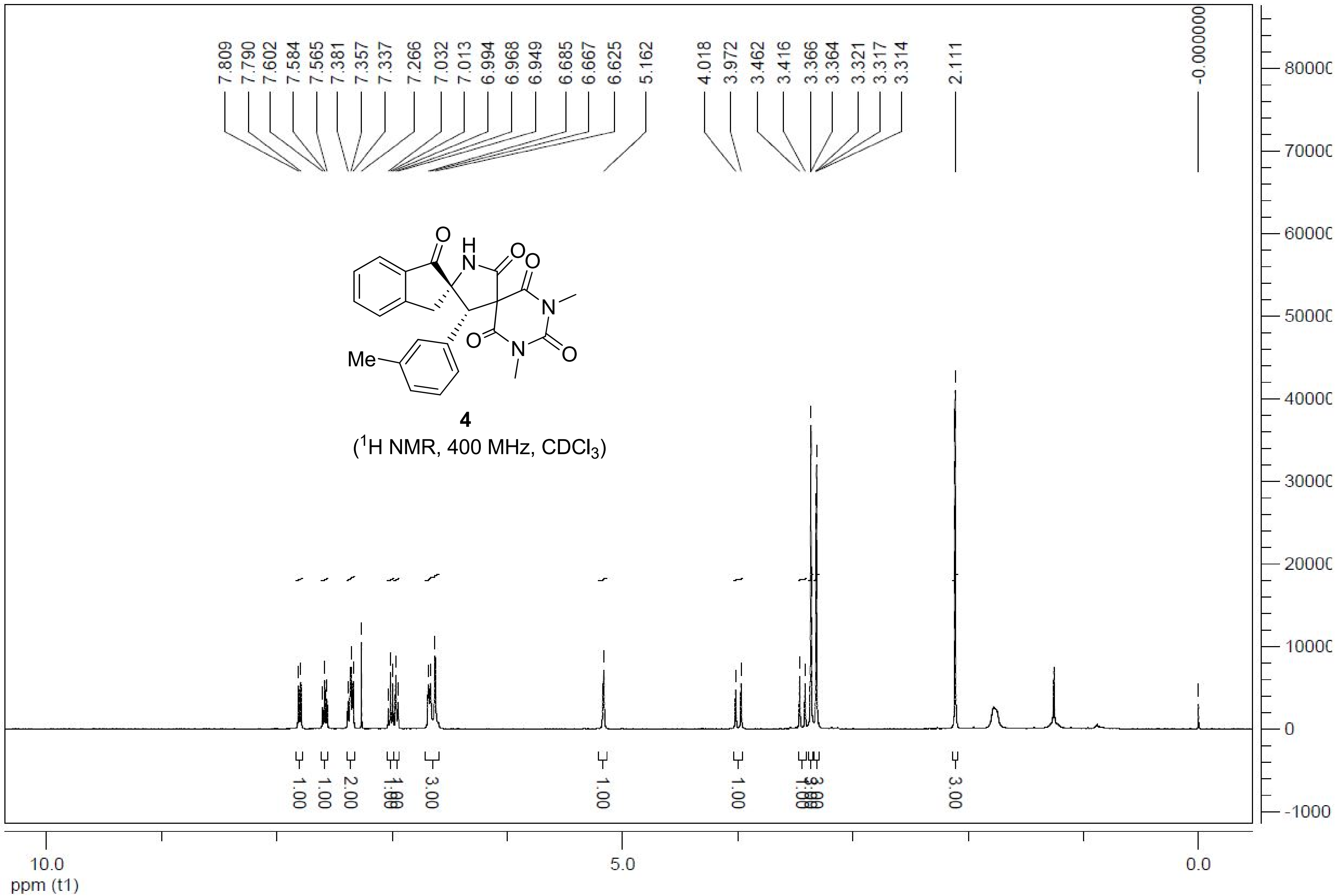
7.809
7.790
7.602
7.584
7.565
7.381
7.357
7.337
7.266
7.032
7.013
6.994
6.968
6.949
6.685
6.667
6.625
5.162
4.018
3.972
3.462
3.416
3.366
3.364
3.321
3.317
3.314
2.111
-0.000000
1.00
1.00
2.00
3.00
1.00
1.00
3.00
10.0
5.0
0.0
ppm (t1)
80000
70000
60000
50000
40000
30000
20000
10000
0
-1000
O
H
N
O
O
N
O
N
O
Me
4
(^{1}H NMR, 400 MHz, $CDCl_3$)

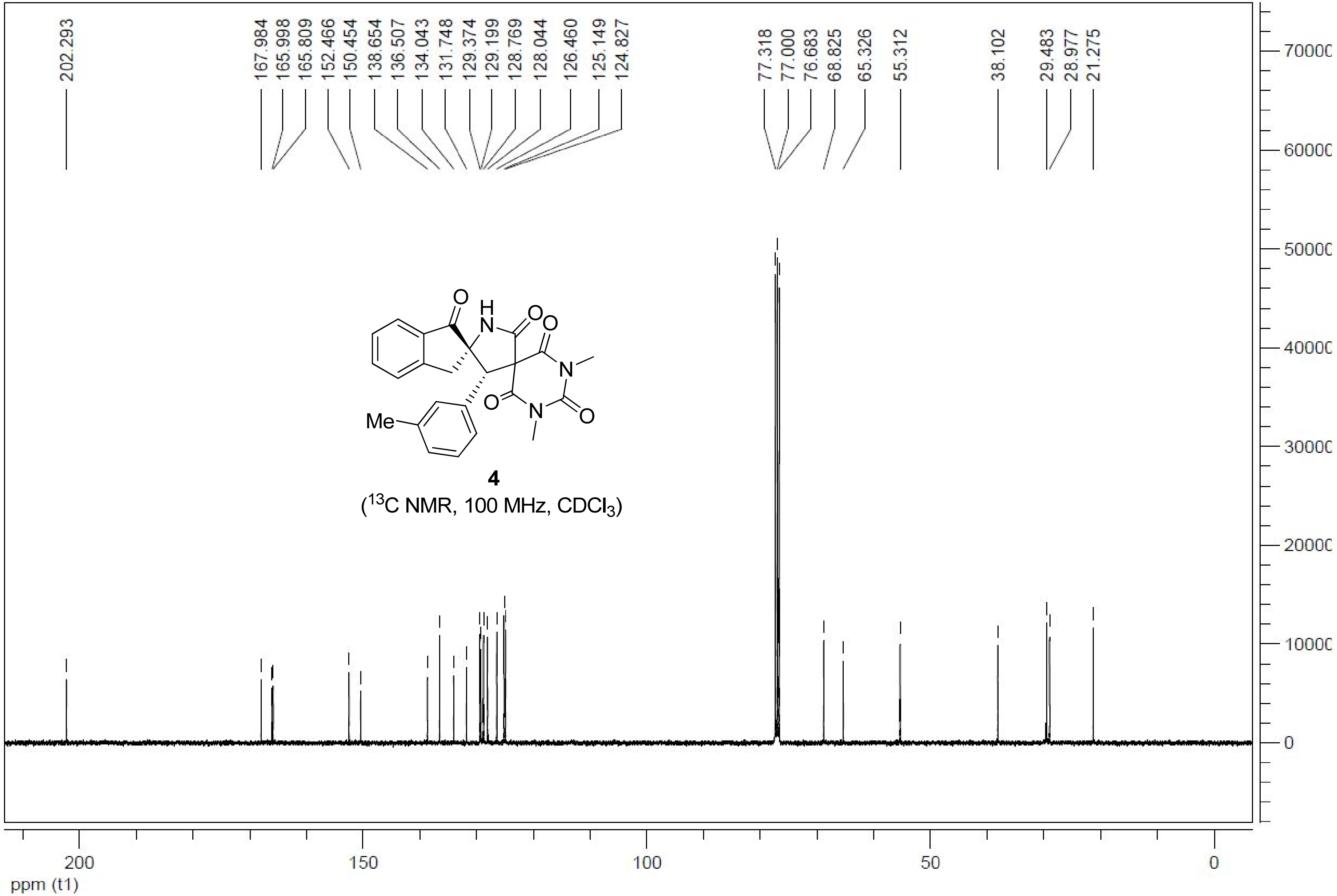
202.293
167.984
165.998
165.809
152.466
150.454
138.654
136.507
134.043
131.748
129.374
129.199
128.769
128.044
126.460
125.149
124.827
77.318
77.000
76.683
68.825
65.326
55.312
38.102
29.483
28.977
21.275
70000
60000
50000
40000
30000
20000
10000
0
200
150
100
50
0
ppm (t1)
Me
O
H
N
N
4
(^{13}C NMR, 100 MHz, $CDCl_3$)

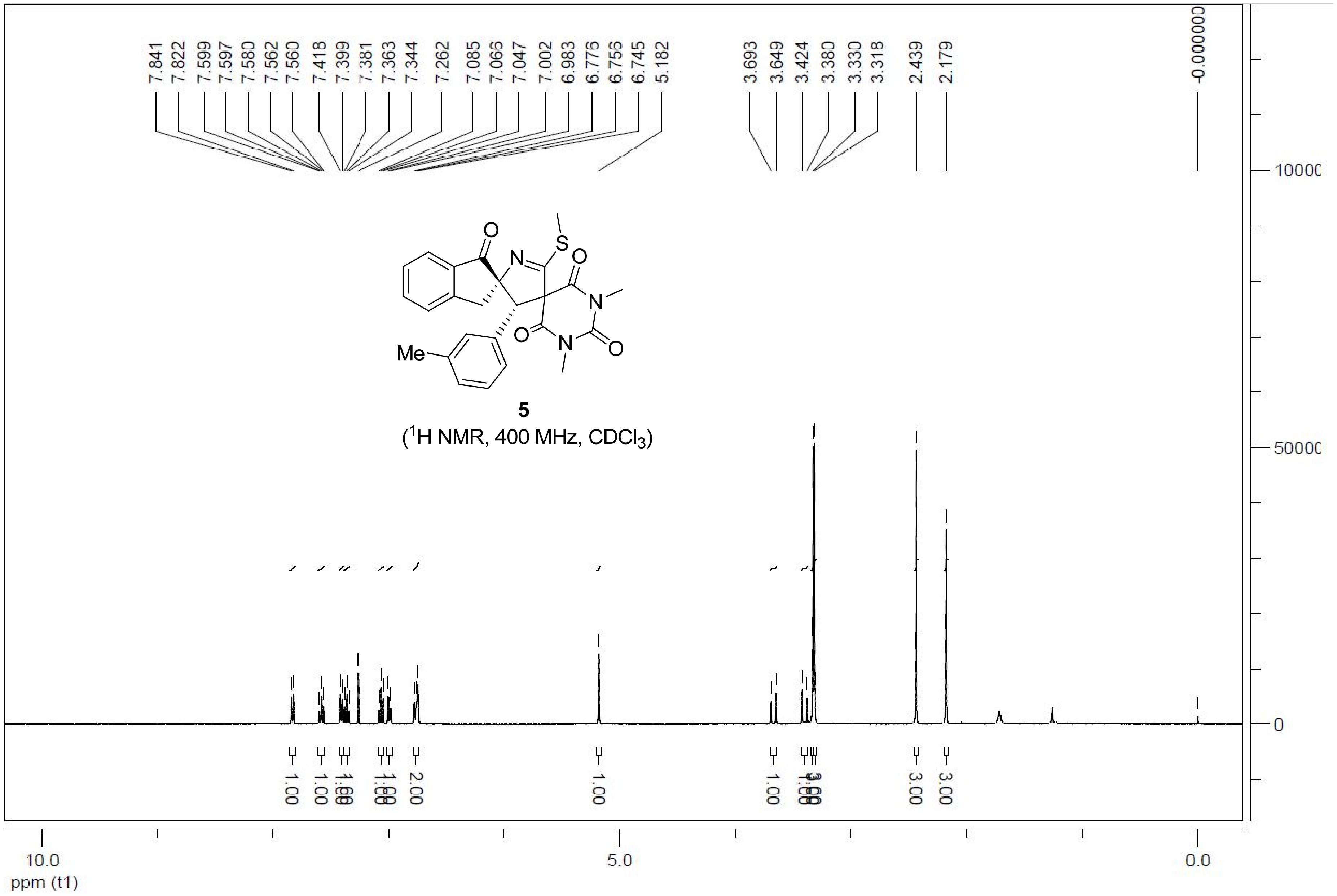
7.841
7.822
7.599
7.597
7.580
7.562
7.560
7.418
7.399
7.381
7.363
7.344
7.262
7.085
7.066
7.047
7.002
6.983
6.776
6.756
6.745
5.182
3.693
3.649
3.424
3.380
3.330
3.318
2.439
2.179
-0.000000
O
N
S
O
N
Me
O
N
O
5
(^{1}H NMR, 400 MHz, $CDCl_3$)
1.00
1.00
2.00
1.00
1.00
3.00
3.00
10000
50000
0
10.0
5.0
0.0
ppm (t1)

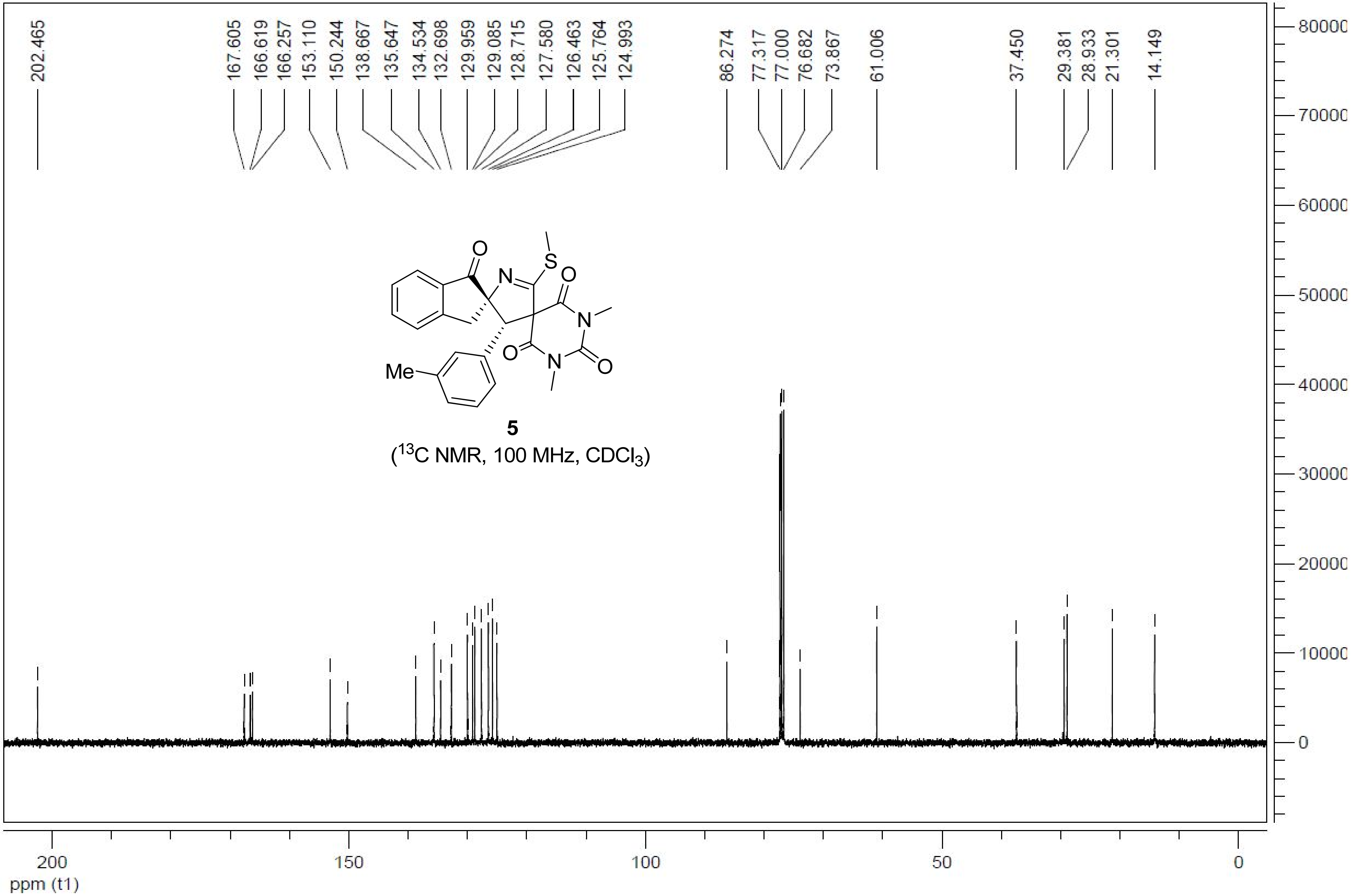
202.465
167.605
166.619
166.257
153.110
150.244
138.667
135.647
134.534
132.698
129.959
129.085
128.715
127.580
126.463
125.764
124.993
86.274
77.317
77.000
76.682
73.867
61.006
37.450
29.381
28.933
21.301
14.149
5
(^{13}C NMR, 100 MHz, $CDCl_3$)
200
150
100
50
0
ppm (t1)
80000
70000
60000
50000
40000
30000
20000
10000
0

5. Copies of HPLC chromatograms

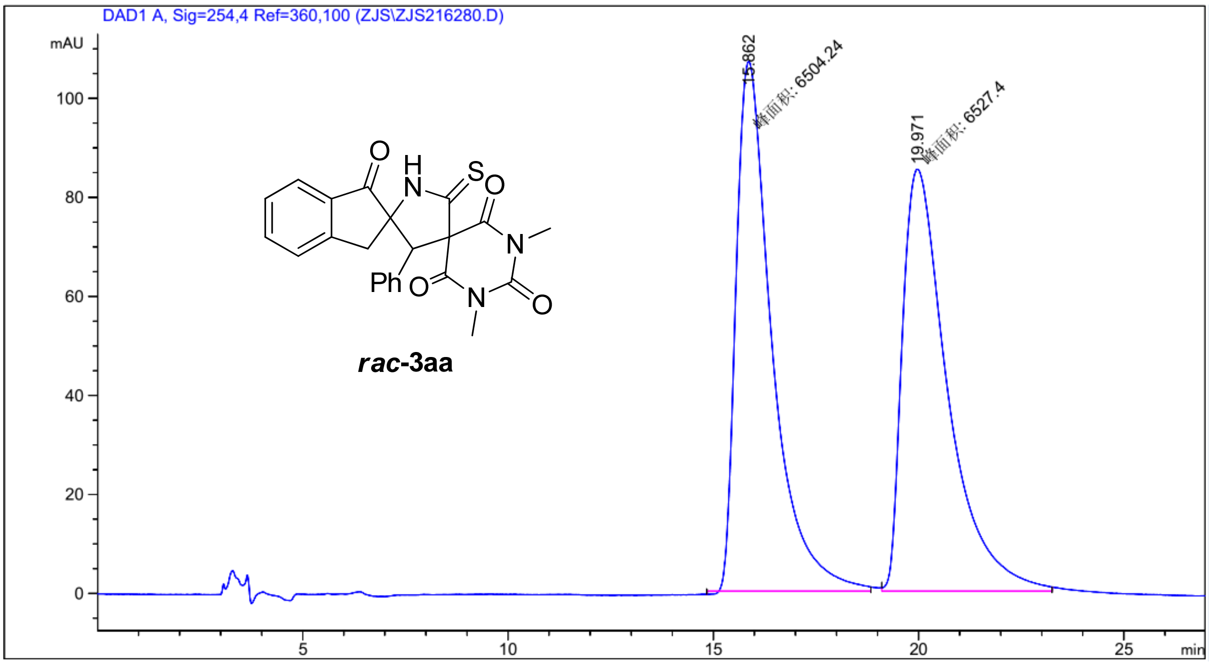

Peak #	RetTime [min]	Type	Width [min]	Area [mAU*s]	Height [mAU]	Area %
1	15.862	MM	1.0129	6504.24268	107.02784	49.9112
2	19.971	MM	1.2765	6527.39893	85.22320	50.0888

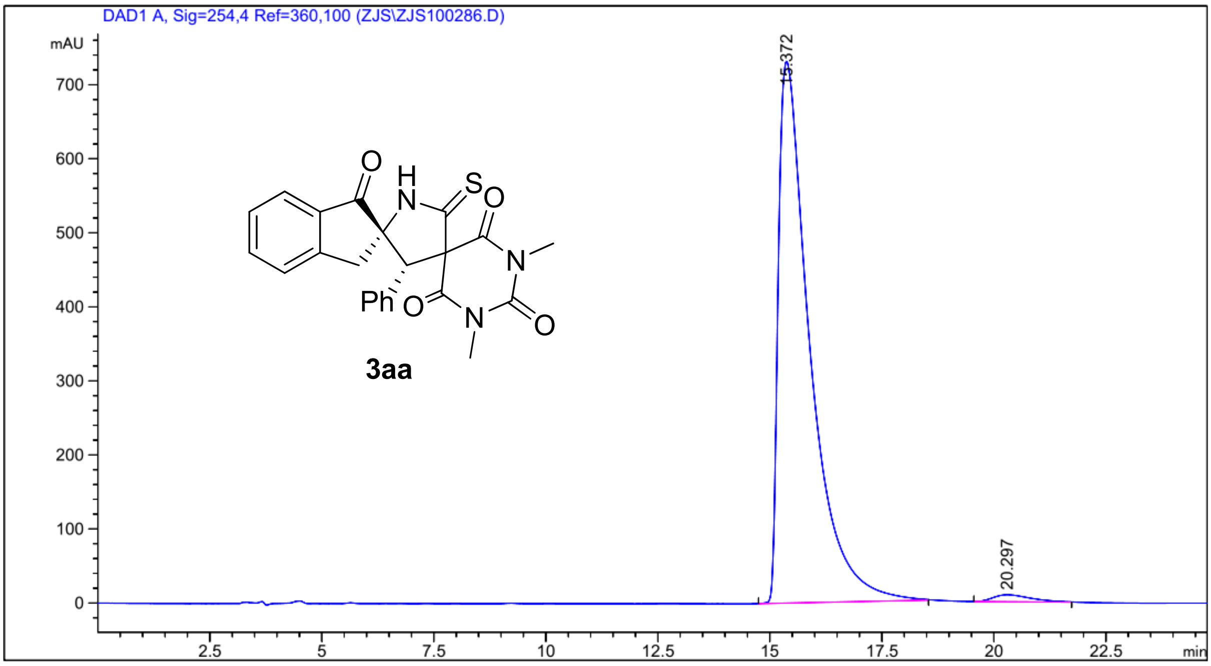

Peak #	RetTime [min]	Type	Width [min]	Area [mAU*s]	Height [mAU]	Area %
1	15.372	BB	0.7252	3.58248e4	731.72662	98.4930
2	20.297	BB	0.7908	548.13196	9.36580	1.5070

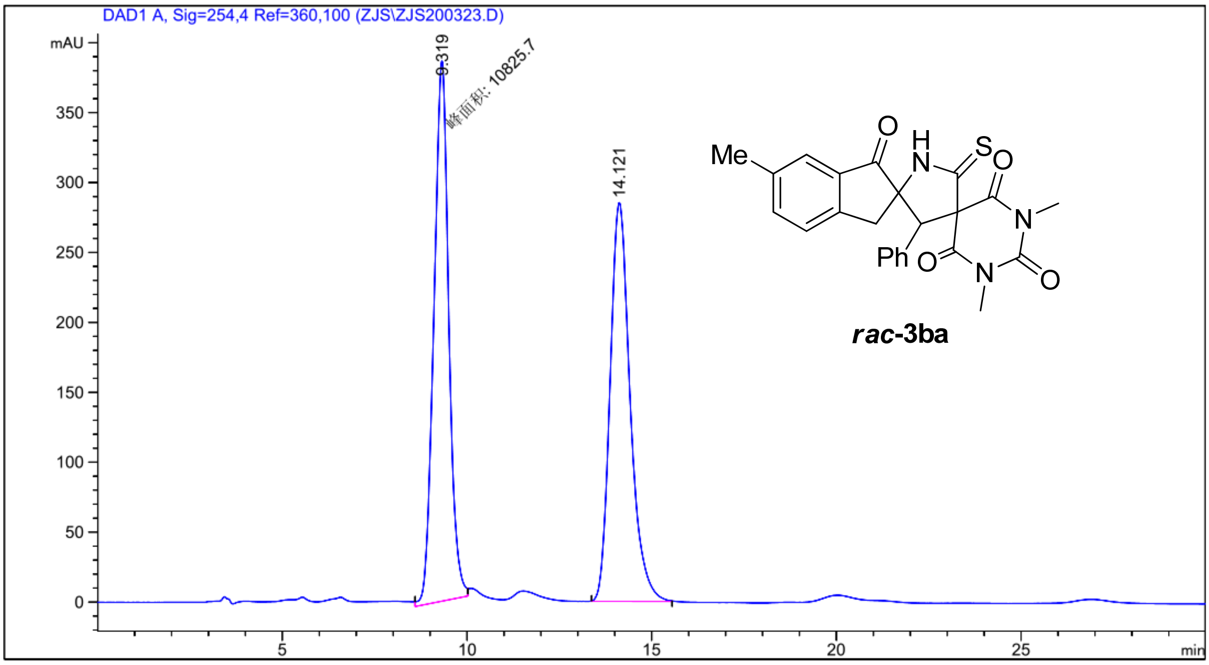

Peak #	RetTime [min]	Type	Width [min]	Area [mAU*s]	Height [mAU]	Area %
1	9.319	MM	0.4670	1.08257e4	386.35458	50.1548
2	14.121	BB	0.5742	1.07588e4	285.15967	49.8452

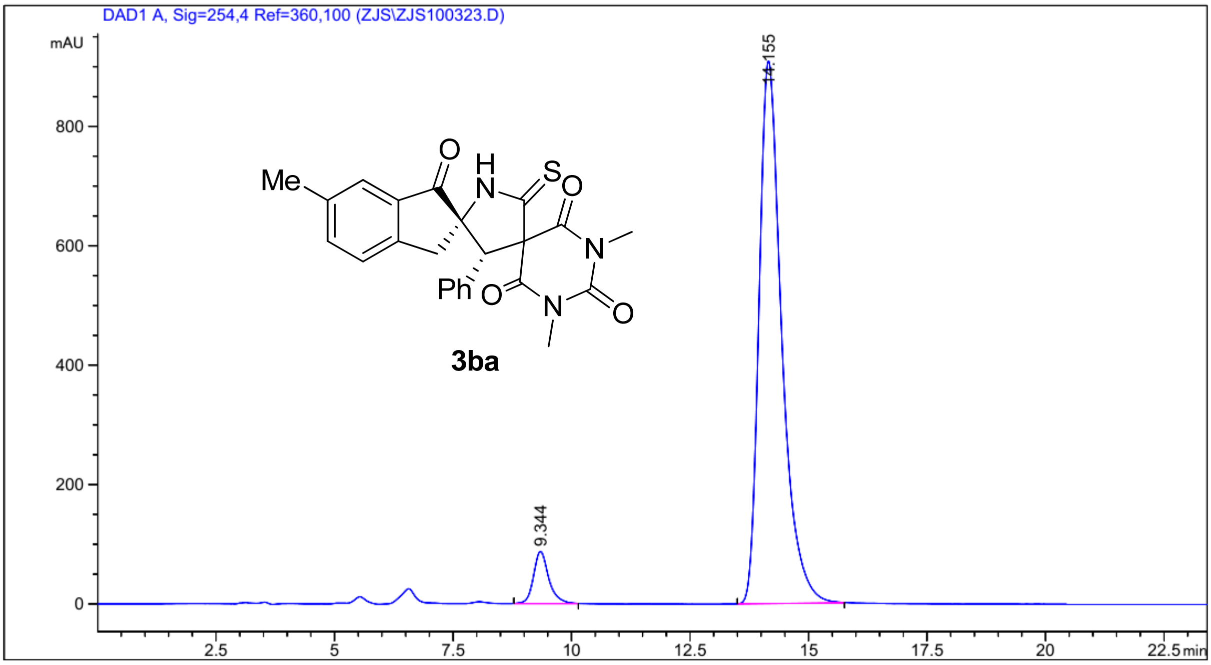

Peak #	RetTime [min]	Type	Width [min]	Area [mAU*s]	Height [mAU]	Area %
1	9.344	BB	0.3380	1963.57336	87.43686	6.0949
2	14.155	BB	0.5022	3.02532e4	909.13275	93.9051

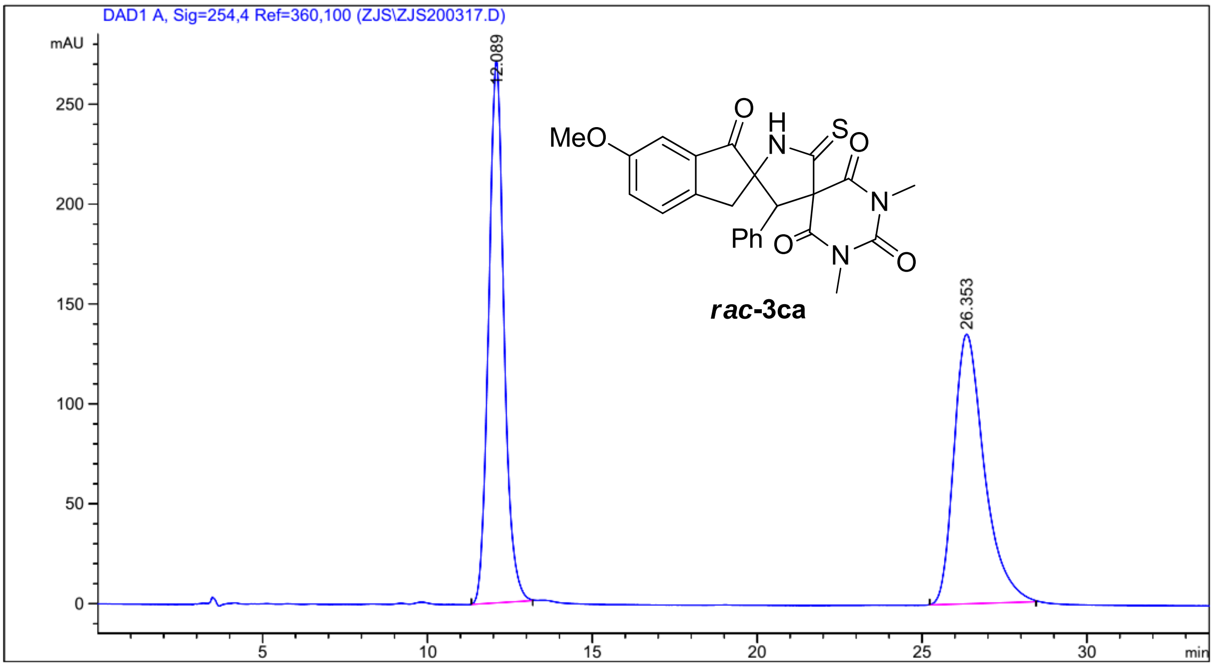

***rac*-3ca**

Peak #	RetTime [min]	Type	Width [min]	Area [mAU*s]	Height [mAU]	Area %
1	12.089	BB	0.4966	8799.12793	271.16763	50.2979
2	26.353	BB	0.9719	8694.90625	134.91916	49.7021

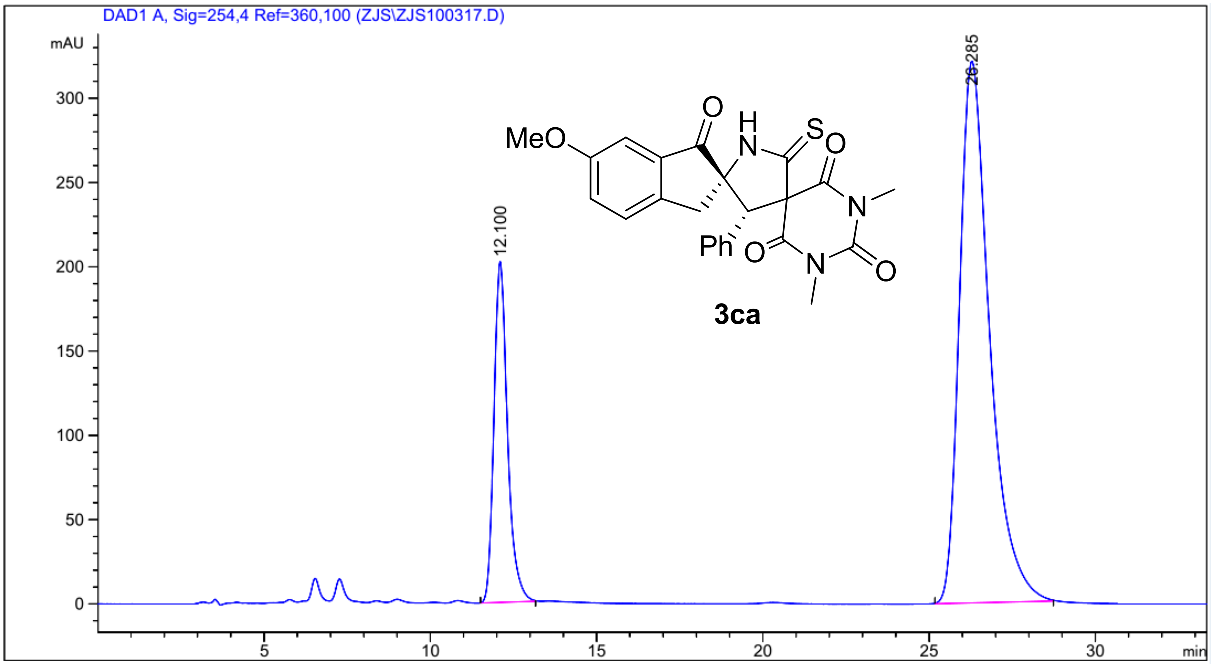

3ca

Peak #	RetTime [min]	Type	Width [min]	Area [mAU*s]	Height [mAU]	Area %
1	12.100	BB	0.4334	5742.90625	202.14685	22.0987
2	26.285	BB	0.9411	2.02447e4	321.30673	77.9013

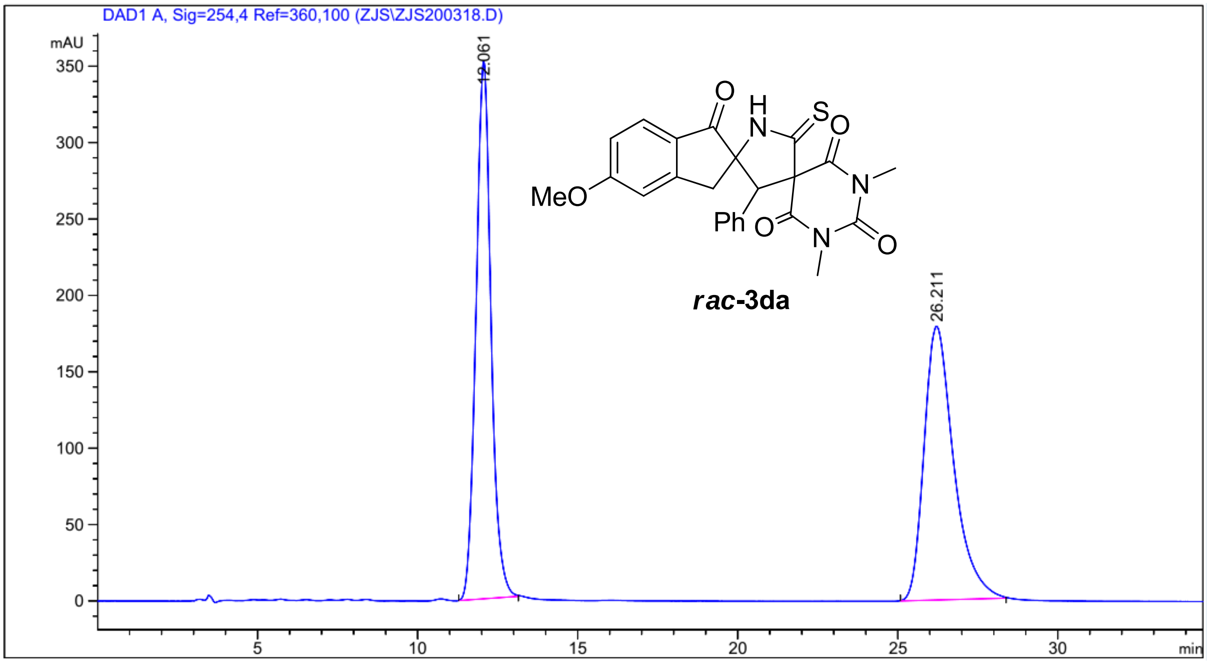

Peak #	RetTime [min]	Type	Width [min]	Area [mAU*s]	Height [mAU]	Area %
1	12.061	BB	0.4974	1.15116e4	352.09341	49.9219
2	26.211	BB	0.9780	1.15476e4	179.14830	50.0781

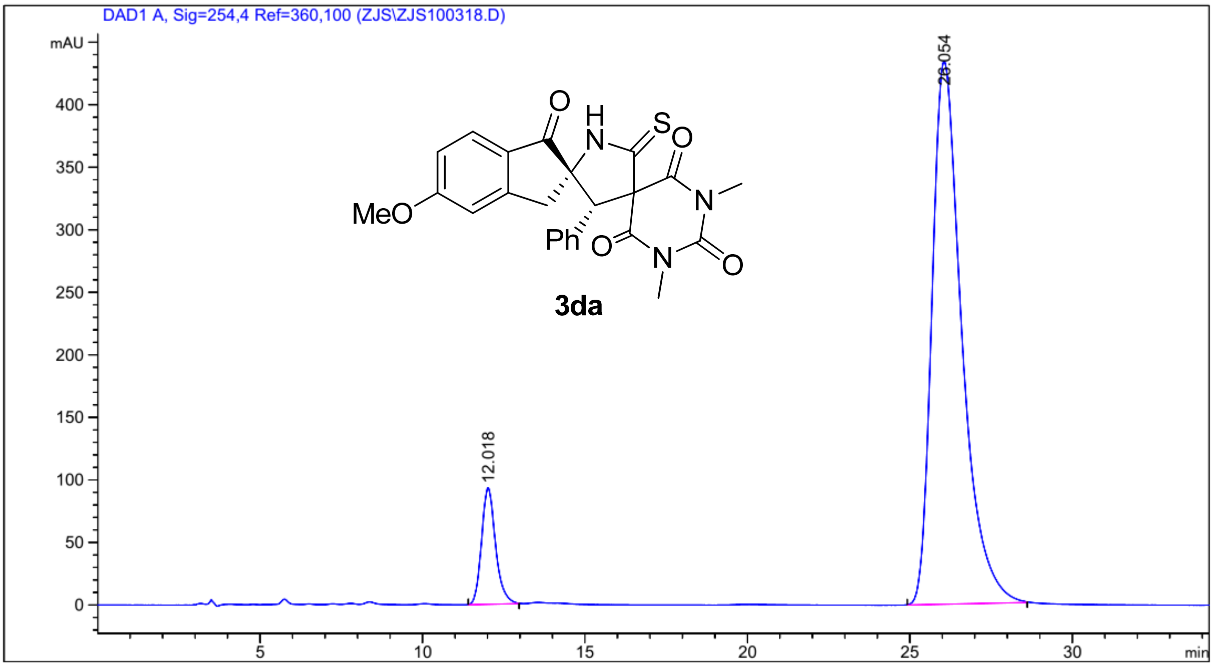

Peak #	RetTime [min]	Type	Width [min]	Area [mAU*s]	Height [mAU]	Area %
1	12.018	BB	0.4597	2807.75073	93.14551	9.1983
2	26.054	BB	0.9587	2.77168e4	434.22656	90.8017

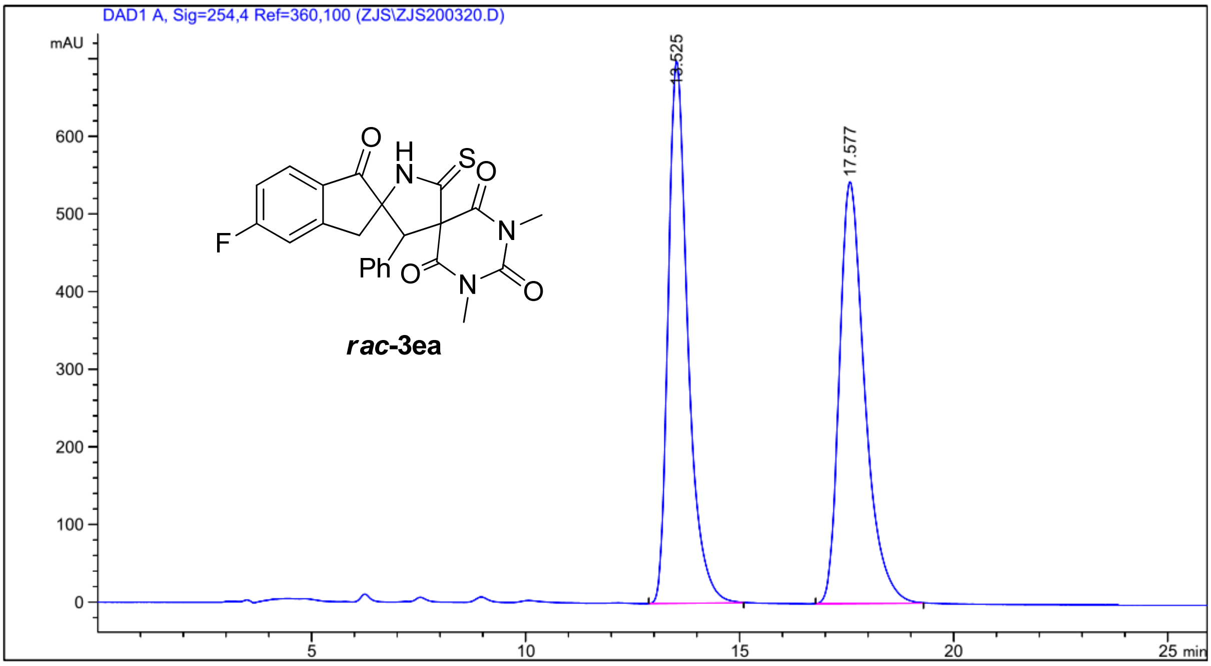

Peak #	RetTime [min]	Type	Width [min]	Area [mAU*s]	Height [mAU]	Area %
1	13.525	BB	0.4866	2.24270e4	698.38226	49.9364
2	17.577	BB	0.6308	2.24842e4	543.27991	50.0636

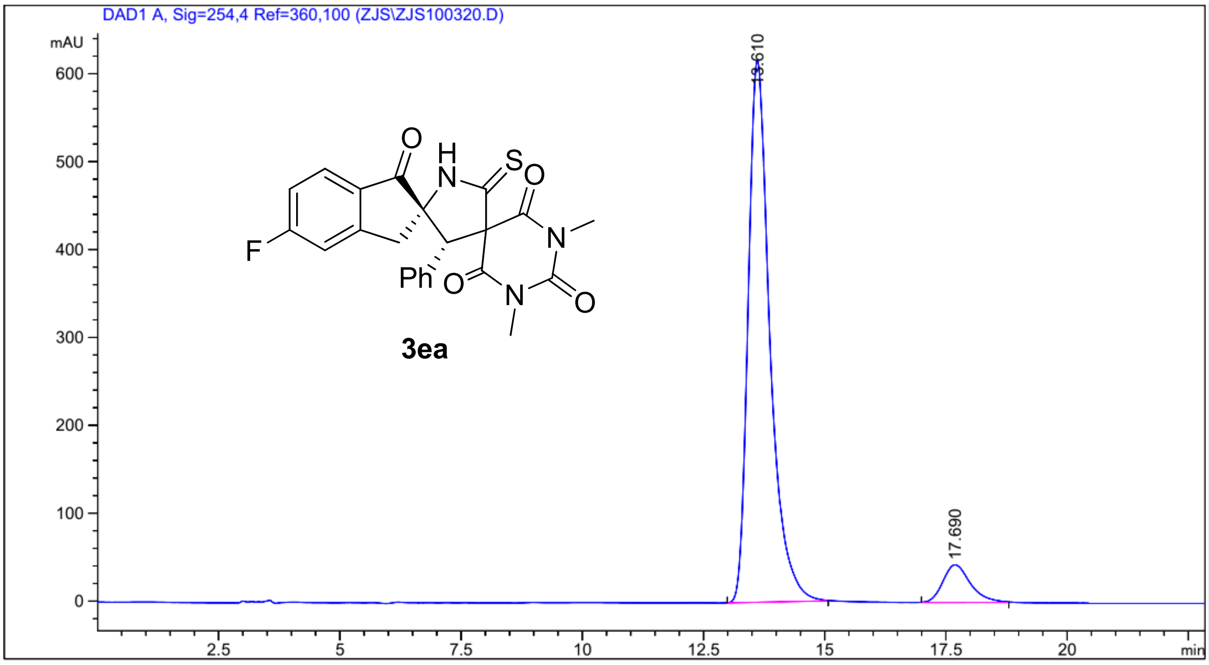

Peak #	RetTime [min]	Type	Width [min]	Area [mAU*s]	Height [mAU]	Area %
1	13.610	BB	0.4712	1.91773e4	616.06366	91.9180
2	17.690	BB	0.5939	1686.18091	42.77147	8.0820

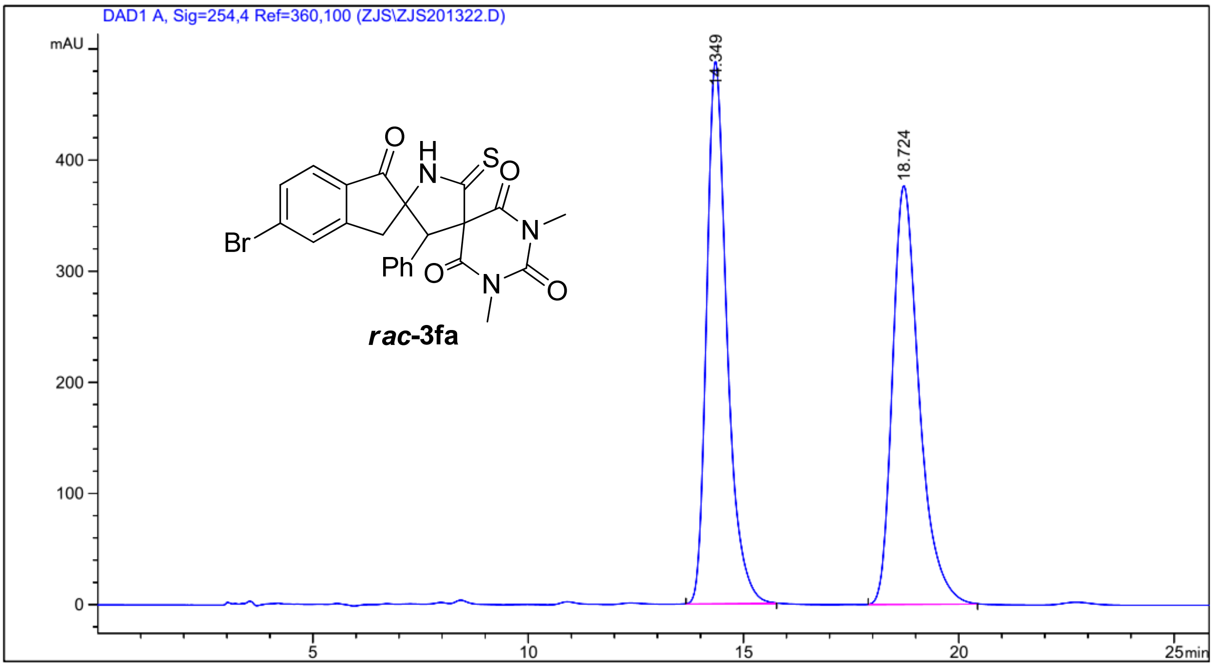

Peak #	RetTime [min]	Type	Width [min]	Area [mAU*s]	Height [mAU]	Area %
1	14.349	BB	0.5007	1.60945e4	488.06161	49.7357
2	18.724	BB	0.6518	1.62656e4	376.69672	50.2643

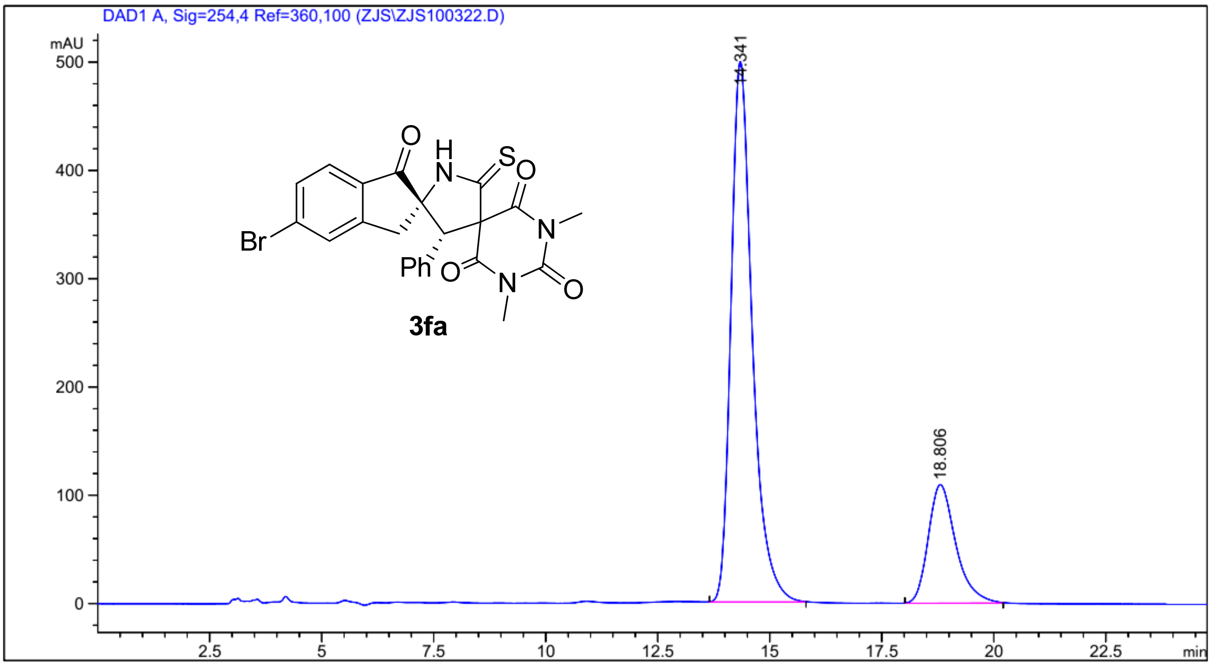

Peak #	RetTime [min]	Type	Width [min]	Area [mAU*s]	Height [mAU]	Area %
1	14.341	BB	0.5002	1.65249e4	499.19998	77.9135
2	18.806	BB	0.6534	4684.39844	109.44051	22.0865

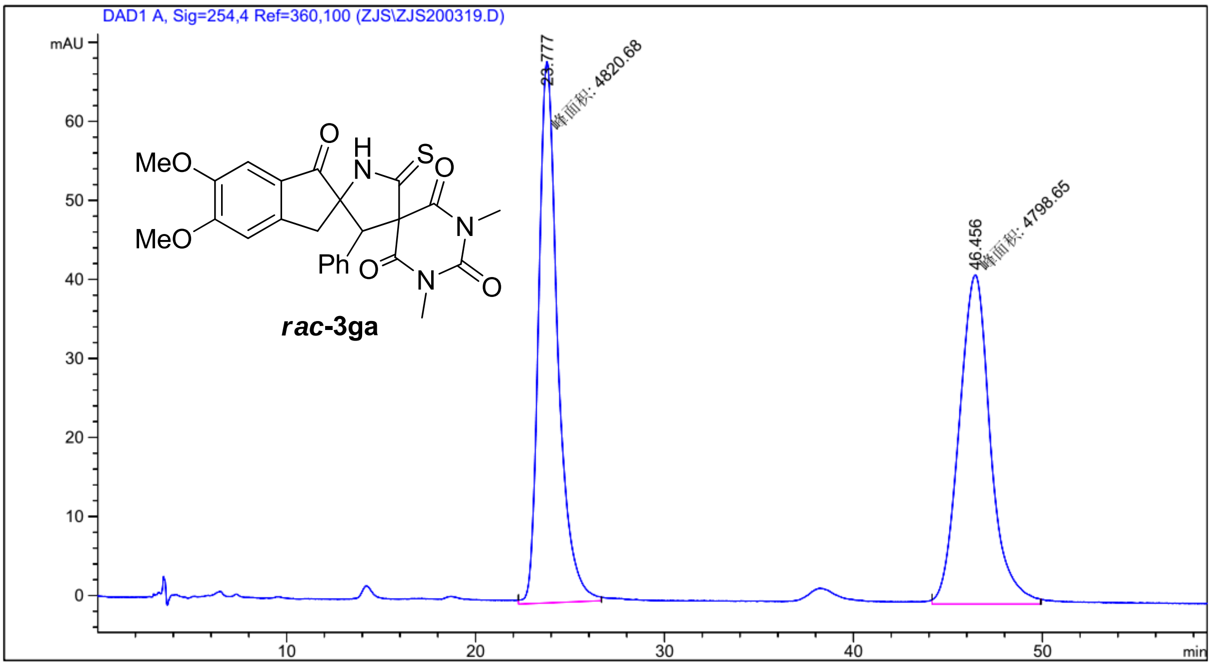

Peak #	RetTime [min]	Type	Width [min]	Area [mAU*s]	Height [mAU]	Area %
1	23.777	MM	1.1725	4820.68408	68.52416	50.1146
2	46.456	MM	1.9190	4798.64502	41.67612	49.8854

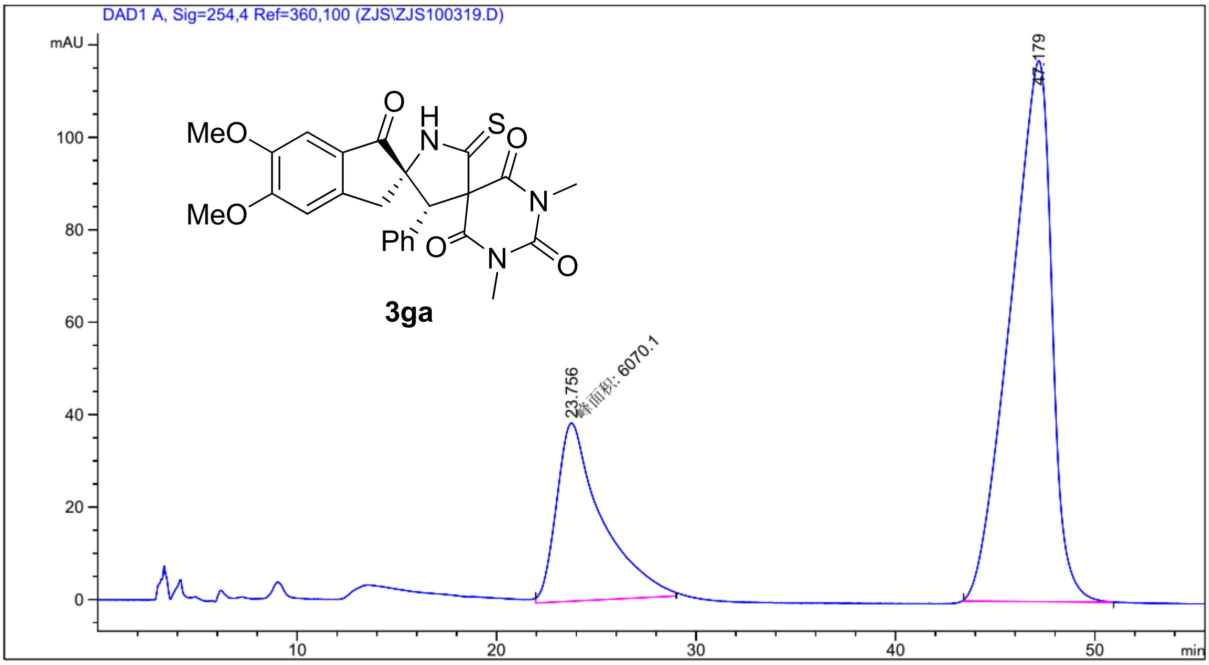

Peak #	RetTime [min]	Type	Width [min]	Area [mAU*s]	Height [mAU]	Area %
1	23.756	MM	2.6244	6070.09961	38.54853	26.4741
2	47.179	BB	1.7366	1.68583e4	117.03114	73.5259

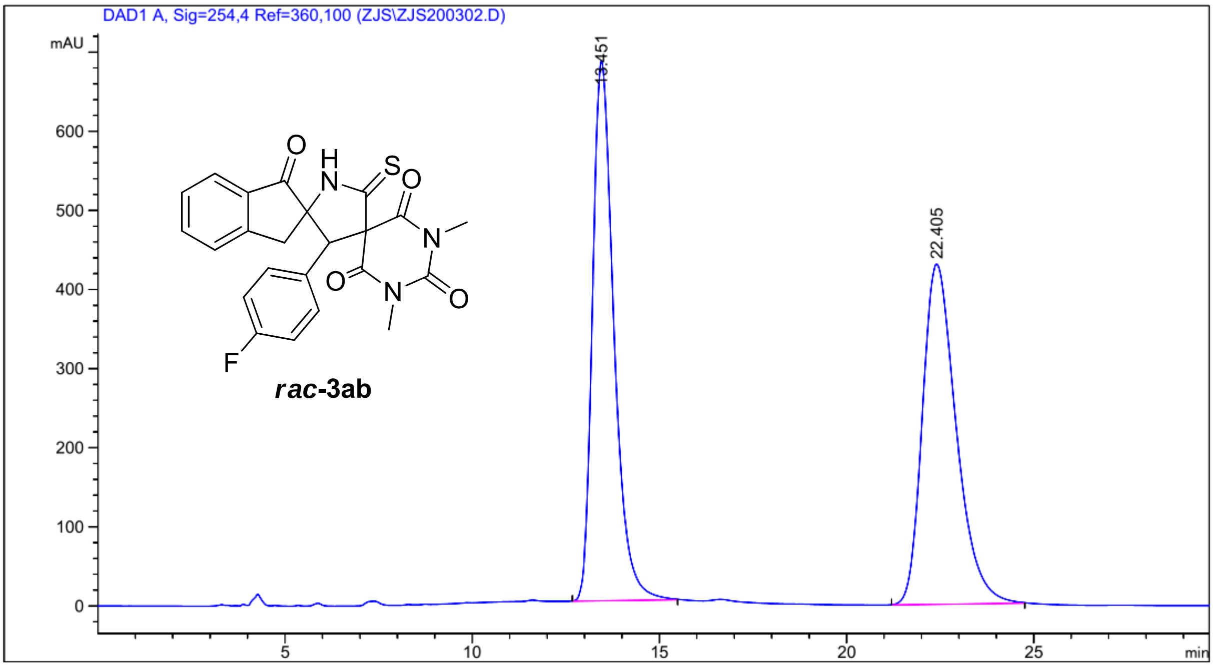

Peak #	RetTime [min]	Type	Width [min]	Area [mAU*s]	Height [mAU]	Area %
1	13.451	BB	0.6055	2.70965e4	682.02148	50.0609
2	22.405	BB	0.9609	2.70306e4	430.25720	49.9391

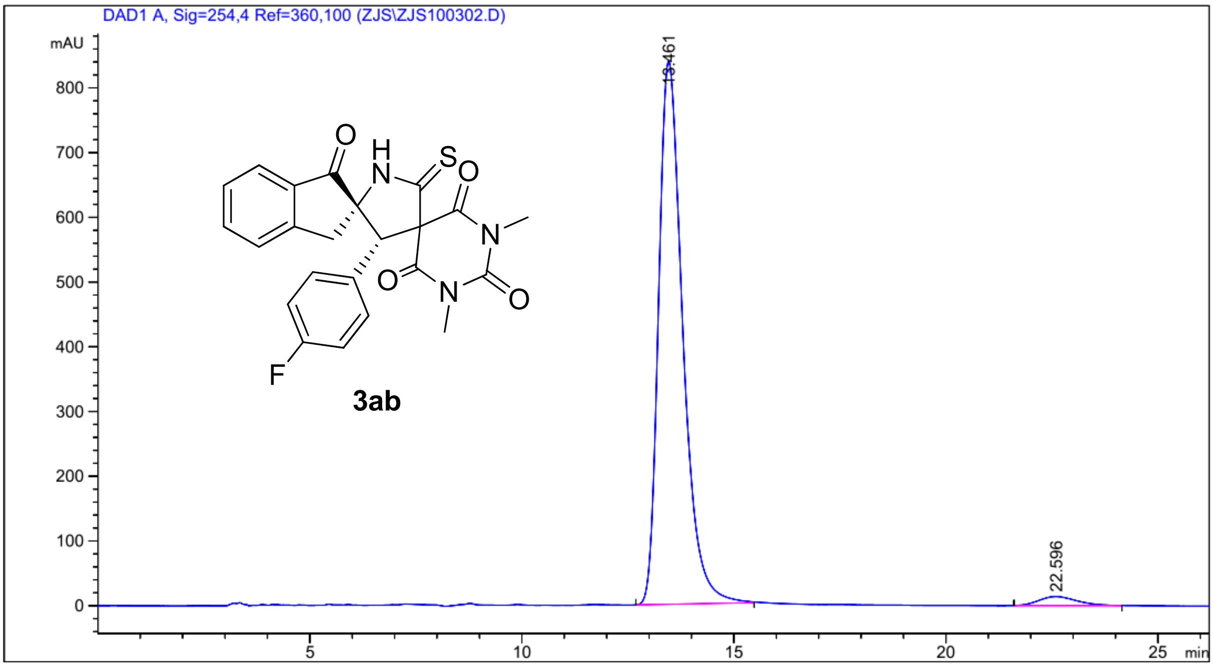

Peak #	RetTime [min]	Type	Width [min]	Area [mAU*s]	Height [mAU]	Area %
1	13.461	BB	0.6039	3.32203e4	839.00397	97.4370
2	22.596	BB	0.7459	873.82336	13.96633	2.5630

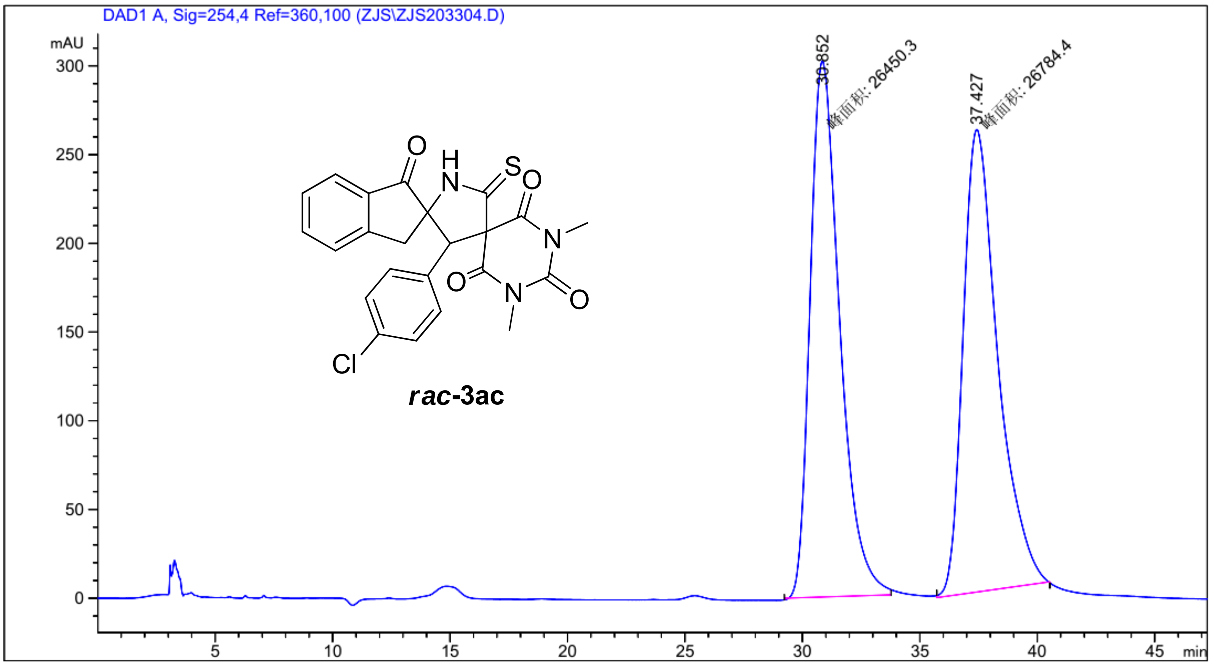

Peak #	RetTime [min]	Type	Width [min]	Area [mAU*s]	Height [mAU]	Area %
1	30.852	MM	1.4595	2.64503e4	302.04819	49.6862
2	37.427	MM	1.7118	2.67844e4	260.78601	50.3138

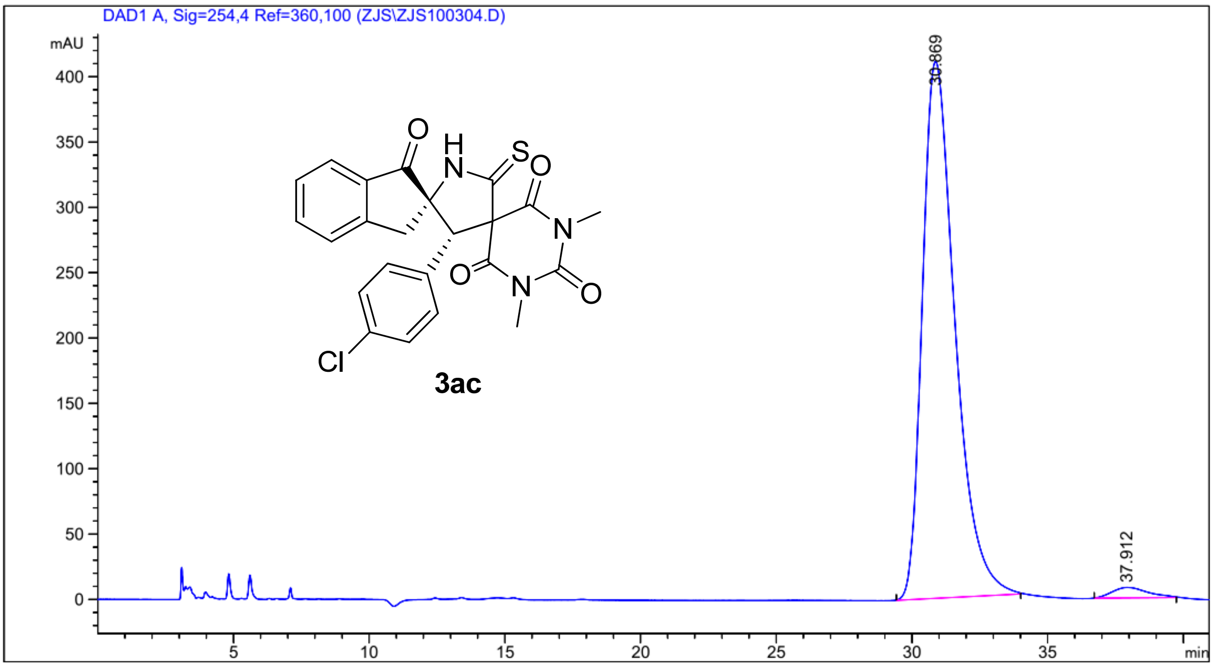

Peak #	RetTime [min]	Type	Width [min]	Area [mAU*s]	Height [mAU]	Area %
1	30.869	BB	1.2486	3.43173e4	410.81244	97.9087
2	37.912	BB	1.0870	733.00610	7.98487	2.0913

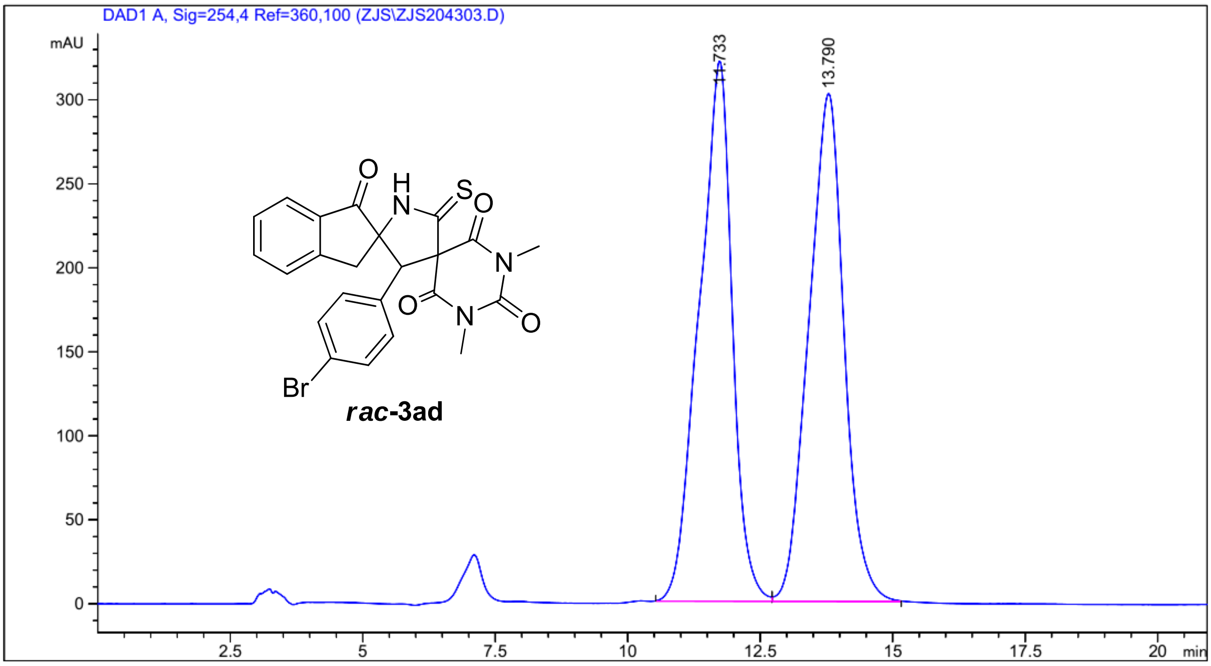

Peak #	RetTime [min]	Type	Width [min]	Area [mAU*s]	Height [mAU]	Area %
1	11.733	BV	0.6126	1.39661e4	321.63046	49.9270
2	13.790	VB	0.6744	1.40070e4	302.39828	50.0730

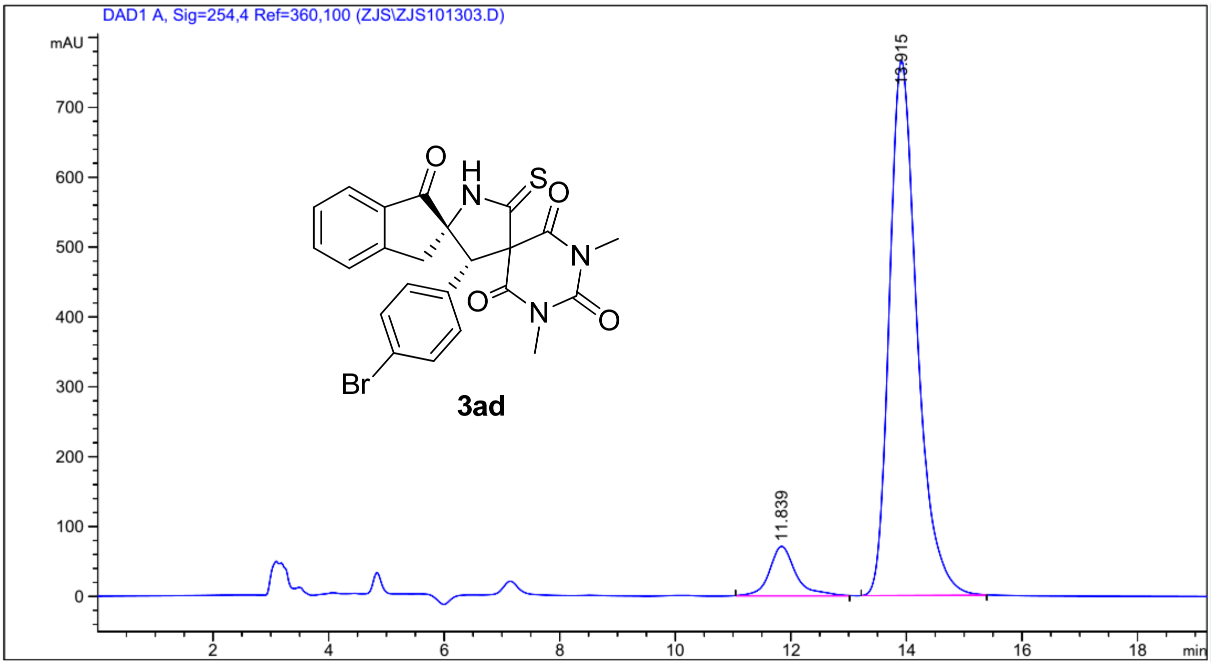

Peak #	RetTime [min]	Type	Width [min]	Area [mAU*s]	Height [mAU]	Area %
1	11.839	BB	0.4723	2247.10937	70.79588	8.0859
2	13.915	BB	0.5095	2.55433e4	765.16919	91.9141

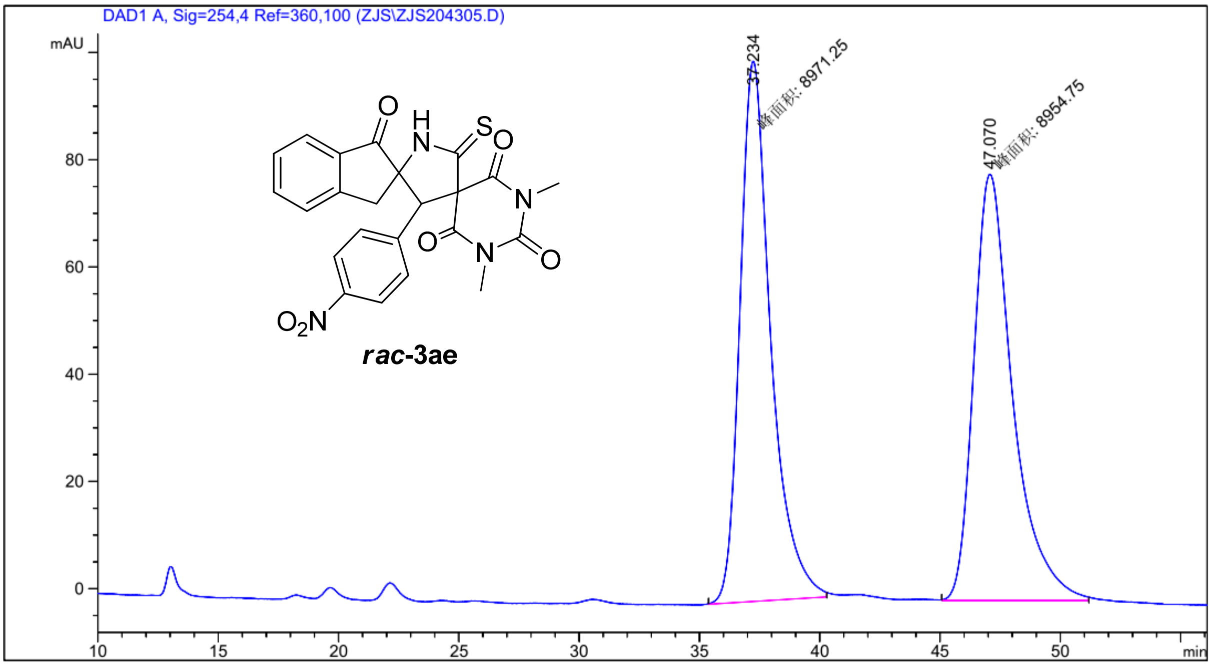

Peak #	RetTime [min]	Type	Width [min]	Area [mAU*s]	Height [mAU]	Area %
1	37.234	MM	1.4839	8971.24707	100.76543	50.0460
2	47.070	MM	1.8765	8954.75000	79.53210	49.9540

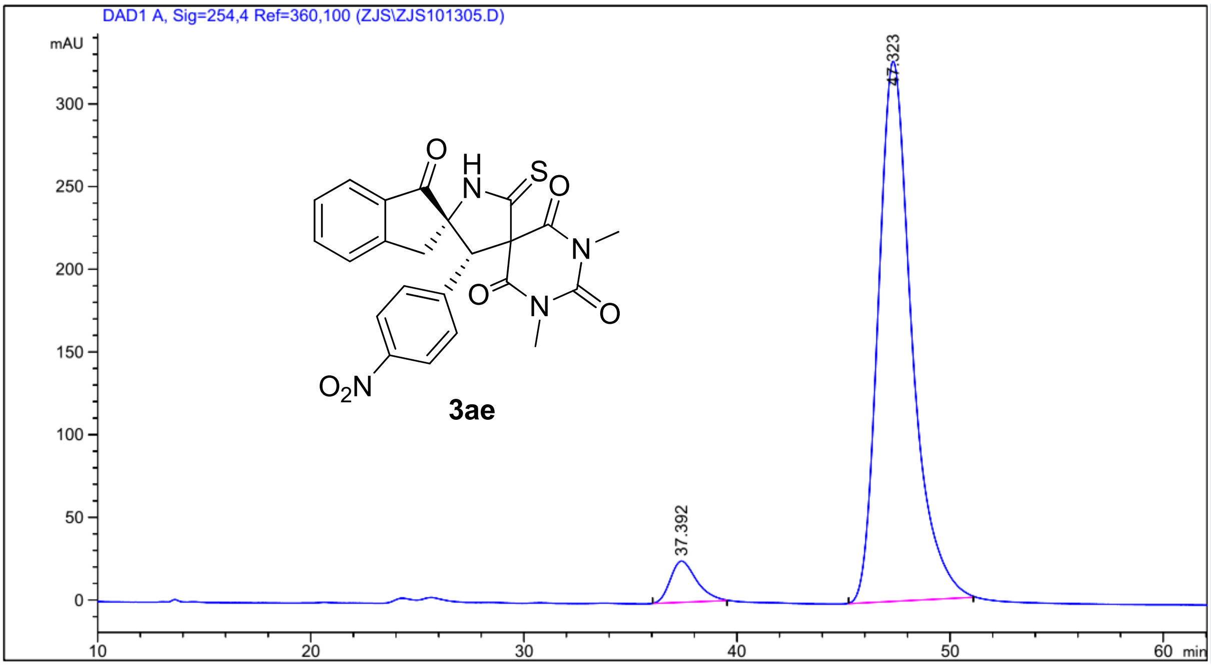

Peak #	RetTime [min]	Type	Width [min]	Area [mAU*s]	Height [mAU]	Area %
1	37.392	BB	1.0173	2143.08276	25.02088	5.6457
2	47.323	BB	1.5836	3.58164e4	326.55856	94.3543

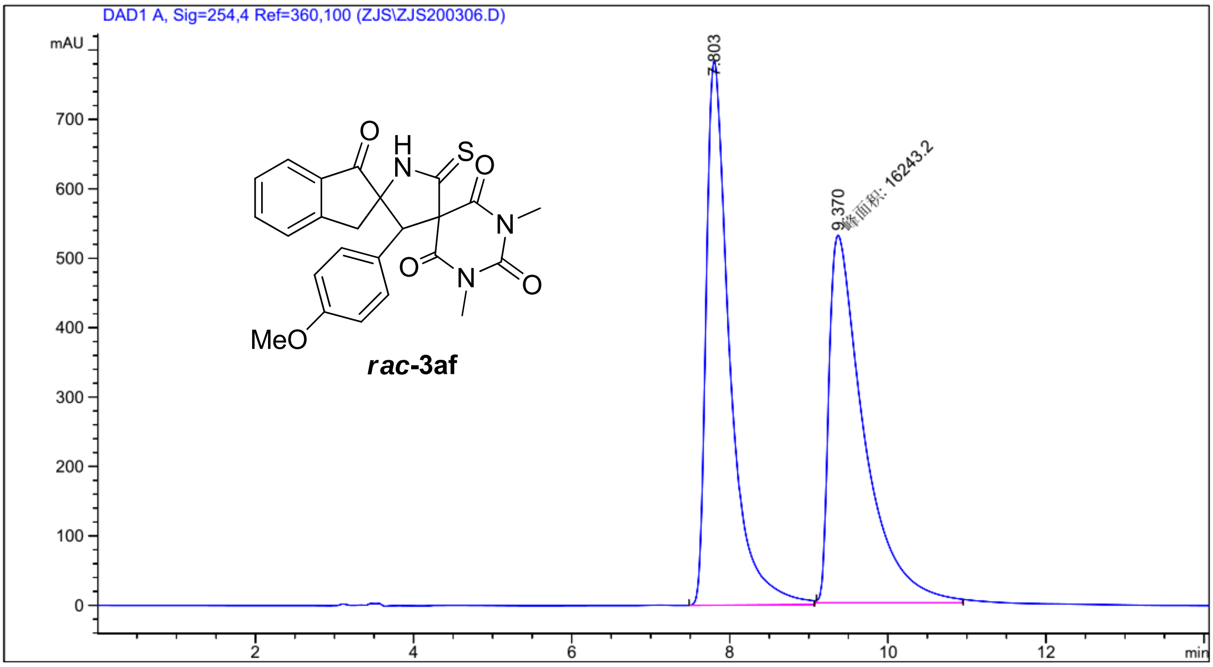

Peak #	RetTime [min]	Type	Width [min]	Area [mAU*s]	Height [mAU]	Area %
1	7.803	BV	0.3085	1.62956e4	784.05957	50.0804
2	9.370	MM	0.5114	1.62432e4	529.41125	49.9196

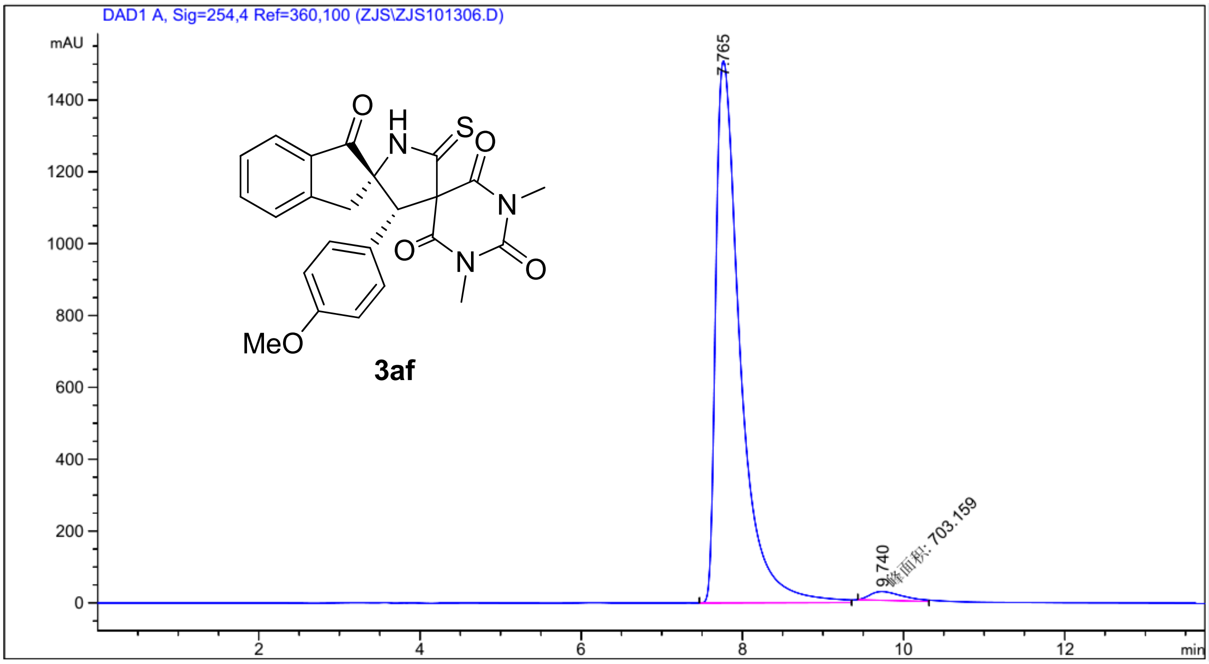

Peak #	RetTime [min]	Type	Width [min]	Area [mAU*s]	Height [mAU]	Area %
1	7.765	BV	0.3135	3.17567e4	1509.03186	97.8338
2	9.740	MM	0.4793	703.15900	24.45270	2.1662

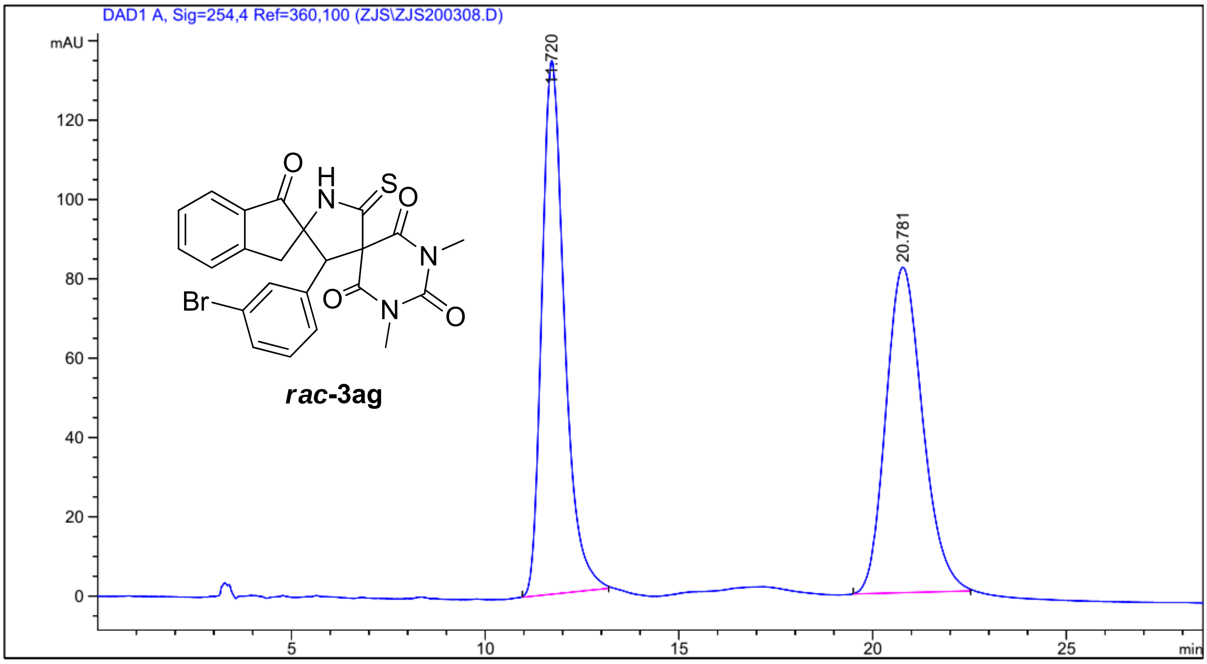

Peak #	RetTime [min]	Type	Width [min]	Area [mAU*s]	Height [mAU]	Area %
1	11.720	BB	0.6210	5475.66895	134.50122	50.2172
2	20.781	BB	1.0038	5428.31006	82.05406	49.7828

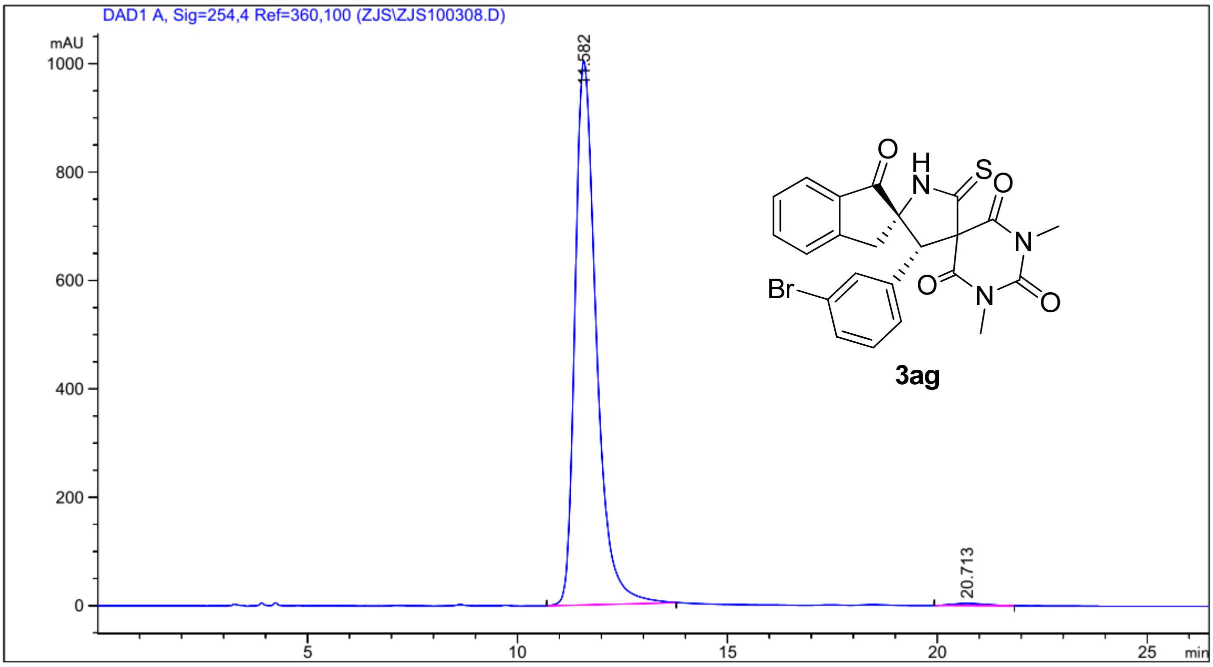

Peak #	RetTime [min]	Type	Width [min]	Area [mAU*s]	Height [mAU]	Area %
1	11.582	BB	0.5387	3.54959e4	1003.45917	99.3638
2	20.713	BB	0.6622	227.28185	4.02885	0.6362

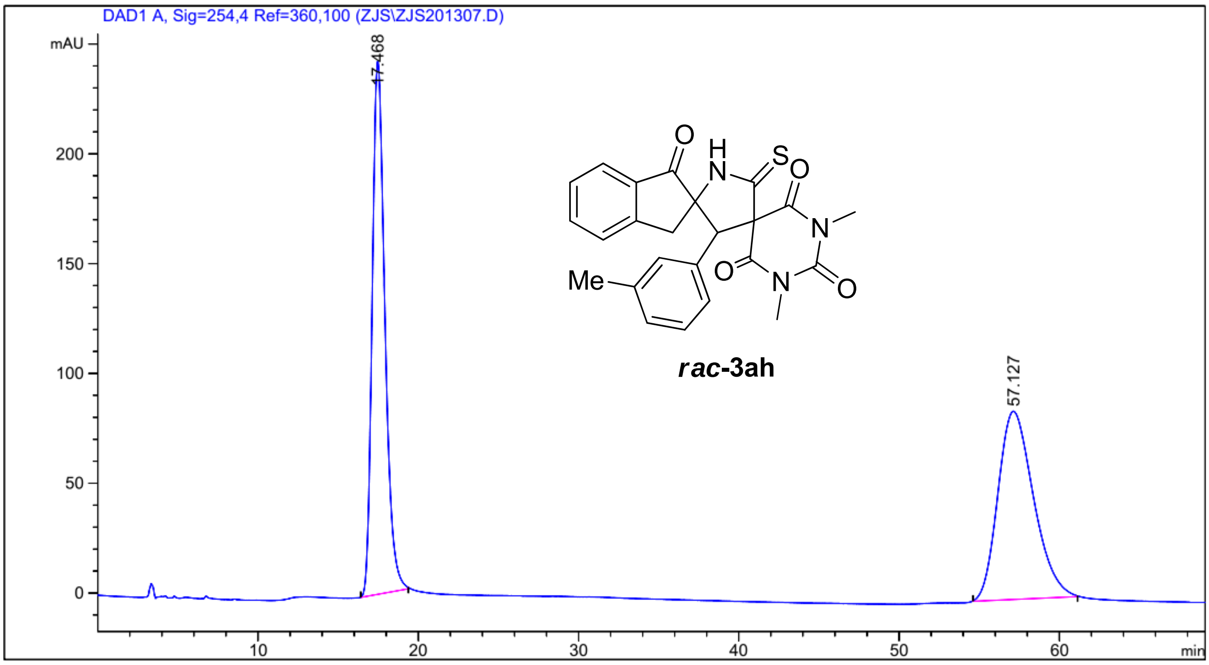

Peak #	RetTime [min]	Type	Width [min]	Area [mAU*s]	Height [mAU]	Area %
1	17.468	BB	0.8448	1.33741e4	242.72838	50.2835
2	57.127	BB	1.8523	1.32233e4	85.69221	49.7165

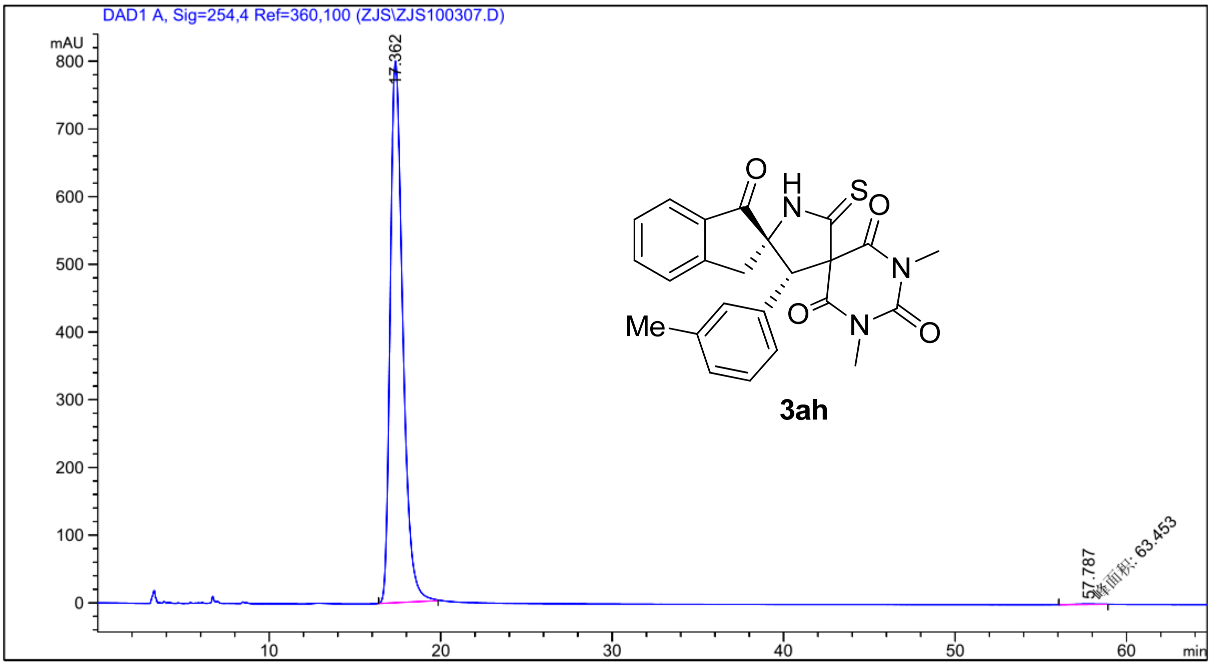

Peak #	RetTime [min]	Type	Width [min]	Area [mAU*s]	Height [mAU]	Area %
1	17.362	BB	0.7837	4.08604e4	800.21143	99.8449
2	57.787	MM	1.6904	63.45302	6.25603e-1	0.1551

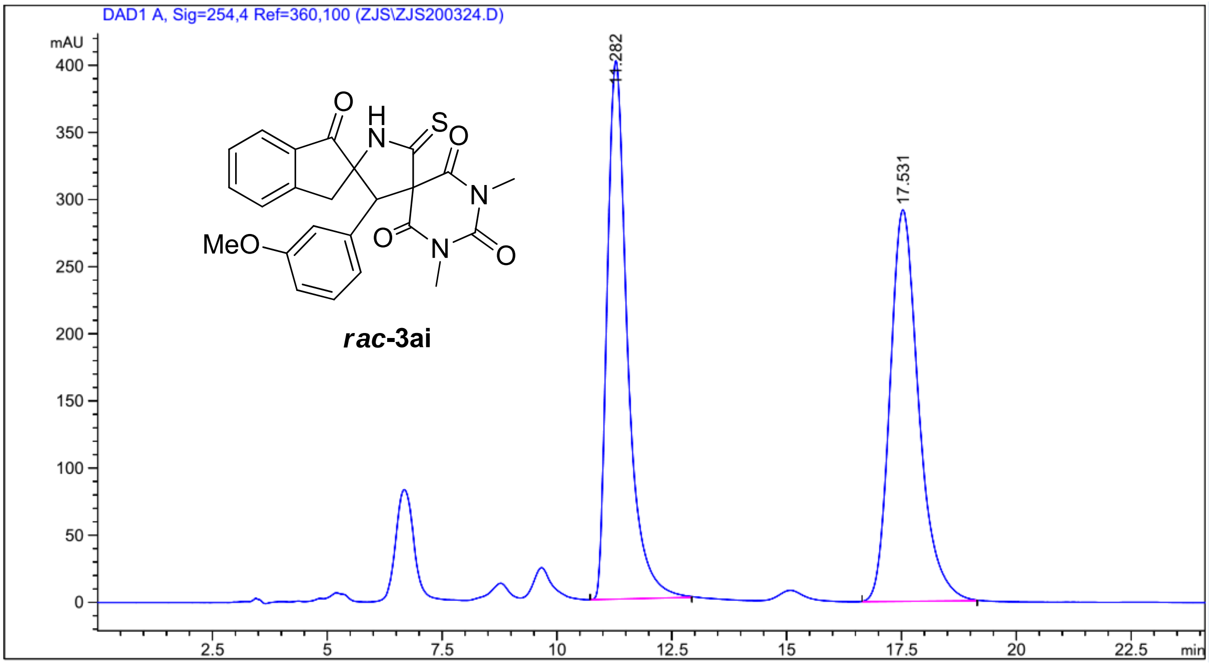

Peak #	RetTime [min]	Type	Width [min]	Area [mAU*s]	Height [mAU]	Area %
1	11.282	BB	0.4736	1.23585e4	400.96426	49.3913
2	17.531	BB	0.6587	1.26631e4	291.54507	50.6087

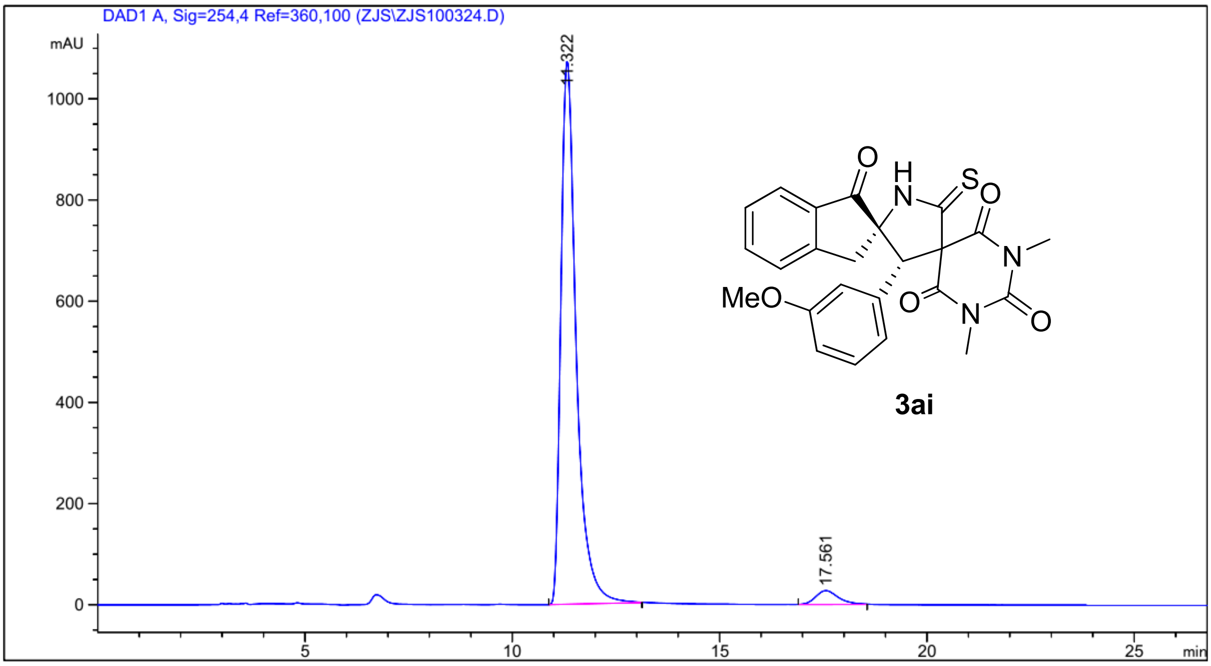

Peak #	RetTime [min]	Type	Width [min]	Area [mAU*s]	Height [mAU]	Area %
1	11.322	BB	0.3931	2.76977e4	1073.25452	96.3710
2	17.561	BB	0.5718	1043.01343	27.29837	3.6290

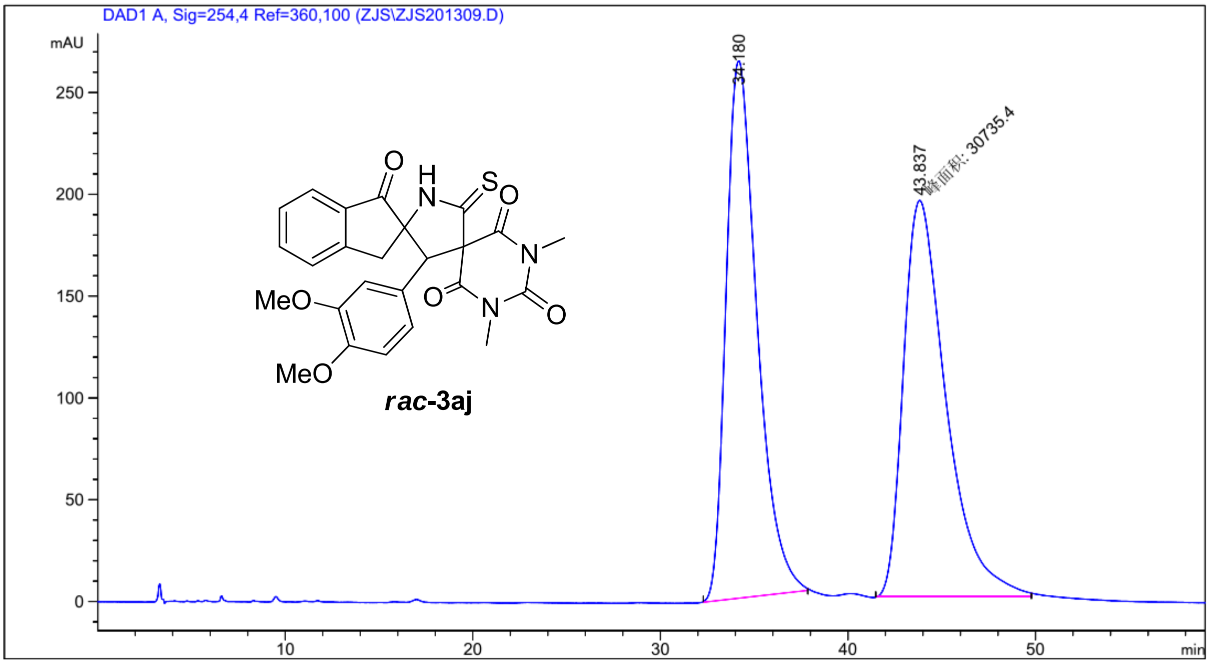

Peak #	RetTime [min]	Type	Width [min]	Area [mAU*s]	Height [mAU]	Area %
1	34.180	BB	1.6614	3.03928e4	263.94412	49.7197
2	43.837	MM	2.6339	3.07354e4	194.48611	50.2803

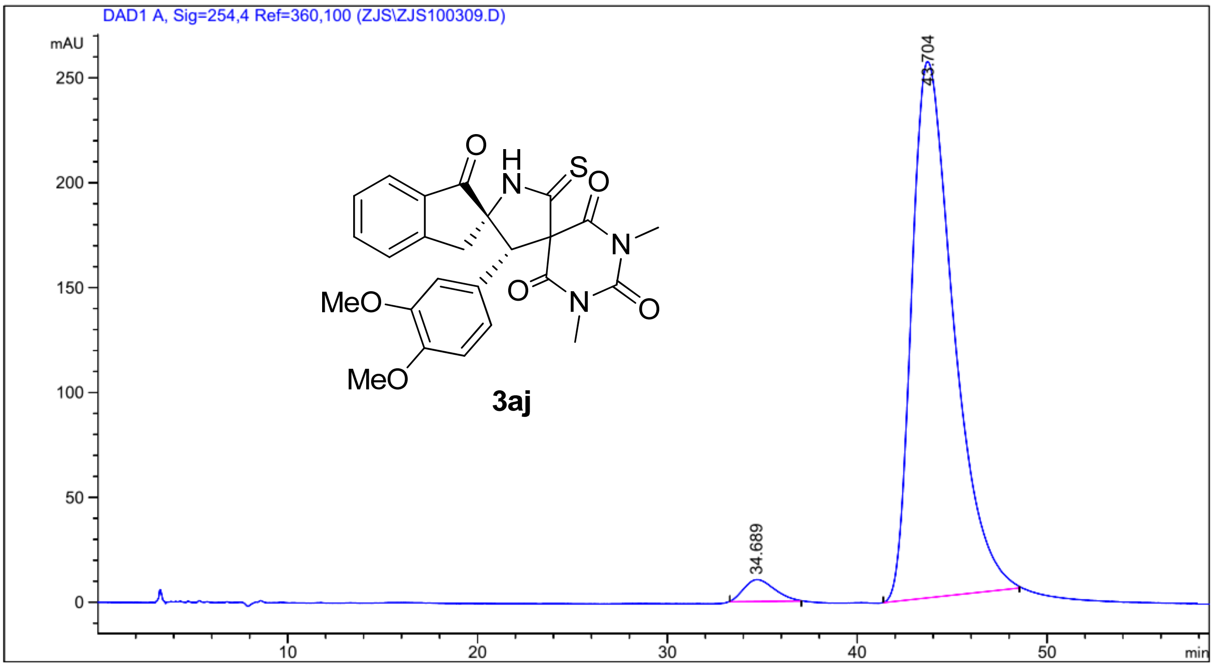

Peak #	RetTime [min]	Type	Width [min]	Area [mAU*s]	Height [mAU]	Area %
1	34.689	BB	1.3222	1166.54004	10.40314	2.8996
2	43.704	BB	2.0985	3.90648e4	255.68781	97.1004

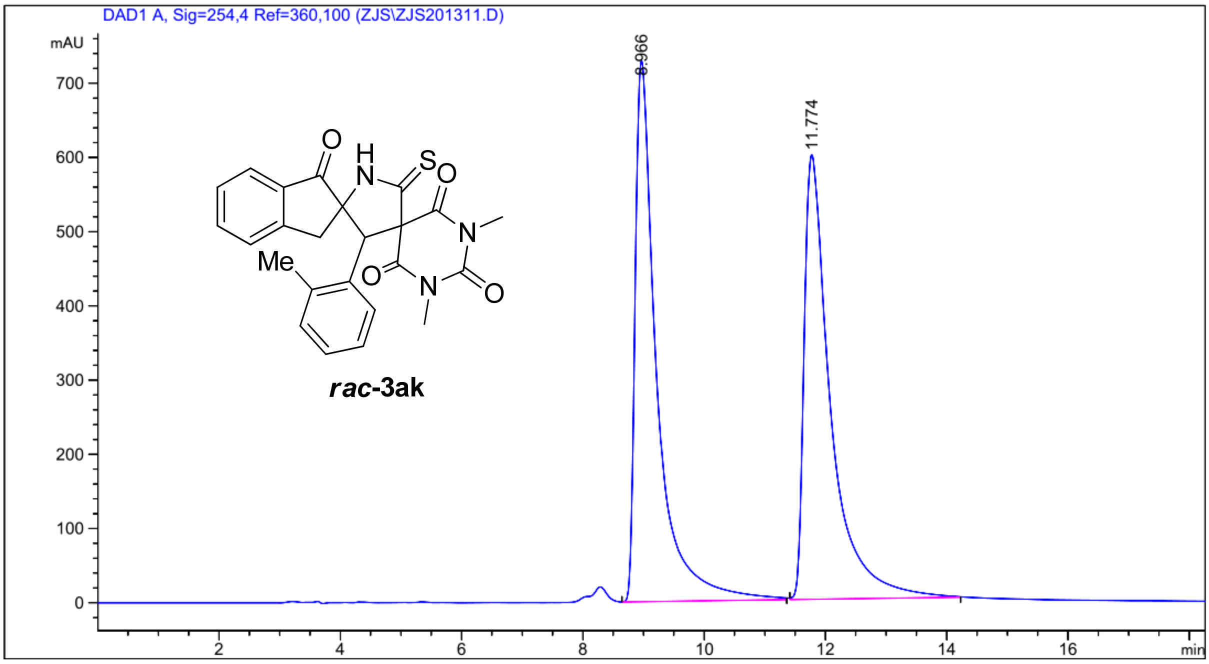

Peak #	RetTime [min]	Type	Width [min]	Area [mAU*s]	Height [mAU]	Area %
1	8.966	VB	0.3602	1.81290e4	728.87714	49.9460
2	11.774	BB	0.4400	1.81682e4	598.79614	50.0540

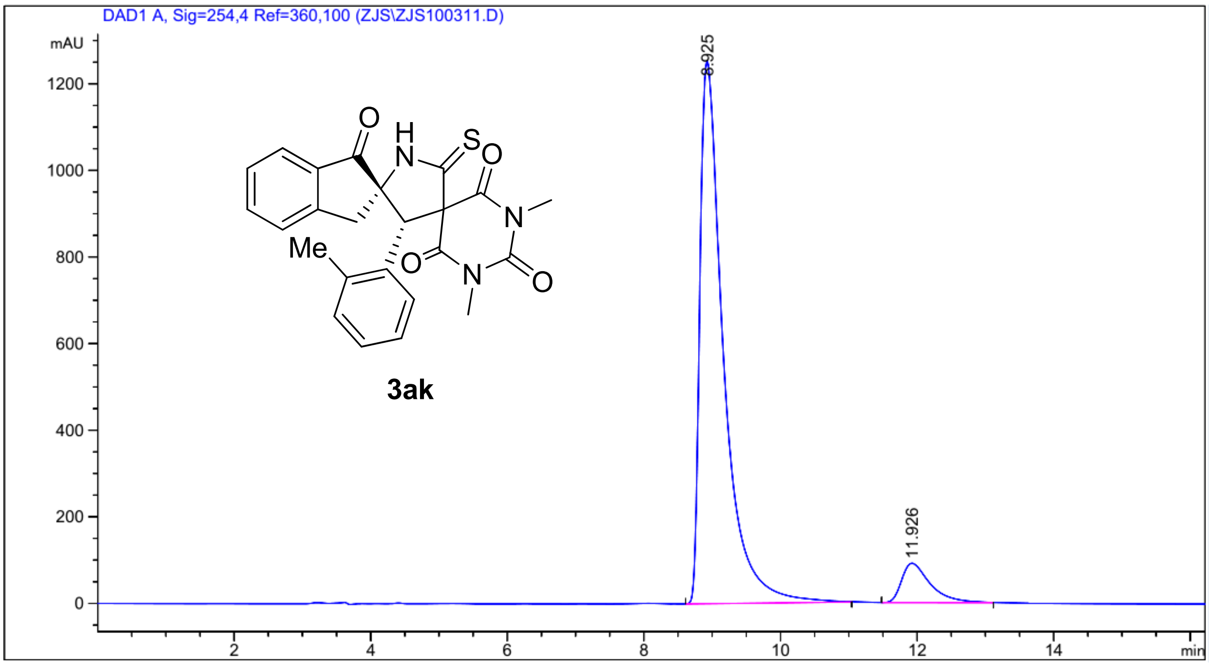

Peak #	RetTime [min]	Type	Width [min]	Area [mAU*s]	Height [mAU]	Area %
1	8.925	BB	0.3563	3.00928e4	1252.62610	91.9349
2	11.926	BB	0.4379	2639.91650	90.62246	8.0651

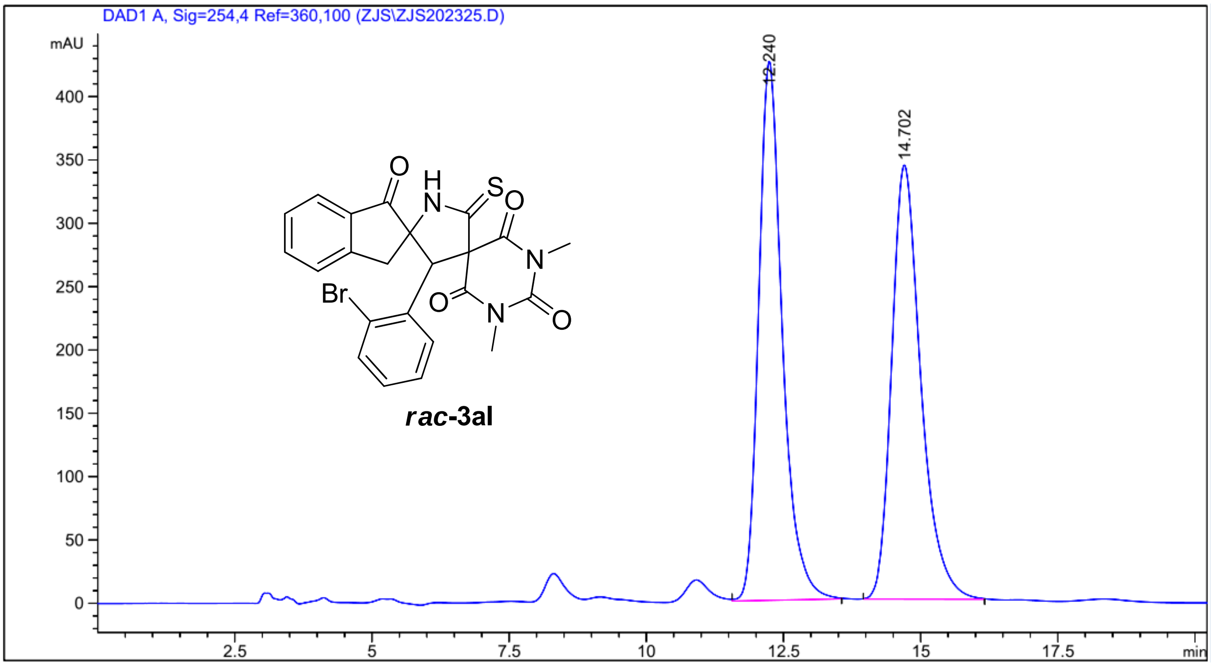

Peak #	RetTime [min]	Type	Width [min]	Area [mAU*s]	Height [mAU]	Area %
1	12.240	VB	0.4574	1.28199e4	425.57092	50.2511
2	14.702	BB	0.5602	1.26918e4	342.58719	49.7489

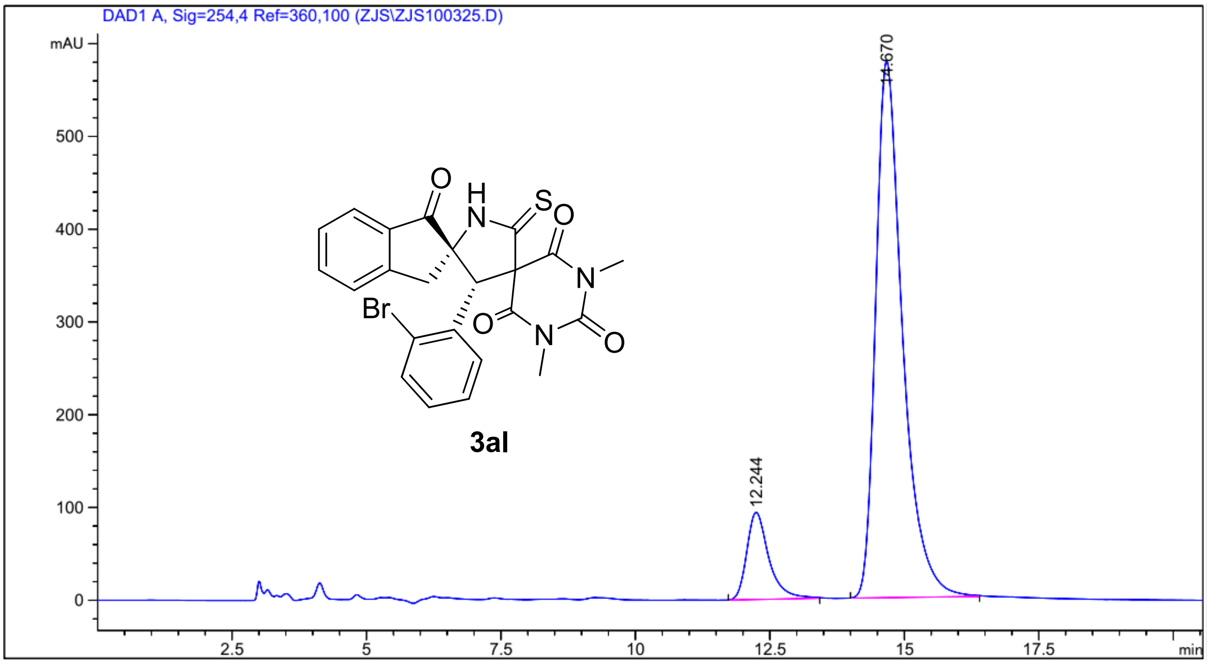

Peak #	RetTime [min]	Type	Width [min]	Area [mAU*s]	Height [mAU]	Area %
1	12.244	BB	0.4297	2687.43677	93.94700	11.7733
2	14.670	BB	0.5222	2.01391e4	578.40143	88.2267

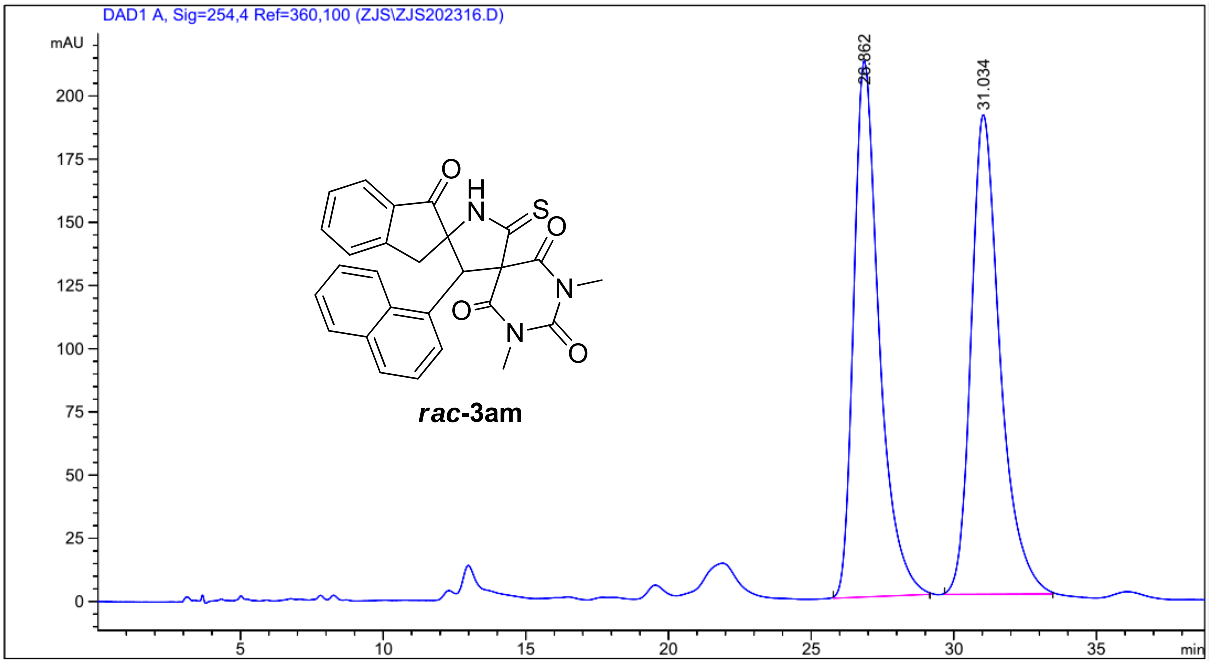

Peak #	RetTime [min]	Type	Width [min]	Area [mAU*s]	Height [mAU]	Area %
1	26.862	BB	0.9259	1.31642e4	212.15480	49.4879
2	31.034	BB	1.0684	1.34367e4	189.69574	50.5121

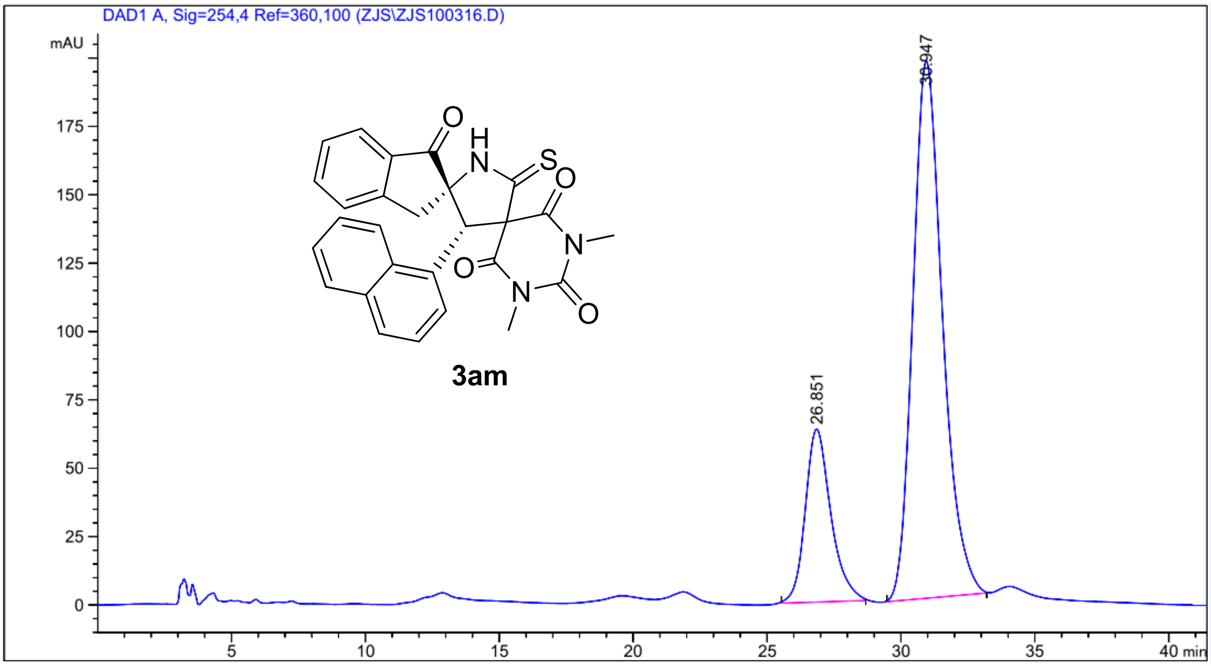

Peak #	RetTime [min]	Type	Width [min]	Area [mAU*s]	Height [mAU]	Area %
1	26.851	BB	0.9627	4210.24951	63.23738	21.7440
2	30.947	BB	1.1621	1.51526e4	196.40216	78.2560

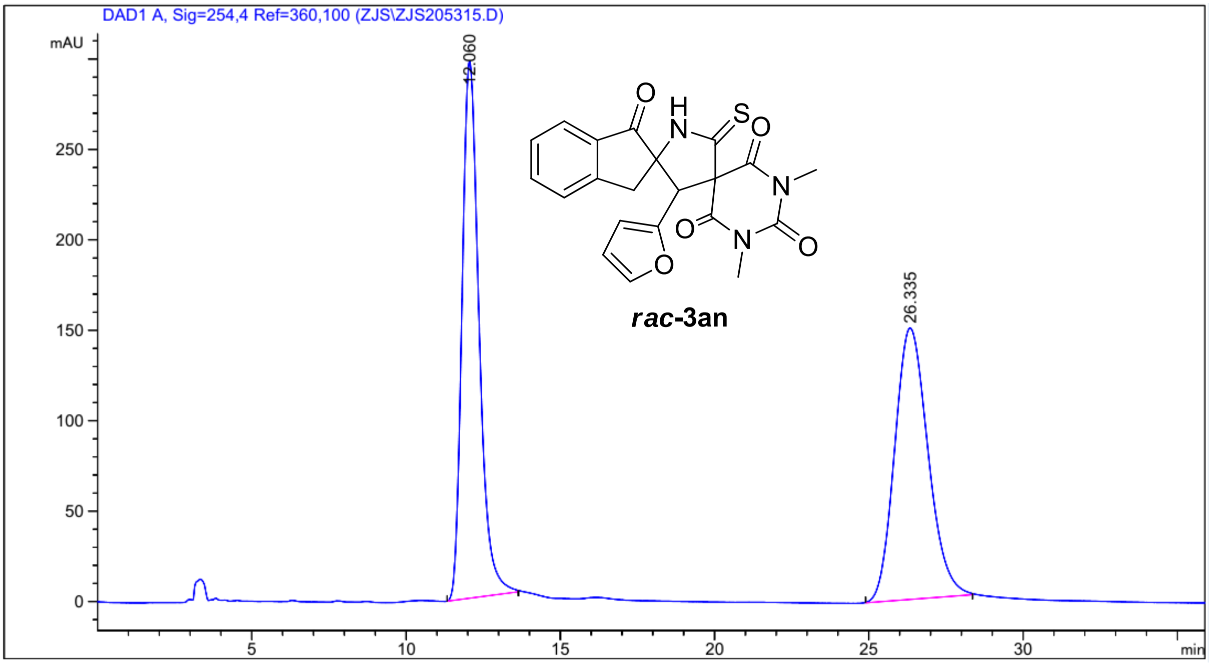

Peak #	RetTime [min]	Type	Width [min]	Area [mAU*s]	Height [mAU]	Area %
1	12.060	BB	0.5904	1.13691e4	297.11658	50.1848
2	26.335	BB	1.1651	1.12854e4	150.10611	49.8152

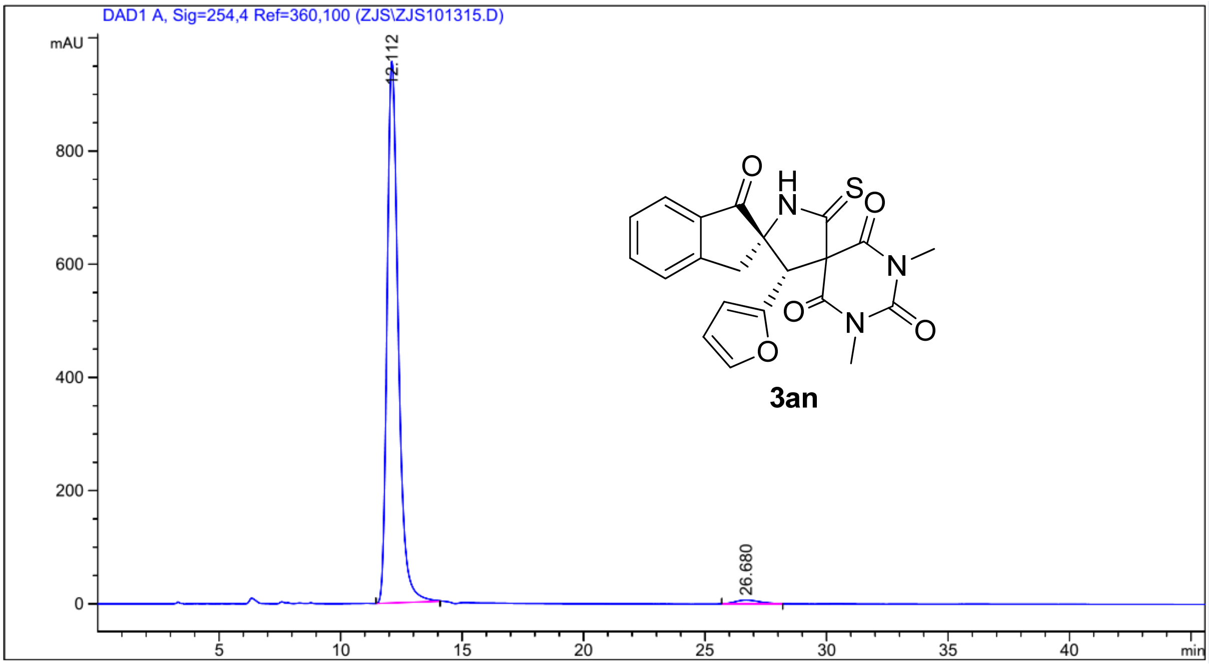

Peak #	RetTime [min]	Type	Width [min]	Area [mAU*s]	Height [mAU]	Area %
1	12.112	BB	0.4918	3.06693e4	957.24963	98.4780
2	26.680	BB	0.8381	473.98697	6.70979	1.5220

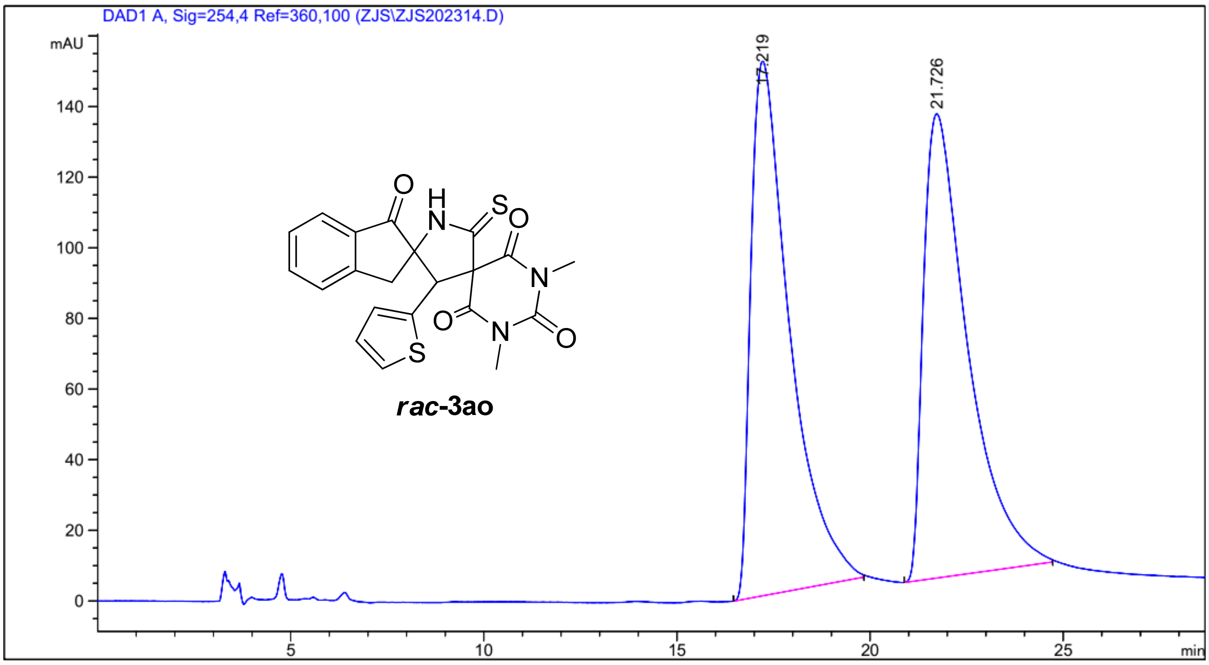

Peak #	RetTime [min]	Type	Width [min]	Area [mAU*s]	Height [mAU]	Area %
1	17.219	BB	1.0225	1.06188e4	151.34993	50.2965
2	21.726	BB	1.1594	1.04936e4	131.44218	49.7035

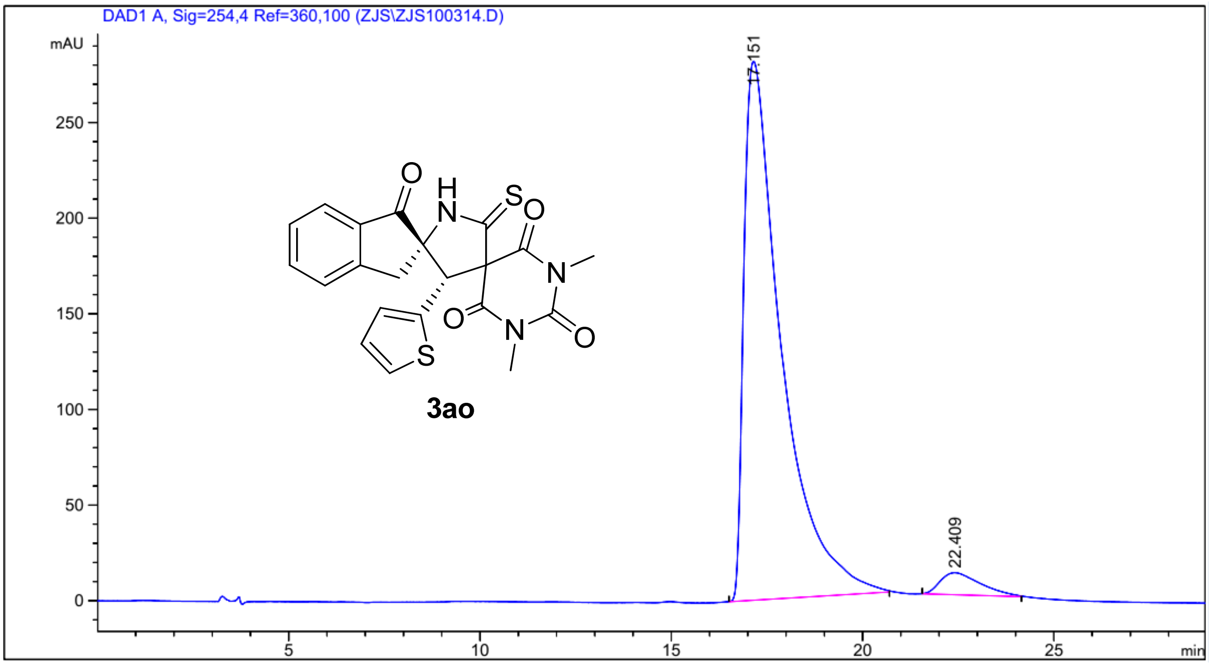

Peak #	RetTime [min]	Type	Width [min]	Area [mAU*s]	Height [mAU]	Area %
1	17.151	BB	0.9918	1.92646e4	281.71320	95.8100
2	22.409	BB	0.8811	842.48981	11.49396	4.1900

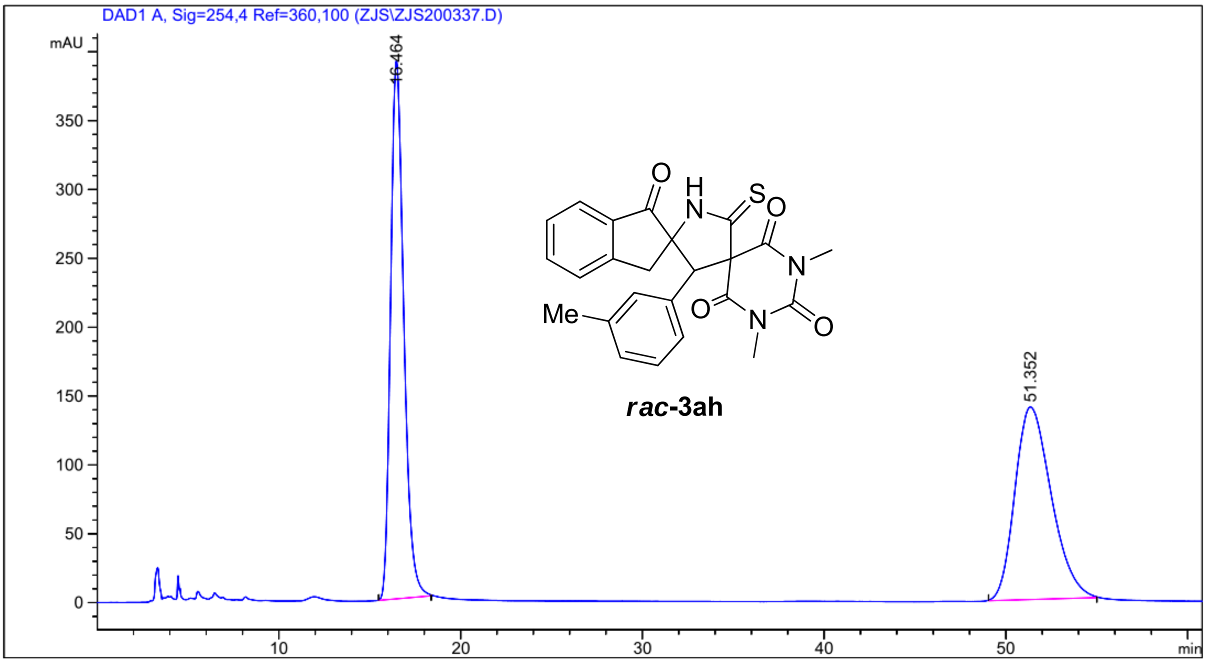

Peak #	RetTime [min]	Type	Width [min]	Area [mAU*s]	Height [mAU]	Area %
1	16.464	BB	0.7721	1.96879e4	390.59589	50.3143
2	51.352	BB	1.8564	1.94419e4	139.80742	49.6857

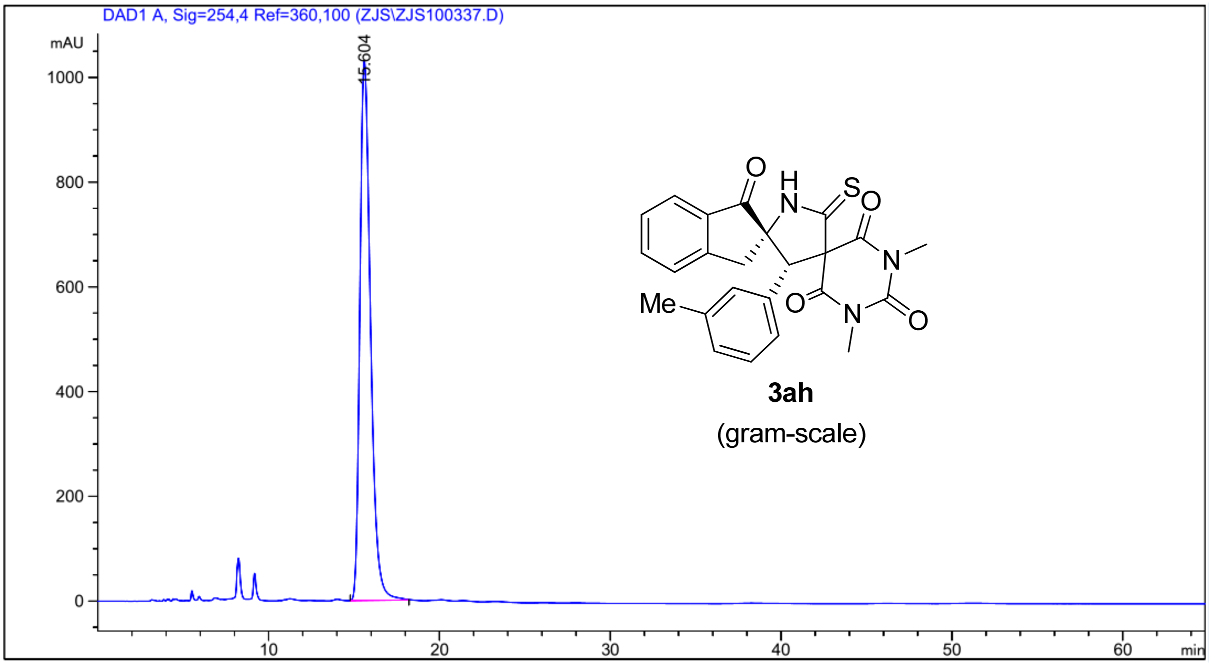

Peak #	RetTime [min]	Type	Width [min]	Area [mAU*s]	Height [mAU]	Area %
1	15.604	BB	0.6679	4.46888e4	1030.59180	100.0000

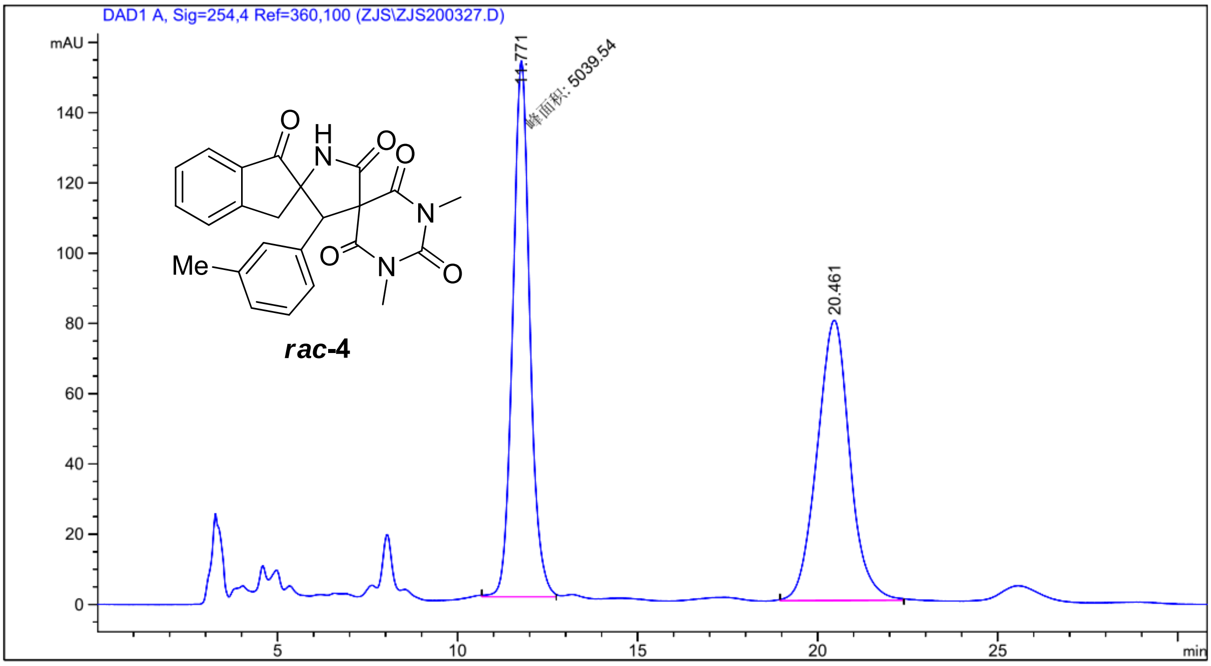

Peak #	RetTime [min]	Type	Width [min]	Area [mAU*s]	Height [mAU]	Area %
1	11.771	MM	0.5503	5039.53711	152.61723	50.0416
2	20.461	BB	0.9556	5031.16309	79.79497	49.9584

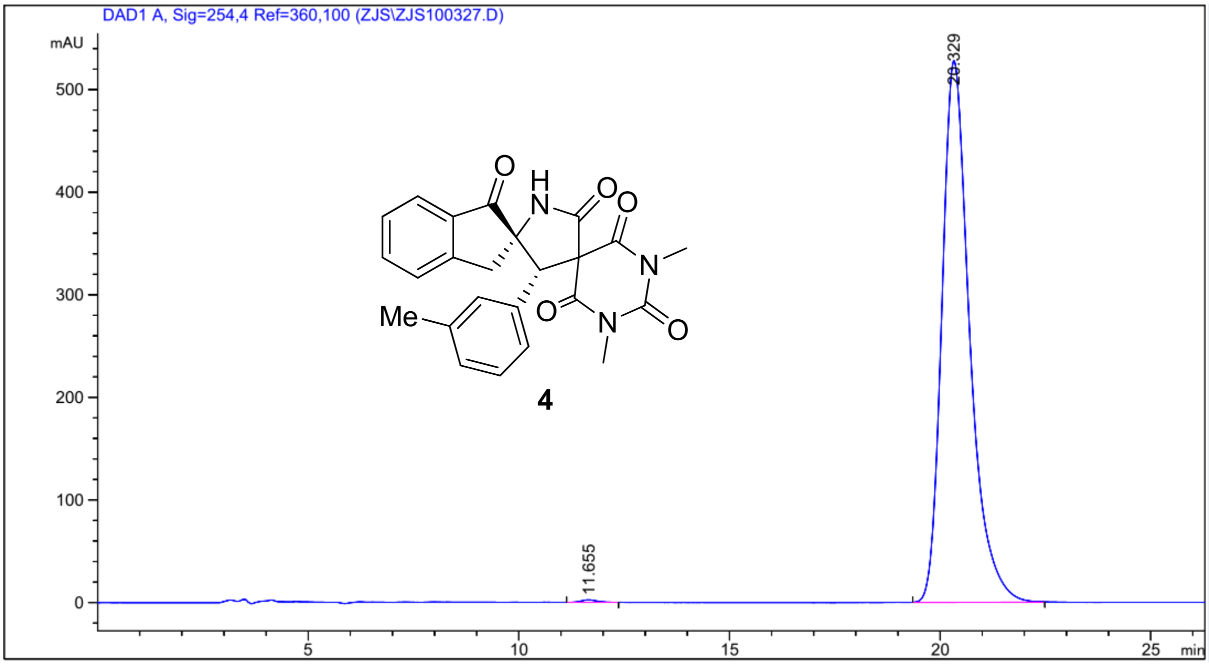

Peak #	RetTime [min]	Type	Width [min]	Area [mAU*s]	Height [mAU]	Area %
1	11.655	BB	0.3961	63.60685	2.40854	0.2623
2	20.329	BB	0.6944	2.41857e4	527.97321	99.7377

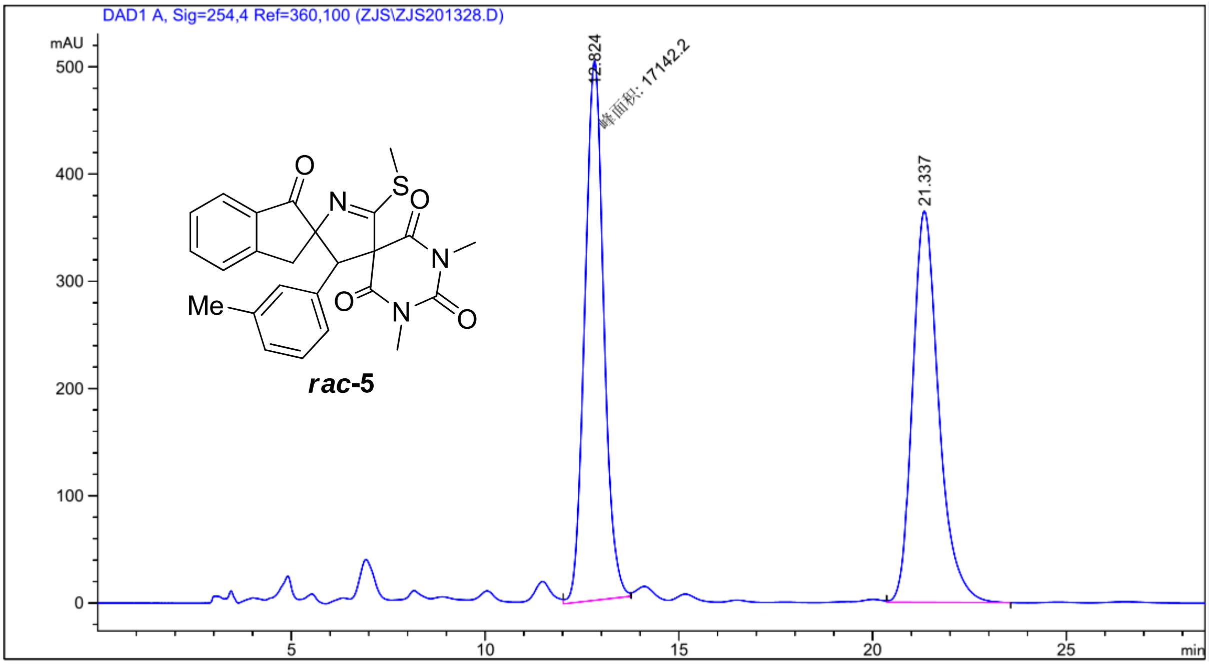

Peak #	RetTime [min]	Type	Width [min]	Area [mAU*s]	Height [mAU]	Area %
1	12.824	MM	0.5679	1.71422e4	503.06976	49.9395
2	21.337	VB	0.7176	1.71837e4	364.75650	50.0605

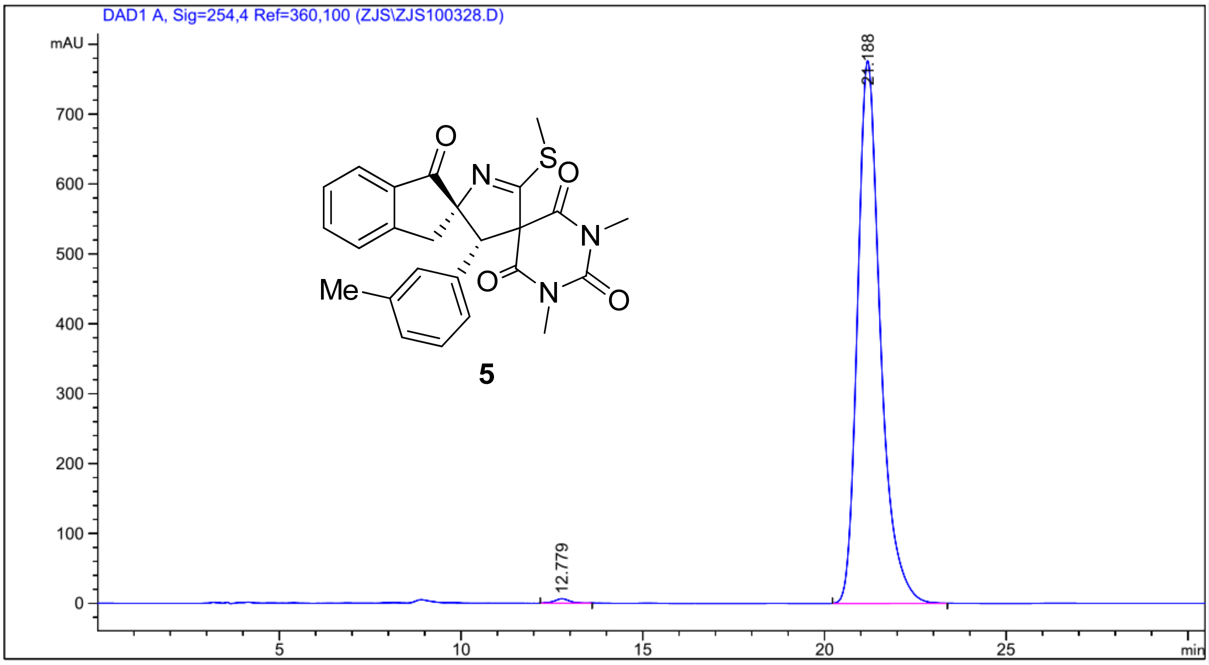

Peak #	RetTime [min]	Type	Width [min]	Area [mAU*s]	Height [mAU]	Area %
1	12.779	BB	0.4493	194.42592	6.49310	0.5675
2	21.188	BB	0.6599	3.40679e4	776.44232	99.4325

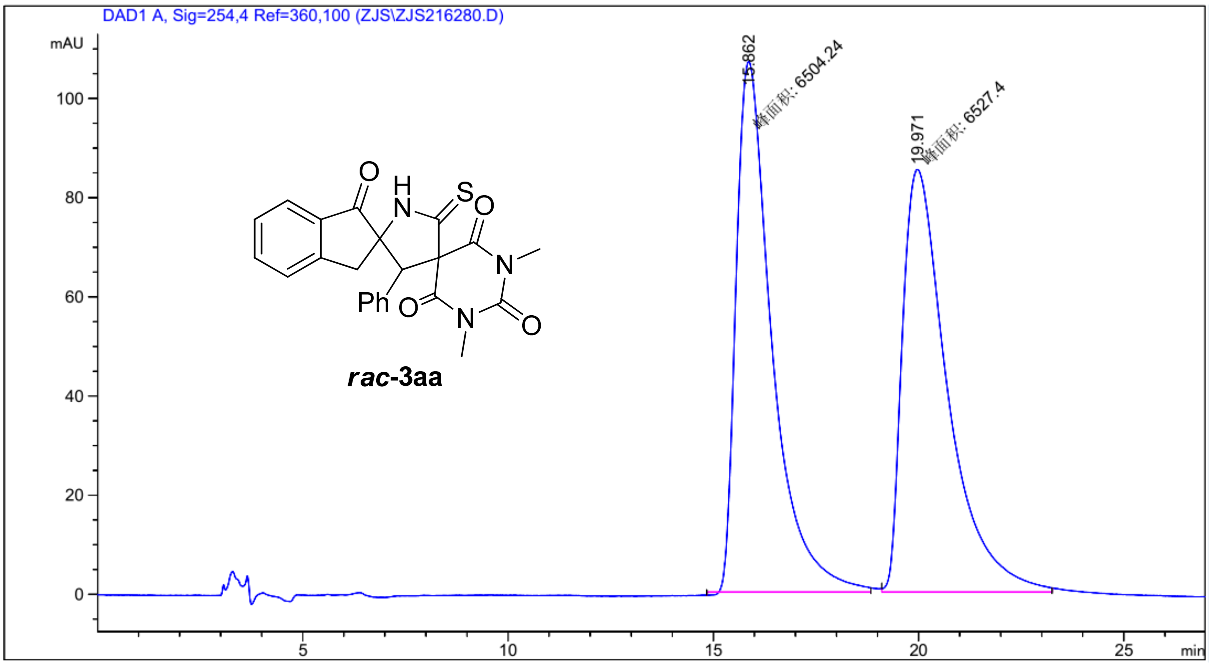

Peak #	RetTime [min]	Type	Width [min]	Area [mAU*s]	Height [mAU]	Area %
1	15.862	MM	1.0129	6504.24268	107.02784	49.9112
2	19.971	MM	1.2765	6527.39893	85.22320	50.0888

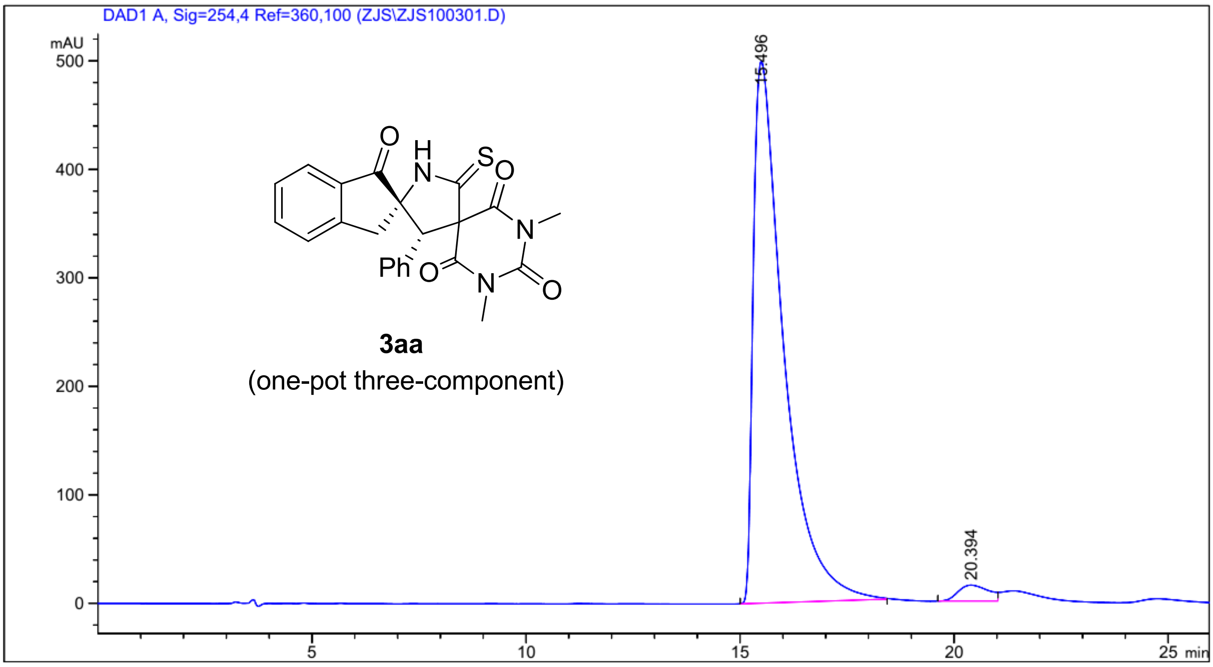

Peak #	RetTime [min]	Type	Width [min]	Area [mAU*s]	Height [mAU]	Area %
1	15.496	BB	0.7332	2.47100e4	499.46423	97.1403
2	20.394	BV	0.6404	727.43628	14.72435	2.8597

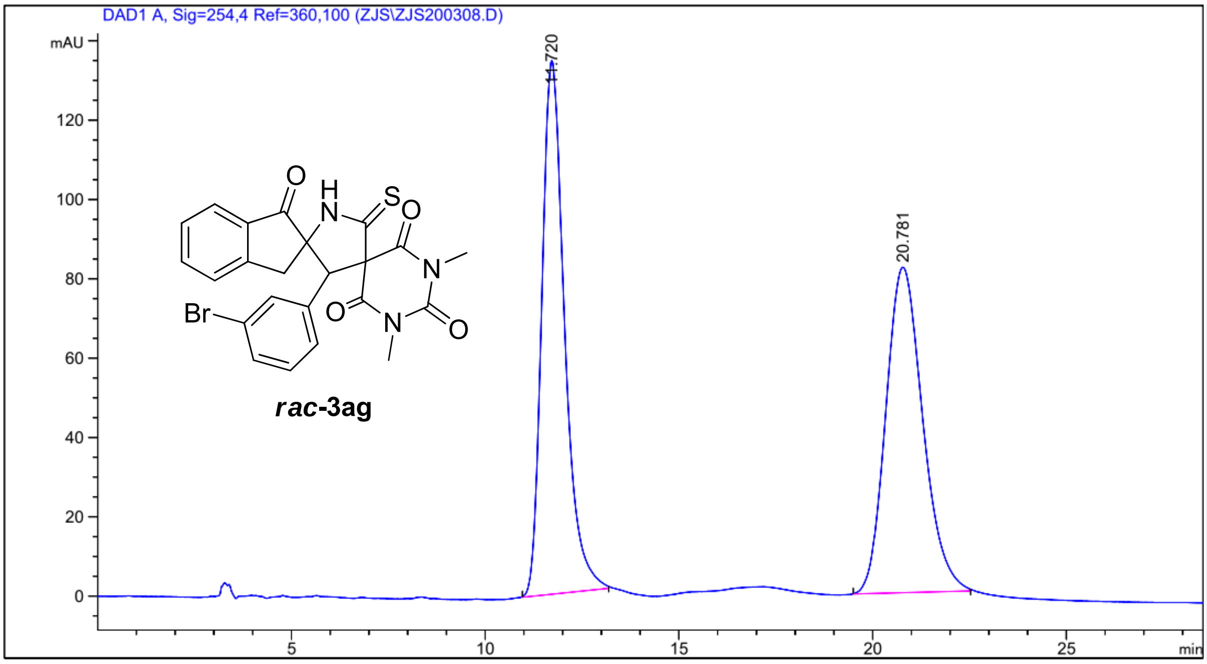

Peak #	RetTime [min]	Type	Width [min]	Area [mAU*s]	Height [mAU]	Area %
1	11.720	BB	0.6210	5475.66895	134.50122	50.2172
2	20.781	BB	1.0038	5428.31006	82.05406	49.7828

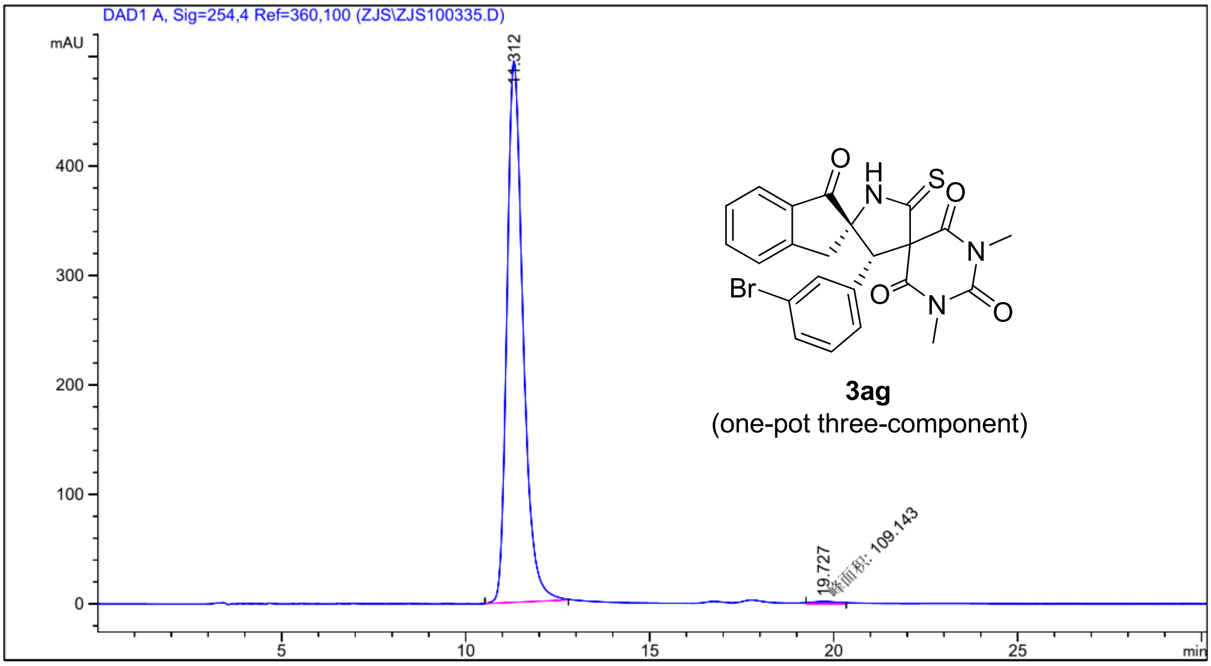

Peak #	RetTime [min]	Type	Width [min]	Area [mAU*s]	Height [mAU]	Area %
1	11.312	BB	0.4647	1.51225e4	494.66156	99.2834
2	19.727	MM	0.8546	109.14272	2.12848	0.7166
